# A General Enzymatic Strategy for Site‐Specific Incorporation of Modified Genetic Building Blocks Into DNA

**DOI:** 10.1002/advs.75917

**Published:** 2026-06-02

**Authors:** Raveena Raveena, Bhavana Ramadas, Sidney Becker

**Affiliations:** ^1^ Max‐Planck Institute of Molecular Physiology Dortmund Germany; ^2^ Faculty for Chemistry and Chemical Biology Technical University Dortmund Dortmund Germany

**Keywords:** chemistry, dna, dna sequencing, enzymatic dna synthesis, polymerase kinetics, labelling, modified nucleotide, site‐specific incorporation, engineered polymerase, specific labelling

## Abstract

Methods for site‐specific incorporation of modified DNA building blocks remain limited. Phosphoramidite chemistry, the most common approach, suffers from low coupling efficiency, error‐prone synthesis, and toxic waste generation. Enzymatic approaches on the other hand operate under mild aqueous conditions but are highly context‐dependent, cannot incorporate multiple modifications, or lack generalizability. Here, we present a proof‐of‐concept for a general enzymatic strategy that uses template‐dependent polymerases to incorporate modified nucleotides at defined positions with high fidelity, providing highly pure products through intrinsic error correction. There is scope for this approach to be automated on DNA sequencers using Sequencing‐by‐Synthesis (SBS) technology, offering a pathway for a routine, eco‐friendly synthesis of long, site‐specifically modified DNA sequences, including promoters and gene fragments.

## Graphical abstract

Eco‐friendly enzymatic synthesis of high‐fidelity, site‐specifically modified DNA using 3′‐blocked nucleotides for full synthetic control and next‐generation sequencing compatibility.

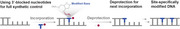

## 1 Introduction

The genome contains numerous modified nucleotides that perform diverse biological functions. These modifications can be broadly classified into epigenetic marks and DNA lesions. Epigenetic modifications, such as 5‐methylcytosine (5mC), regulate essential processes including gene expression, development, and genomic imprinting [1, 2, 3]. In contrast, stress‐induced lesions such as 8‐oxo‐purines arise from oxidative damage caused by metabolic activity or environmental stressors [4, 5, 6] and serve as important biomarkers [7]. These lesions are highly mutagenic due to their propensity to mispair during replication, and insufficient repair can lead to genome instability and cancer. Notably, 8‐oxoguanine (8oxoG) has also been shown to influence transcription, suggesting a potential regulatory role [8, 9, 10, 11].

The ability to precisely control the site‐specific incorporation of modified nucleotides would enable powerful applications in synthetic biology, biotechnology, gene therapy and epigenetic regulation. However, broadly applicable methods for efficiently synthesizing DNA fragments with defined genetic modification profiles, including epigenetic marks, DNA lesions or synthetic functionalities such as fluorophores or affinity probes, remain limited. At present, phosphoramidite chemistry is the dominant strategy for site‐specific incorporation [12]. While this approach enables *de novo* DNA synthesis, its coupling efficiency (∼99.5%) restricts practical oligonucleotide lengths to approximately 200 nucleotides and leads to substantial side‐product formation with additional errors arising from acidic detritylation that promotes depurination. The reliance on organic solvents generates large quantities of toxic waste (1000 kg per kilogram of oligonucleotide) [13]. To generate longer modified DNA constructs, purified oligonucleotides must be assembled enzymatically [14, 15, 16, 17, 18, 19]. However, these downstream enzymatic steps often fail when applied to long or repetitive sequences or to regions with highly biased base compositions. Moreover, such assembly steps are limited by context‐dependent specificity, preventing modification at arbitrary positions within a DNA sequence.

Enzymatic synthesis of site‐specifically modified DNA may offer an attractive alternative, as it operates under mild aqueous conditions, avoids toxic waste, requires no harmful deprotection and can achieve high coupling efficiencies with minimal side‐product formation. Early efforts using terminal deoxynucleotidyl transferase (TdT) provided limited positional control and allowed incorporation of only a single modification due to poor tolerance for modified substrates [20]. Other polymerase‐based strategies face similar constraints, as low incorporation efficiencies, polymerase stalling and reduced control over the synthesis process limits their robustness, and in practice often restrict incorporation to a limited number of modified nucleotides [21, 22, 23, 24, 25, 26, 27]. While some of these studies provide the scope for site specific incorporation of multiple modifications, they typically require rigorous sequence design and additional steps [22, 26, 27]. More recently, CRISPR‐Cas systems have been adapted to target DNA methyltransferases to a user‐defined DNA sequence for site‐specific cytosine methylation [28, 29, 30, 31, 32, 33, 34, 35]. However, these approaches are prone to off‐target activity and are not readily generalizable for incorporating diverse modified genetic building blocks.

**FIGURE 1:**
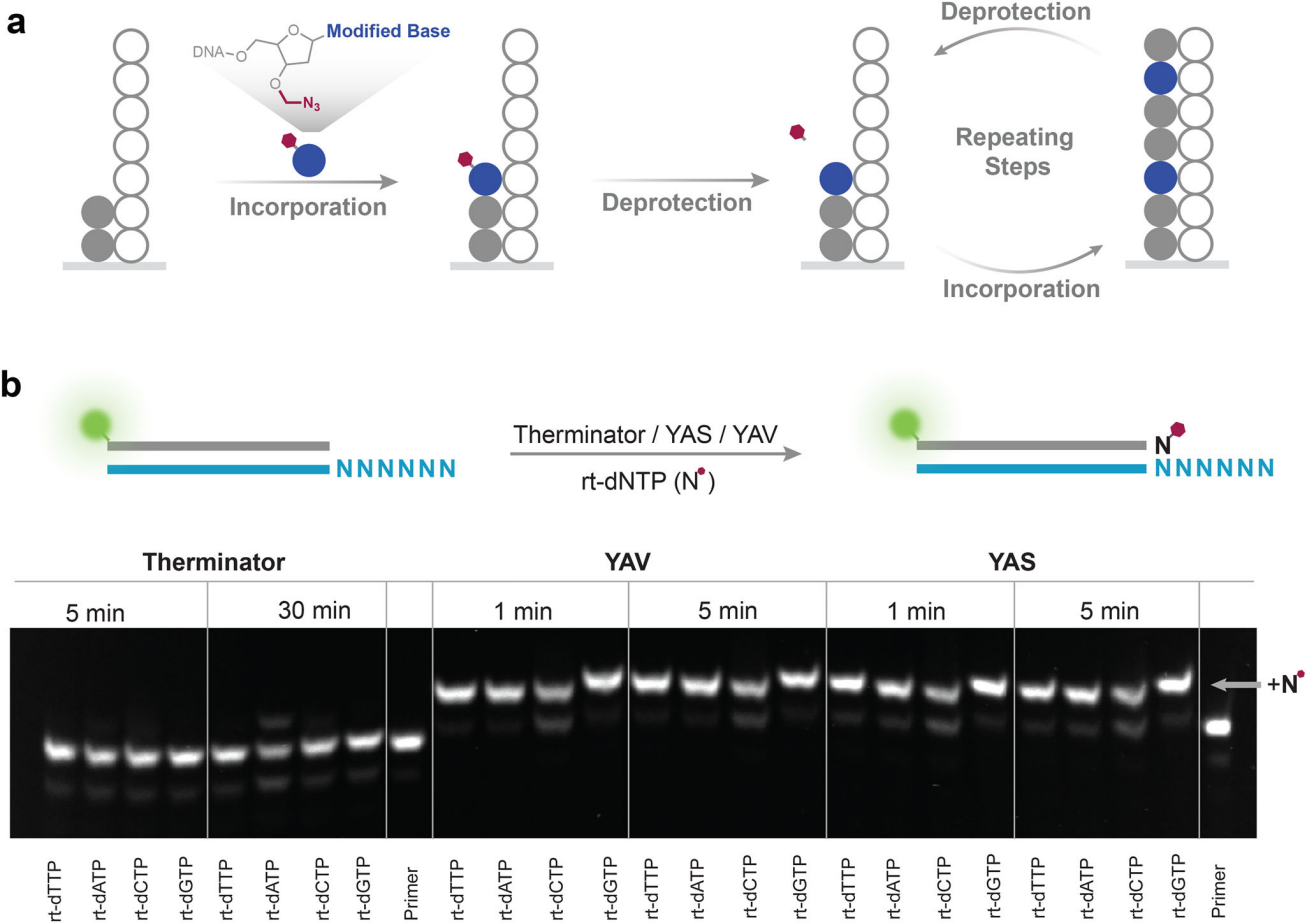
(a) General enzymatic approach for the controlled, site‐specific incorporation of modified genetic building blocks into DNA by template‐dependent polymerases. (b) Urea‐PAGE gel for single nucleotide incorporation of rt‐dNTPs in a primer with 5’ 6FAM opposite six A/T/G/Cs with Therminator, YAV and YAS polymerases at two incubation times. Reactions were incubated in 20 µL volumes at 60°C with 1U/20 µL polymerase and 10 µm rt‐dNTP (N: A/T/G/C, red hexagon: reversible terminator (rt)).

To our knowledge, no generalizable enzymatic method currently enables the site‐specific incorporation of multiple modified nucleotides into DNA. We hypothesized that template‐dependent polymerases may enable controlled, high‐fidelity incorporation of diverse modified building blocks through primer extension opposite a given template. To achieve controlled stepwise synthesis, we employed reversible 3′‐O blocking groups (“reversible terminators”), commonly used in template‐independent enzymatic DNA synthesis or next‐generation sequencing [36, 37]. We selected a template‐dependent polymerase capable of efficiently incorporating reversible terminator nucleotide triphosphates (rt‐dNTPs). After optimizing incorporation and deprotection conditions, we achieved efficient multi‐cycle primer extension. To show the site‐specific incorporation of modified DNA building blocks, we synthesized reversible terminator triphosphates of 8‐oxoguanine (8oxoG), 8‐oxoadenine (8oxoA), and 5‐methylcytosine (5mC). These building blocks were incorporated enzymatically at defined positions with exceptionally high coupling efficiency (>99.9% stepwise yield). This strategy establishes a robust, broadly applicable proof‐of‐concept for site‐specific enzymatic incorporation of diverse DNA modifications into DNA at high‐fidelity with low side product formation (Figure 1a). The (bio)chemistry is fully compatible with sequencing‐by‐synthesis (SBS) technology, providing a path toward automated, high‐fidelity, eco‐friendly synthesis of long, site‐specifically modified DNA constructs without the need for laborious assembly steps.

## 2 Results

### 2.1 Polymerase Incorporation of rt‐dNTPs

Our initial focus was to identify a polymerase capable of efficiently incorporating rt‐dNTPs. While Therminator polymerase (NEB), a mutant of Thermococcus 9°N‐7 DNA polymerase, can incorporate a variety of modified dNTPs, its efficiency with rt‐dNTPs is low (Figure 1b). Improved polymerases capable of efficient incorporation of dNTPs with a 3’‐O‐azidomethyl group at the sugar moiety requires introduction of mutations in the catalytic site of the *Thermococcus* 9°N‐7 DNA polymerase (amino acids 408–410 and 484–486) in addition to the mutations in the 3’‐5’ exonuclease site (D141A, E143A) [38]. Therefore, we introduced mutations in the Addgene plasmid (pOpen‐9N7polA (CT), #165502) using Gibson Assembly, and inserted the gene construct into the expression vector pET‐16b via restriction‐enzyme cloning. BL21 (DE3) RIL Codon Plus cells (Agilent technologies) were transformed with the plasmids, and the gene constructs were expressed and purified (Figure S1). We tested different mutant versions to identify polymerases that were able to efficiently incorporate rt‐dNTPs with 3’‐O‐azidomethyl groups, and found that the L408**Y**/Y409**A**/P410**S**/A485L and L408**Y**/Y409**A**/P410**V**/A485L (from now on referred to as the **YAS** and **YAV** polymerases) combinations proved to be beneficial. The activity (units) of the purified polymerases YAS & YAV was quantified by real‐time qPCR using the EvaEZ Fluorometric Polymerase Activity Assay kit.

We performed enzymatic incorporation assays using the purified polymerases, to assess their ability to efficiently incorporate rt‐dNTPs (rt‐dATP, rt‐dTTP, rt‐dGTP, rt‐dCTP), and to confirm single‐nucleotide incorporation mediated by the 3’‐O‐azidomethyl reversible terminator. A 5’‐FAM‐labelled primer (Primer_1, 24 nt) was extended over various templates (30 nt) containing six consecutive thymines (6T_Template), adenines (6A_Template), cytosines (6C_Template), or guanines (6G_Template), at the 3′ end to detect potential read‐through resulting from incomplete termination (Table S1). Such effects may arise from dNTP contamination or instability of the reversible terminator. Reaction products were analysed on 20% denaturing urea‐PAGE gels. Assays were performed with Therminator, YAS and YAV polymerases (1U per 20 µL reaction) (Figure 1b). While the Therminator polymerase showed poor incorporation efficiency, YAS and YAV polymerases achieved nearly complete single‐nucleotide incorporation of all rt‐dNTPs. Importantly, we observed only single‐nucleotide incorporation, indicating that the rt‐dNTPs contained no dNTP contamination and that the 3′‐O‐azidomethyl group was stable under the reaction conditions. These validations are crucial for reducing potential side products that may arise during primer extension with multiple incorporation cycles.

### 2.2 Optimizing Incorporation & Deprotection of rt‐dNTPs on Streptavidin Beads

**FIGURE 2:**
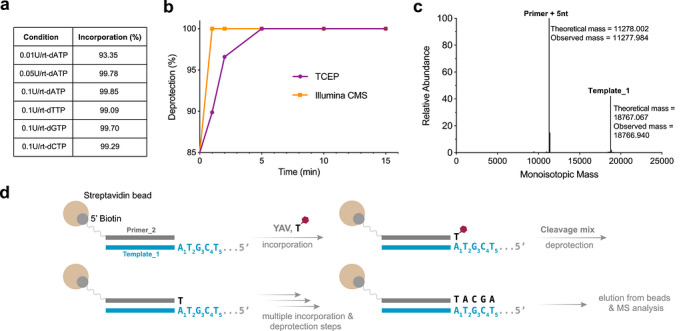
(a) Optimizing incorporation conditions of rt‐dNTPs with the YAV polymerase on streptavidin beads at 60°C for 30 min. The amount of polymerase and respective rt‐dNTP is provided together with the incorporation efficiency determined by LC‐MS. (b) Optimizing & comparing deprotection conditions of TCEP and Illumina cleavage mix (CMS) on streptavidin beads. (c) LC‐MS analysis after five iterative extension cycles (incorporation & deprotection). (d) Schematic representation of stepwise incorporation of dNTPs. rt‐dNTPs are incorporated by YAV, followed by deprotection with Illumina CMS for stepwise incorporation of dNTPs.

As our workflow would require multiple steps of incorporation and deprotection (removal of the reversible terminator), we proceeded to optimize reaction conditions on a solid support. For this purpose, a 5’‐biotinylated primer (Primer_2) was annealed to a template (Template_1) and the duplex was bound to streptavidin beads (Table S1). We first optimized the conditions for single rt‐dNTP incorporation on the beads. Because site‐specific incorporation requires multiple sequential steps, our goal was to minimize polymerase usage while still achieving efficient incorporation at 60°C. Although 9°N polymerase variants exhibit optimal activity at 75°C, we performed the reactions at a reduced temperature with extended incubation to avoid streptavidin dissociation or denaturation. To establish suitable conditions, we evaluated rt‐dATP incorporation using 0.01 U, 0.05 U, and 0.1 U YAV polymerase per 20 µL reaction, incubated for 30 min at 60°C. Nearly complete incorporation (∼99.9%) was obtained with 0.1 U. Using this condition, we next tested the remaining rt‐dNTPs and observed >99% incorporation for all four nucleotides (Figure 2a).

Next, we compared the deprotection rates of the reversible terminator using TCEP and Illumina cleavage mix (CMS) that is commonly part of Miseq sequencing kits. We first incorporated rt‐dTTP, followed by deprotection with 200 mM TCEP or CMS mix, by incubating at 65°C (TCEP) or 60°C (CMS) for different time intervals. The Illumina cleavage mix immediately shows quantitative deprotection after 1 min, while TCEP takes around 5 min to obtain complete deprotection (Figure 2b).

After confirming efficient incorporation and deprotection, we proceeded to extend the primer through iterative cycles of rt‐dNTP addition. The rt‐dNTPs were added individually using YAV polymerase, followed by removal of the 3′‐O‐azidomethyl reversible terminator with the CMS mix, enabling controlled stepwise extension. Upon completion of all cycles, the oligonucleotides were eluted from the beads and analysed by MS (Figure 2c,d). Incorporation efficiency was determined from the relative abundance of the final primer‐extension product compared to all incomplete products, as calculated using BioPharma Finder. We initially attempted primer extension under the previously optimized conditions, but these yielded only ∼96.4% overall efficiency for multiple incorporations (Table S2). To improve efficiency for extension of five nucleotides, we increased the polymerase concentration to 0.5 U/20 µL YAV polymerase, achieving ∼99% of the expected product (primer + 5 nt) (Table S3).

### 2.3 Synthesis of rt‐8oxoG, rt‐8oxoA & rt‐5mC Triphosphates

Next, we aimed to achieve site‐specific incorporation of biologically relevant modified nucleotides. To illustrate the range of substrates amenable to site‐specific incorporation, we synthesized 5mC (epigenetic base) as well as 8oxoG, and 8oxoA (both oxidative lesions) as triphosphates containing a reversible terminator.

**SCHEME 1:**
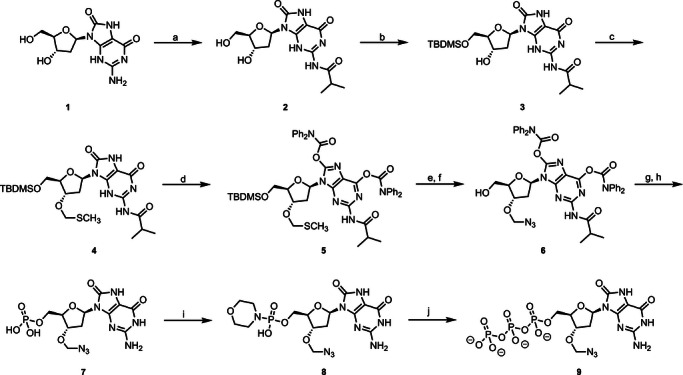
Synthesis of 3'‐O‐azidomethyl‐8‐oxo‐dGTP 9 from 8‐oxo‐dG 1. a) TMSCl, Isobutyric anhydride, Pyridine, room temperature, 6 h, 60%, (b) TBDMSCl, Pyridine, room temperature, 3.5 h, 78%, (c) DMSO, AcOH, Ac_2_O, room temperature, 48 h, 79%, (d) Ph_2_NCOCl, DIPEA, Pyridine, room temperature, 4 h, 90%, (e) TMSN_3_, NIS, TfOH, DCM, 0°C, 4.5 h (f) NH_4_F, MeOH, room temperature, 22 h, 77% over 2 steps, (g) POCl_3_, PO(OMe)_3_, Proton‐sponge, 0°C, 2 h (h) 7N NH_3_ in MeOH, 60°C, 60 h, 42%, (i) DCC, Morpholine, t‐BuOH:H_2_O(1:1), 95°C, 4.5 h, 79% and (j) (Bu_3_NH)_4_P_2_O_7_, Tetrazole, DMF, 37°C, 20 h, 56%.

For the synthesis of 3′‐O‐azidomethyl‐8‐oxo‐dG triphosphate **9**, we first protected the reactive functional groups of commercially available 8‐oxo‐dG **1** to prevent cross‐reactivity during 3′‐O‐reversible terminator synthesis (Scheme 1). The 2‐amino functional group on **1** was protected by treatment with isobutyric anhydride in the presence of trimethylchlorosilane to obtain **2** [39]. This was followed by TBDMSCl (tert‐butyldimethylchlorosilyl) protection of 5’‐OH, which resulted in protected nucleoside **3** [39]. For introducing the azidomethyl group on the 3’‐OH position, Pummerer rearrangement conditions were used to prepare the active 3'‐O‐methylthiomethyl **4** from **3** [40, 41]. However, unlike deoxyguanosine, 8oxo‐guanine consists of two carbonyl functional groups (C‐6 and C‐8) [41]. Therefore, both the carbonyl groups of **4** were protected using diphenylcarbamoyl chloride to achieve protected product **5** in presence of diisopropylethylamine (DIPEA), thereby suppressing possible side reactions. NIS/TfOH activation [42] successfully activated 3'‐O‐methylthiomethyl, which was converted to 3'‐O‐azidomethyl using TMSN_3_ as azide source [43]. These conditions, provided reversible terminator nucleoside **6** after silyl deprotection with NH_4_F as fluoride source. Importantly, under these conditions we were unable to identify remaining **4** which would lead to chain termination as the 3'‐O‐methylthiomethyl moiety cannot be readily deprotected.

Converting **6** to **9** was accomplished by performing the monophosphorylation of 3’‐O‐azidomethyl modified nucleoside **6** followed by activated morpholidate formation. Monophosphate **7** was synthesised using POCl_3_ in trimethyl phosphate in the presence of proton sponge to eliminate pH‐imbalance during the reaction [44]. Subsequently the protected monophosphate was deprotected under basic conditions, resulting in monophosphate **7** [45]. To avoid 3’‐O‐azidomethyl cleavage during morpholidate formation, the synthesis was carried out under mild conditions by slowly treating **7** with DCC (dicyclohexylcarbodiimide) in presence of morpholine to attain morpholidate **8** [46], which was further converted into the triphosphate **9** with tetrabutylammonium pyrophosphate in presence of tetrazole activator [47]. HPLC purification provided the 3’‐O‐azidomethyl‐8‐oxo‐dGTP (rt‐8oxoG) **9** as triethylammonium salt with 56% isolated yield. Triphosphate **9** was achieved with 4.7% overall yield via 10 synthetic steps.

For the synthesis of 3’‐O‐azidomethyl‐8‐oxo‐deoxyadenosine triphosphate we followed a similar synthetic pathway (Scheme S1). Briefly, we started from 8‐bromo‐dA **10**, which was treated with in‐situ prepared sodium benzyloxide to obtain 8‐benzyloxy‐dA **11** [48]. The 6‐amino group was benzoyl protected, and further treated with acidic condition giving N‐benzoylated 8‐oxo‐dA **12** [49], after loss of the 8‐benzyl moiety [50]. 5’‐TBDMS protection afforded product **13**, which was further converted by Pummerer rearrangement to obtain 3’‐O‐methylthiomethyl modified product **14**. We found that the 8‐oxo group did not require protection for conversion to the 3′‐O‐azidomethyl moiety via NIS/TfOH activation, provided the reaction was carried out at low temperature (−40 °C). Deprotection of 5’‐TBDMS finally afforded 3’‐O‐azidomethyl‐8‐oxo‐6‐benzoyl‐dA **15**, which was further converted into the monophosphate **16**. After deprotection of the benzoyl moiety the phosphate was activated as morpholidate to obtain compound **17**, which was further converted into the triphosphate **18** as triethylammonium salt in 63% isolated yield. We achieved the synthesis of triphosphate **18** via 12 synthetic steps giving 5.0% overall yield.

The epigenetic nucleobase 5‐methyl‐deoxycytidine (5mdC) **19** was converted to its reversible terminator nucleotide 3’‐O‐azidomethyl‐5‐methyl‐deoxycytidine triphosphate **26** by following a similar synthetic pathway (Scheme S2). We proceeded synthesis by protecting the 6‐amino group of **19** with benzoyl chloride to obtain **20** [51]. 3’‐O‐methylthiomethyl product **22** was synthesized by Pummerer rearrangement on the 5’‐TBDMS protected product **21**. Finally, 3’‐O‐azidomethyl moiety **23** was achieved with NIS/TfOH activation under low temperature (‐20°C) followed by subsequent deprotection of 5’‐TBDMS. Nucleoside **23** was phosphorylated and deprotected to obtain the monophosphate **24**, which was further activated as morpholidate **25**. The active morpholidate was converted into the triphosphate **26** as triethylammonium salt in 46% isolated yield over two‐steps. Triphosphate **26** synthesis was attained with 1.4% overall yield via 10 steps. There is scope to improve the yield by addressing the low‐yielding monophosphorylation step. The protecting groups may hinder efficient phosphorylation due to steric effects. Performing deprotection prior to phosphorylation may be beneficial.

### 2.4 Single‐Nucleotide Incorporation of rt‐8oxoG, rt‐8oxoA & rt‐5mC

**FIGURE 3:**
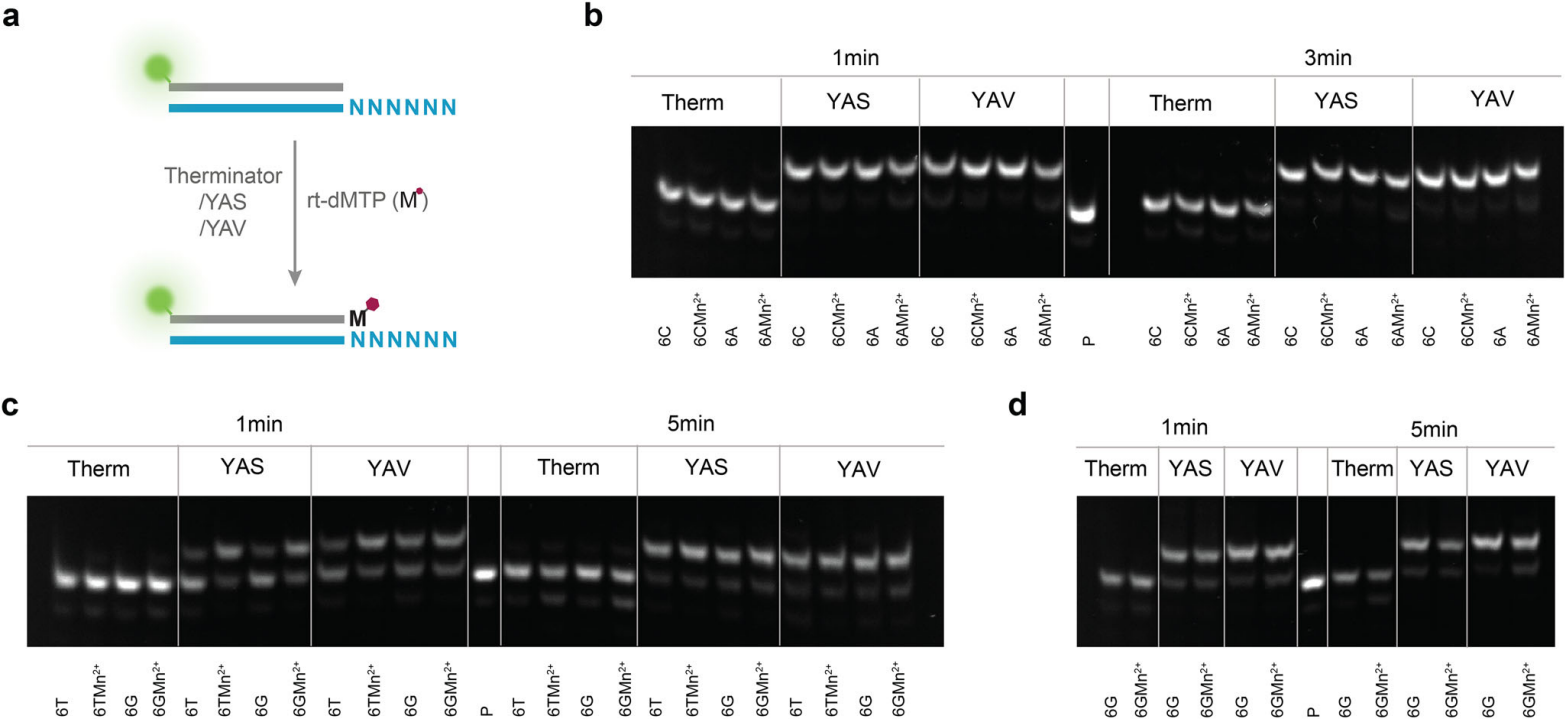
(a) Scheme for single nucleotide incorporation of rt‐dMTP in a primer with 5’ 6‐FAM opposite six A/T/G/Cs where M = 8oxoG/8oxoA/5mC and N = A/T/G/C, with Therminator, YAS and YAV polymerases at two incubation times. (b–d) Urea‐PAGE gels for single nucleotide incorporation (with and without Mn^2+^) of (b) rt‐8oxoG opposite 6C and 6A template (1 and 3 min), (c) rt‐8oxoA opposite 6T and 6G template (1 and 5 min), and (d) rt‐5mC opposite 6G template (1 and 5 min).

Next, we performed enzymatic incorporation assays (Figure 3a) with the synthesized rt‐8oxoG (Figure 3b), rt‐8oxoA (Figure 3c) and rt‐5mC (Figure 3d). Assays were performed with all three polymerases, (1U per 20 µL reaction) with and without Mn^2+^. Mn^2+^ is known to reduce polymerase fidelity and promote incorporation of non‐canonical nucleotides [52, 53]. While the Therminator polymerase showed poor incorporation efficiency as previously observed with natural rt‐dNTPs, YAS and YAV polymerases achieved near quantitative single‐nucleotide incorporation of all three modified rt‐dNTPs. Adding Mn^2+^ had no significant effect on incorporation. Importantly, for all three modified rt‐dNTPs, only single‐nucleotide incorporation was observed, confirming the absence of dNTP contamination in the synthesized rt‐dNTPs.

We also tested incorporation of rt‐dNTP (rt‐dGTP and rt‐8oxoG) to extend a single stranded primer sequence with a commercially available template independent polymerase (TdT). The incorporation, however, was highly inefficient even after 1 h of incubation at 37°C with 200 µM rt‐dNTP (Figure S2), confirming incompatibility of the azidomethyl group with TdT.

**FIGURE 4:**
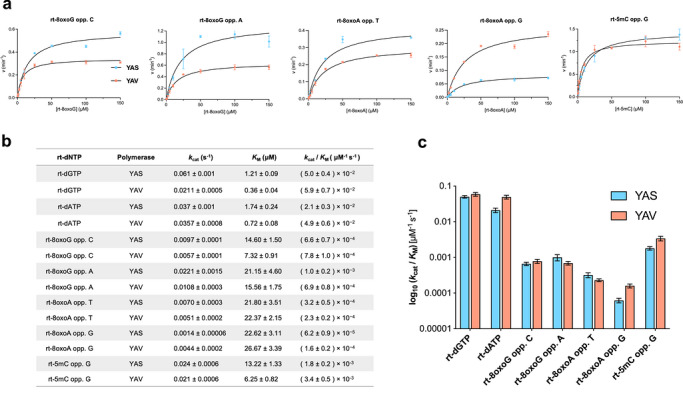
(a) Steady‐state kinetics plots of rt‐8oxoG opposite C & A, rt‐8oxoA opposite T & G, and rt‐5mC opposite G. (b) Results of steady‐state kinetic analysis of incorporation of rt‐dGTP, rt‐dATP, rt‐8oxoG (opposite C & A), rt‐8oxoA (opposite T & G), and rt‐5mC by YAS and YAV. (c) Comparison of log_10_ values of incorporation of rt‐dNTPs by YAS & YAV.

We then proceeded to characterise the kinetics of rt‐dNTP incorporation by the YAS and YAV polymerases, following the protocol of O'Flaherty with minor changes [54]. Primer‐extension assays were performed (in steady state conditions) in duplicates for various rt‐dNTP concentrations. Band intensities after PAGE analysis were used to calculate percentage of product formation, and subsequently turnover values. The rt‐dNTP concentrations and corresponding turnover values were used for Michaelis‐Menten analysis to obtain *k_cat_* and *K_M_* values (Figure 4a; Figure S3). We consistently observed higher *k_cat_* values for the YAS polymerase compared to the YAV polymerase, indicating a higher catalytic rate. However, YAS also exhibited higher *K_M_* values, reflecting lower affinity for rt‐dNTPs, requiring higher rt‐dNTP concentrations (Figure 4b). As expected, the incorporation of the natural rt‐dGTP & rt‐dATP showed higher *k_cat_* and lower *K_M_* values compared to rt‐5mC, rt‐8oxoG and rt‐8oxoA. Additionally, rt‐5mC incorporation gave higher *k_cat_* /*K_M_* values than rt‐8oxoG and rt‐8oxoA (Figure 4c). We also tested the effect of Mn^2+^ on the incorporation efficiency of rt‐dGTP, and surprisingly observed a decrease in the catalytic efficiency of both polymerases (Figure S4). Therefore Mn^2+^ was not considered further.

### 2.5 Site‐Specific Incorporation of 8oxoG, 8oxoA & 5mC in Oligonucleotides

**FIGURE 5:**
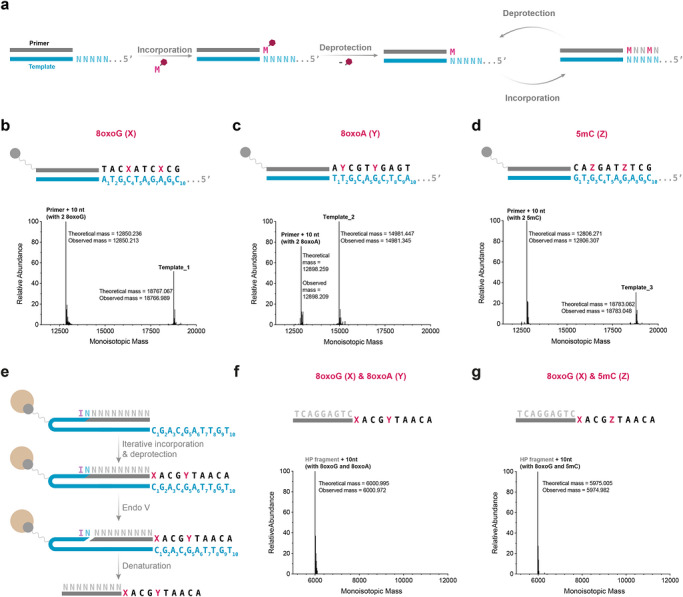
(a) Scheme for site‐specific incorporation of modified nucleotides (M = modified nucleotide (8oxoG/8oxoA/5mC), N = natural nucleotide (A/T/G/C)). (b–d) Site‐specific incorporation of (b) two 8oxoGs, (c) two 8oxoAs, and (d) two 5mCs. 8oxoG is incorporated opposite C_4_ and A_8_ of Template_1, 8oxoA is incorporated opposite T_2_ and G_6_ of Template_2, and 5mC is incorporated opposite G_3_ and G_7_ of Template_3. (e–g) Site‐specific incorporation of two different modifications using an inosine containing hairpin, followed by Endo V treatment and denaturation to obtain pure site‐specifically modified oligonucleotide. (e) Schematic representation of workflow. (f,g) LC‐MS analysis shows clean oligonucleotide with (f) 8oxoG incorporated opposite C_1_ and 8oxoA incorporated opposite G_5_, (g) 8oxoG incorporated opposite C_1_, and 5mC incorporated opposite G_5_.

We subsequently carried out experiments to validate the site‐specific incorporation of 8oxoG, 8oxoA and 5mC into oligonucleotides (Figure 5a). With our previously optimized conditions, we first tested the extension by 5‐nucleotides of Primer_2 opposite Template_1 (8oxoG), Template_2 (8oxoA) or Template_3 (5mC) with the modified nucleotides incorporated at a single site or two specific sites (Figure S5, Tables S4–S8). We obtained near quantitative yield for all three primer extension reactions and proceeded to perform a 10 nucleotide‐extension with 8oxoG incorporated opposite C_4_ and A_8_ of Template_1, 8oxoA incorporated opposite T_2_ and G_6_ of Template_2 and 5mC incorporated opposite G_3_ and G_7_ of Template_3 (Figure 5b–d; Tables S9–S11). We were able to observe the fully extended primer (Primer_2 + 10 nt) as the major product in all cases, with an abundance of ∼99.5% after ten incorporations. This clearly demonstrates the feasibility of the technique for site‐specific incorporation of modified dNTPs into DNA at high efficiency.

We next aimed to demonstrate site‐specific incorporation of two different modifications within a single DNA strand, along with its selective release from the template. To achieve this, we designed a hairpin containing inosine adjacent to the extension site. Following 10 cycles of incorporation and deprotection, the extended oligonucleotide bearing site‐specific modifications was cleaved using Endonuclease V [55, 56] and after denaturation at 80°C, yielded the purified product (Figure 5e). Using this strategy, we synthesized oligonucleotides containing two distinct site‐specific modifications. 8oxoG was introduced together with either 8oxoA or 5mC, and provided clean oligonucleotide after endonuclease V cleavage (Figure 5f,g; Tables S12 and S13). The correct positioning of the modifications was further confirmed by MS/MS analysis (Figures S6 and S7). Notably, the hairpin template may be reused for subsequent rounds of oligonucleotide synthesis.

## 3 Discussion

Here we present a generalizable enzymatic approach for the site‐specific incorporation of modified genetic building blocks into relevant DNA sequences, offering an eco‐friendly alternative to phosphoramidite chemistry. While this strategy does not enable *de novo* DNA synthesis, biologically relevant templates can be readily obtained from natural sources via PCR. We demonstrate site‐specific incorporation of naturally occurring modified DNA building blocks, including the epigenetic base 5‐methylcytosine (5mC) and the DNA lesions 8oxoG and 8oxoA, synthesized as reversible terminator nucleoside triphosphates. Mutants of Thermococcus 9°N‐7 DNA polymerases efficiently incorporated these modified rt‐dNTPs. Site‐specific incorporation was confirmed by primer extension over ten nucleotides, with modified bases introduced at defined positions with highest incorporation efficiency. These results demonstrate the robustness of our proof‐of‐concept for precise incorporation of modified genetic building blocks into DNA. Importantly, no harsh deprotection steps, such as acid detritylation are required, preserving DNA integrity. Our templated synthesis provides intrinsic error correction where misincorporations are minimized and faulty sequences progressively lose template synchrony with each incorporation/deprotection cycle, eliminating the need for additional blocking steps typical of phosphoramidite chemistry. Synthesized DNA fragments can be released from the solid support using endonuclease V to obtain clean oligonucleotides as previously described [55].

Our study provides a flexible and direct method for the site‐specific incorporation of multiple modifications into DNA, enabling precise engineering of nucleic acids for applications including fluorescent and affinity tagging, epigenetic studies, and investigations of nucleic acid structure and interactions. In epigenetic studies, this approach could enable systematic investigation of how defined epigenetic patterns influence gene expression and protein interactions, as well as the generation of DNA libraries carrying precise epigenetic modifications that can serve as reference standards for epigenetic sequencing. In structural biology, site‐specific modifications enable strategies such as fluorescent labelling for FRET‐based analysis of conformational dynamics, or the incorporation of crosslinkers to stabilize transient nucleic acid‐protein complexes for structural characterization by cryo‐EM or X‐ray crystallography. Spin labels can further support EPR measurements to obtain distance constraints within macromolecular assemblies, while tailored base modifications can enhance structural stability or facilitate crystallization of challenging targets. Together, our approach may enable deeper insights into the structure, dynamics, and function of complex biological systems.

Importantly, the underlying (bio)chemistry is compatible with sequencing‐by‐synthesis (SBS) technology, allowing full automation on standard DNA sequencers capable of synthesizing DNA fragments up to 600 nt in length (Illumina MiSeq). In the absence of sequence identification constraints and due to intrinsic error correction, substantially longer DNA fragments may be accessible. Recent studies have demonstrated how DNA sequencers can be repurposed for alternative applications [57, 58, 59, 60, 61], providing a path toward eco‐friendly, high‐fidelity routine synthesis of site‐specifically modified, biologically relevant long DNA fragments far beyond 200 nt to potentially eliminate laborious assembly steps in the future.

## Author Contributions

R.R and B.R. conducted all experiments and analysed data. R.R. performed the chemical synthesis. B.R. cloned, expressed and purified polymerase mutants. All authors interpreted the data and wrote the manuscript. S.B. supervised the study and acquired funding.

## Conflicts of Interest

A patent application related to this work has been filed by the authors.

## Supporting information




**Supporting File**: advs75917‐sup‐0001‐SuppMat.pdf.

## Data Availability

All analysed data are available within the manuscript and the Supplementary Information. The LC–MS raw data are publicly accessible for download via ftp://massive‐ftp.ucsd.edu/v13/MSV000101869/.
