# Supplementary material for: A General Enzymatic Strategy for Site‐Specific Incorporation of Modified Genetic Building Blocks Into DNA
Source: Adv Sci (Weinh). 2026 Jun 2:e75917. Online ahead of print. doi: 10.1002/advs.75917 (PMC13336351; doi:10.1002/advs.75917)

SUPPLEMENTARY INFORMATION

A General Enzymatic Strategy for Site-Specific Incorporation of Modified Genetic Building Blocks into DNA

Raveena Raveena[1,2,#], Bhavana Ramadas[1,2,#], Sidney Becker[1,2,*]

[1] Max-Planck Institute for Molecular Physiology, Otto-Hahn-Str. 11, 44227 Dortmund
[2] Technical University Dortmund, Faculty for Chemistry and Chemical Biology, Otto-Hahn-Str. 4a, 44227 Dortmund

[#] Authors contributed equally
* Correspondence to sidney.becker@mpi-dortmund.mpg.de

Methods

Reagents and Procedures

All the oligonucleotides were commercially purchased as HPLC-purified from Sigma Aldrich and mixed with the advised amount of milli-Q water to obtain 100 µM solution. rt-dNTPs (natural A, T, G and C) were purchased from Jena Bioscience. Therminator polymerase (M0261S), Terminal Transferase (TdT, M3015S) and EndoV (M0305S) were purchased from New England Biolabs. Urea PAGE buffers were purchased from Carl Roth.

All the reagents & starting material were purchased from Sigma Aldrich, Thermo Fisher, TCI and BldPharm, and were used in synthetic procedures without further purification. Anhydrous solvents with Acros seal and molecular sieves were purchased from Thermo Scientific. Silica-coated aluminium plates (Merck 60 F254) were used for thin layer chromatography (TLC) to monitor reactions and were visualized under UV irradiation (254 nm) or by staining with p-anisaldehyde (135mL absolute EtOH, 5mL concentrated H_2SO_4, 1.5mL CH_3COOH, 3.7mL p-anisaldehyde) or $KMnO_4$ (1.5g of $KMnO_4$, 10g K_2CO_3, and 1.25mL 10% NaOH in 200mL water). Analytical LC-MS were performed on Thermo Scientific dionex Ultimate 3000 (uHPLC) connected with Velos Pro ETD (Electron-transfer dissociation) MS using column: ec 50/2 Nucleodur C18 gravity, 1.8 µM (eluents: Acetonitrile (+ 0.1 % TFA) and Water (+ 0.1 % TFA)).

The crude mixture purification was done with silica gel (Merck 60, particle size 0.040-0.063 mm) column chromatography using the suitable eluent system purchased from VWR. Purification of phosphorylated compounds were carried out with Thermo Scientific Vanquish HPLC system using column (i) Macherey Nagel, VP 250/10 Nucleodur 100-5 C18 ec (eluents - A: 100mM TEAA (triethyl ammonium acetate) in water, pH= 7 and B: 100mM TEAA (triethyl ammonium acetate) and (ii) Ion – Exchange column: Thermo Scientific DNAPac™ PA200 Semi-prep 9*250mm (eluents - C: 20mM Tris in water, pH = 8 and D: 1.25M NaCl + 20mM Tris in water, pH = 8).

NMR data were collected with Bruker Avance III HD 400 MHz spectrometer (NanoBay), Bruker Avance III HD 500 MHz spectrometer (Prodigy Cryoprobe), Agilent DD2 500 MHz (triple resonance), Avance III HD 600 MHz

spectrometer (Cryoprobe), Bruker Avance NEO 600 MHz spectrometer (Cryoprobe), Bruker Avance III HD 700 MHz spectrometer (Cryoprobe). NMR was performed with $CDCl_3$, DMSO-d_6 and D_2O solvents, and with reference to the solvent chemical shifts reported in ppm (parts per million), coupling constant in Hz and multiplicity with abbreviations like s (singlet), d (doublet), t (triplet), dd (double doublet), m (multiplet) etc. Positive HRMS data were recorded with an Agilent 1200 system HPLC coupled to a Compact Q-tof mass spectrometer from Bruker with ESI (electro spray ionization) ionization source. Negative HRMS experiments of nucleotides and oligonucleotides were conducted with Thermo Scientific Vanquish HPLC system coupled with an Orbitrap Exploris 120 ESI-MS using Thermo Scientific DNAPac RP 4 μM, 2.1 × 100 mm column (eluents - 50 mM HFIP (1,1,1,3,3,3-hexafluoro-2-propanol), 15 mM TEA (triethylamine), pH 9.00 (solvent A) and methanol (solvent B) at 80 °C. MS data of oligonucleotide experiments were deconvoluted with the help of FreeStyle 1.8.

Expression & purification of polymerases

Plasmid pOpen-9N7polA (CT) (#165502) was purchased from Addgene, which carries the *Thermococcus* sp. (strain 9°N – 7) DNA polymerase gene with the mutations L408S/Y409A/P410V in the catalytic site, in addition to the 3'-5' exonuclease mutations. Site-directed mutagenesis was performed using Gibson assembly to introduce the mutations suggested in the Illumina patent. The two mutant gene variants were cloned into the expression vector pET-16b (Novagen) (which has an N-terminal 10xHis-Tag) via restriction enzyme cloning. YAV & YAS gene constructs were expressed and purified following the Illumina patent protocol with slight changes. BL21(DE3) RIL Codon Plus competent cells (Agilent Technologies) were transformed with the plasmids and single colonies were picked for overnight primary culture growth. Further, LB media was inoculated with 1/100th volume of primary culture and over expression of the polymerase was induced using 1 mM IPTG at OD600 of 0.6. After cell lysis, samples were incubated at 75 °C for 30 minutes before pelleting. This step removes E. coli proteins that are not thermostable, hence simplifying purification. The protein was purified using a HiTrap TALON column (Cytiva), followed by size-exclusion chromatography with HiLoad 26/600 Superdex 200 pg column (Cytiva).

Single nucleotide incorporation of rt-dNTPs

The incorporation experiments were conducted with 20 μL final volume. 0.25 μM 5'- 6FAM labelled Primer (Primer_1, 24mer) and 0.50 μM template (6C_template/6A_template/6T_template/6G_Template, 30mer) were annealed in 2 μL of 10x ThermoPol buffer (NEB, final 1x) by incubating at 95 °C for 5 minutes followed by a gradual decrease to 25 °C at a rate of 0.2 °C/s. This is followed by addition of rt-dNTP (10 μM) and polymerase (1U per 20 μL). In the experiments testing effect of Mn^{2+}, 0.5 mM Mn^{2+} solution was also added before polymerase. The incorporation reactions were performed by incubating sample at 60 °C over time intervals. Quenching of the reaction was achieved by adding equal volume of Gel Loading Dye II (Invitrogen) followed by incubation at 95 °C for 5 minutes. Analysis of the experiment was completed by loading all the samples on 20% Urea-PAGE gel and imaged by ChemiDoc Imaging System (Bio-Rad).

Steady state kinetic analysis

Steady state kinetics experiments were performed following the protocol of O'Flaherty with minor changes. Oligonucleotides were purchased from Sigma-Aldrich. Primer-extension assays were performed (in steady state conditions) in ThermoPol buffer (NEB), with 1 μM template, 1 μM primer, and 10 mM Mg^{2+}. Primer (Primer_1) and template (6C_template/6A_template/6T_template/6G_Template) were annealed before adding polymerase and rt-dNTP by incubating at 95 °C for 5 minutes followed by a gradual decrease to 25 °C at a rate of 0.1 °C/s. Polymerase concentrations ranged from 10 nM - 200 nM, depending on the rt-dNTP, optimized for steady state conditions (less than 20% product formation). All samples were incubated for 5 minutes at 45 °C before adding rt-dNTP, to allow polymerase binding to the primer-template duplex. After adding rt-dNTP, samples were again incubated at 45 °C for 5 minutes for primer extension. Reactions were quenched by adding Gel Loading Dye II (Invitrogen) and incubating at 95 °C for 5 minutes. All assays were performed in duplicates. Samples were loaded on 20% Urea-PAGE gels, and imaged using ChemiDoc Imaging System (Bio-Rad). Gels were analysed using ImageLab software. Band intensities were used to calculate percentage of product formation, and subsequently turnover values. The rt-dNTP concentrations and corresponding turnover values were imported into GraphPad Prism software for Michaelis-Menten analysis.

Iterative incorporation of rt-dNTPs & site-specific incorporation of modified nucleotides on streptavidin beads

The iterative incorporation experiments were performed on Dynabeads™ MyOne™ Streptavidin C1 (Invitrogen). 5' biotinylated Primer_2 (20 pmol) was annealed to Template_1 (30 pmol) by incubating at 95 °C for 5 minutes and gradually decreasing the temperature to 25 °C at the rate of 0.1 °C/s. The annealed oligonucleotides were then bound to the streptavidin beads (8 μL beads for 20pmol oligo) following the manufacturer's instructions. Incorporation reactions were performed in ThermoPol buffer (NEB, 1x), at 60 °C for 30 minutes, with 50 μM rt-dNTP and 0.5U/20 μL YAV polymerase. Deprotection was carried out using Illumina cleavage mix, at 60 °C for 10 minutes. The beads were washed after each step with wash buffer (5 mM Tris HCl pH 7.5, 0.5 mM EDTA, 1 M NaCl). The final deprotection step is skipped to obtain a distinct UV peak for the final product. The oligos were eluted from the beads by incubating in 95% formamide, 10 mM EDTA at 95 °C for 5 minutes. Samples were purified using the Oligo Clean & Concentrator kit (Zymo Research), and run on the Orbitrap Exploris 120 mass spectrometer for analysis. Abundance of primer-extension products was calculated using BioPharma Finder™ 5.1. Efficiency was calculated as the relative abundance of the final primer-extension product in comparison to all the incomplete extension products. All measured MS errors were below 10 ppm. Similarly, the site-specific incorporation of the modified nucleotides 8oxoG, 8oxoA and 5mC were carried out with the templates Template_1, Template_2, Template_3 and Template_hairpin respectively.

Endonuclease cleavage & denaturation of the site-specifically modified oligonucleotide

The 10nt extension of hairpin was achieved by iterative incorporation of rt-dNTP on streptavidin beads. The extended hairpin (20pmol, final reaction volume = 20 μL) on beads was incubated at 37 °C for 1h with/in NEBuffer™ 4 and EndoV (10U/10pmol oligo). Denaturation of the cleaved strand was performed by incubating

at 80 °C for 10min. The supernatant consisting of site-specifically modified oligonucleotide was purified with Oligo Clean & Concentrator kit (Zymo Research), analysed with the Orbitrap Exploris 120 mass spectrometer. MS data analysis was conducted using BioPharma Finder™ 5.1 to calculate efficiency and MS2 fragmentation was performed to confirm the position of the modified nucleotides under the error below 10 ppm.

Incorporation of rt-dNTP using Template independent Polymerase (TdT)

The incorporation experiments were performed on Dynabeads™ MyOne™ Streptavidin C1 using the commercially purchased TdT polymerase. 5' biotinylated Primer_2 (20 pmol) was bound to Streptavidin beads (8 µL) using the manufacturer's protocol. Afterwards the beads were washed and used for incorporation reaction (reaction final volume = 10 µL) in Terminal Transferase reaction buffer (NEB, 1x in reaction) with 200 µM rt-dNTP, 250 µM $CoCl_2$ and 10U/10 µL TdT polymerase at 37 °C for 30 minutes and 1h. The extended oligos were eluted from the beads by incubating in elution buffer (95% formamide, 10 mM EDTA) at 95 °C for 5 minutes. Samples were purified using the Oligo Clean & Concentrator kit (Zymo Research), and run on the Orbitrap Exploris 120 mass spectrometer for analysis. Abundance of primer-extension products was calculated using BioPharma Finder™ 5.1.

Supplementary Figures

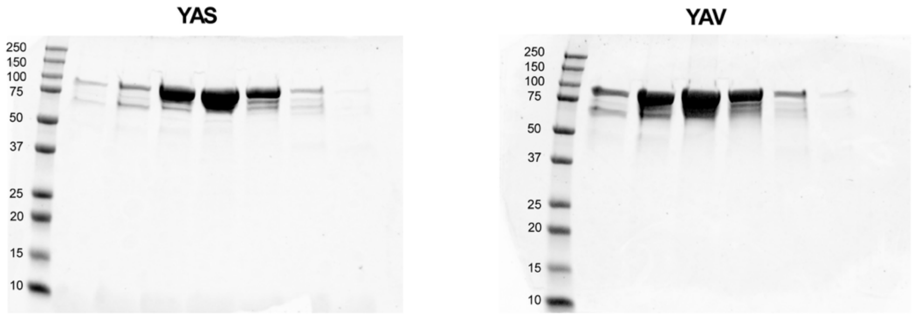

Figure S1: SDS-PAGE gels of purified polymerases YAS and YAV after affinity chromatography with HiTrap TALON (Cytiva) column and size exclusion chromatography with HiLoad 26/600 Superdex 200 pg column (Cytiva). (Molecular weights of polymerases range from 92-93 kDa).

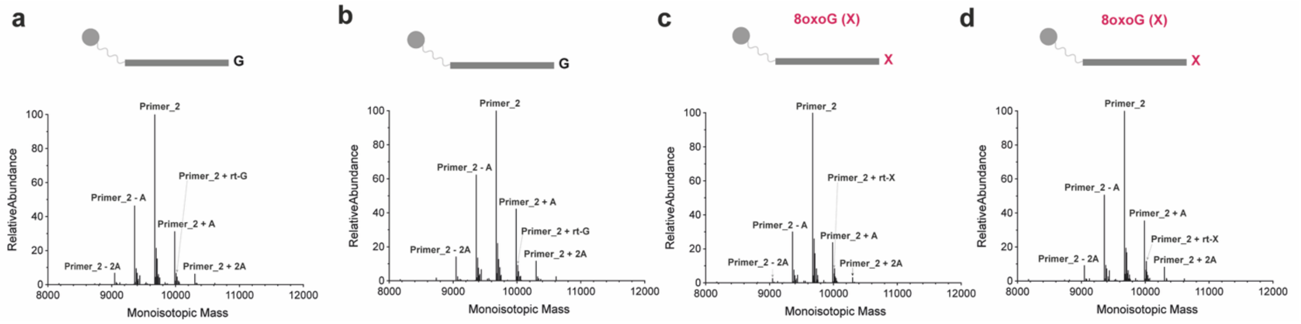

Figure S2: Deconvoluted MS spectra of rt-dNTP incorporation with TdT polymerase **(a)** rt-dG, incubation at 37 °C over 30 min; observed mass = 9048.632 (Primer_2 – 2A), 9361.685 (Primer_2 – A), 9674.741 (Primer_2), 9987.793 (Primer_2 + A), 10058.785 (Primer_2 + rt-dG) and 10300.857 (Primer_2 + 2A) **(b)** rt-dG, incubation at 37 °C over 1 h; observed mass = 9048.626 (Primer_2 – 2A), 9361.680 (Primer_2 – A), 9674.738 (Primer_2), 9987.792 (Primer_2 + A), 10058.801 (Primer_2 + rt-dG) and 10300.849 (Primer_2 + 2A) **(c)** rt-8oxoG, incubation at 37 °C over 30 min; observed mass = 9048.630 (Primer_2 – 2A), 9361.687 (Primer_2 – A), 9674.744 (Primer_2), 9987.798 (Primer_2 + A), 10074.788 (Primer_2 + rt-8oxoG) and 10300.858 (Primer_2 + 2A) **(d)** rt-8oxoG, incubation at 37 °C over 1h; observed mass = 9048.634 (Primer_2 – 2A), 9361.687 (Primer_2 – A), 9674.744 (Primer_2), 9987.802 (Primer_2 + A), 10074.781 (Primer_2 + rt-8oxoG) and 10300.856 (Primer_2 + 2A). Theoretical mass = 9048.610 (Primer_2 – 2A), 9361.668 (Primer_2 – A), 9674.725 (Primer_2), 9987.790 (Primer_2 + A), 10058.795 (Primer_2 + rt-dG),10074.790 (Primer_2 + rt-8oxoG) and 10300.841 (Primer_2 + 2A).

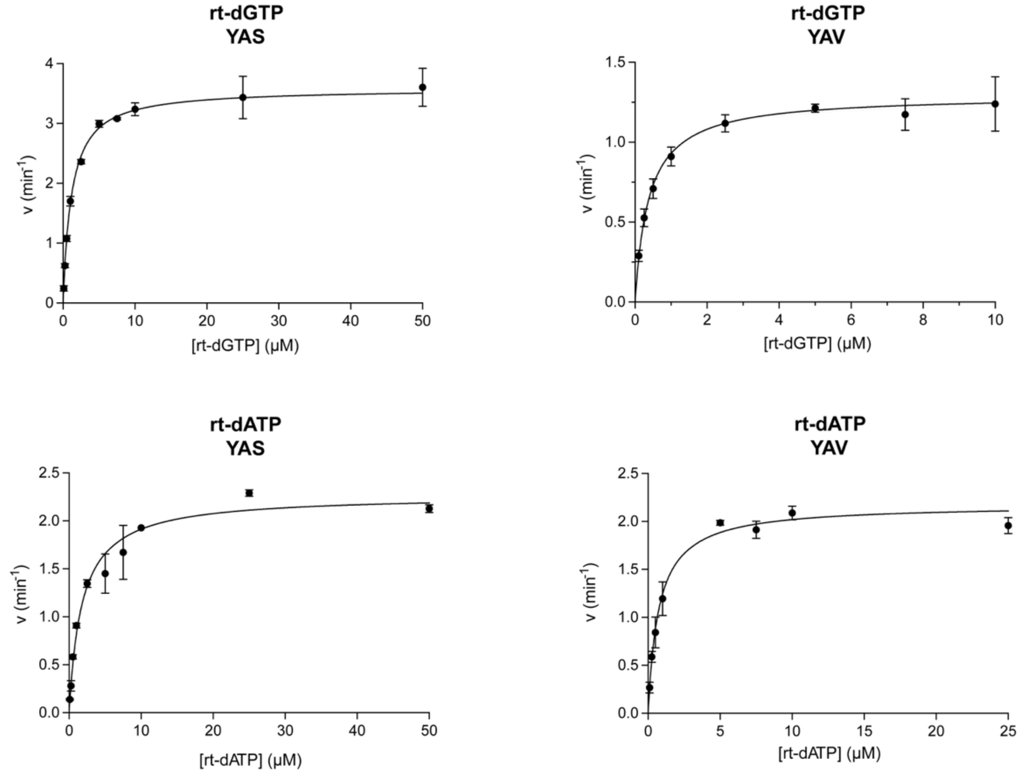

Figure S3: Steady-state kinetics plots of incorporation of rt-dGTP and rt-dATP by YAS and YAV

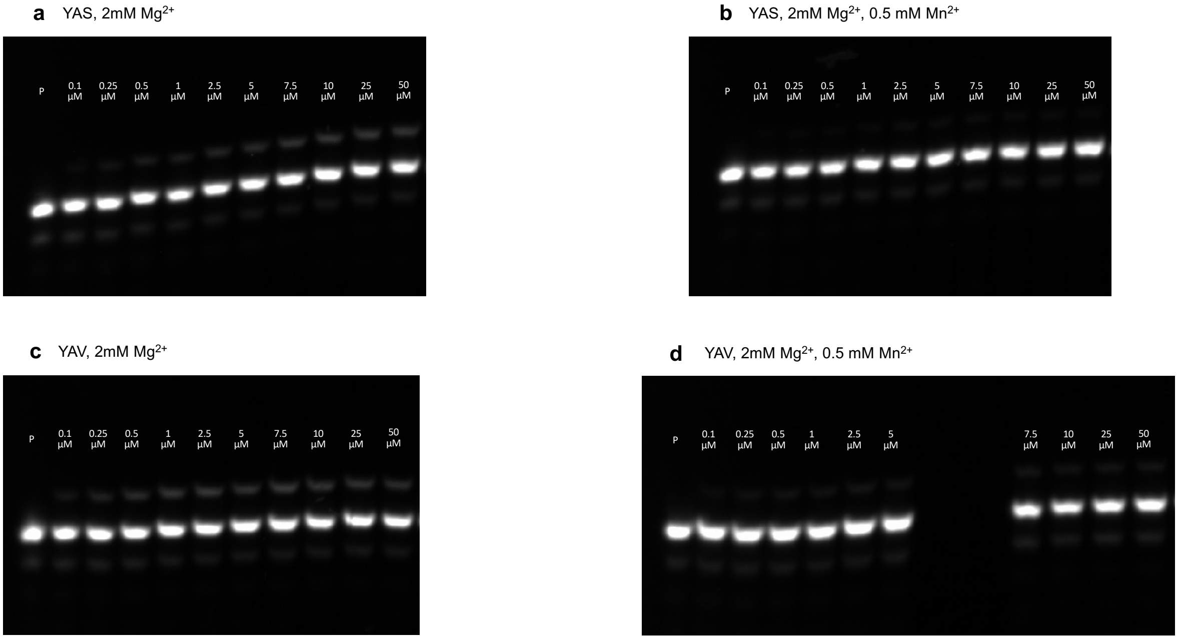

Figure S4: Effect of Mn^{2+} on the incorporation of rt-dGTP (steady-state conditions): adding Mn^{2+} decreases the incorporation of rt-dGTP by both YAS **(a & b)** and YAV **(c & d)**. The reduced incorporation efficiency in the presence of Mn^{2+} prevented accurate determination of kinetic parameters under these conditions.

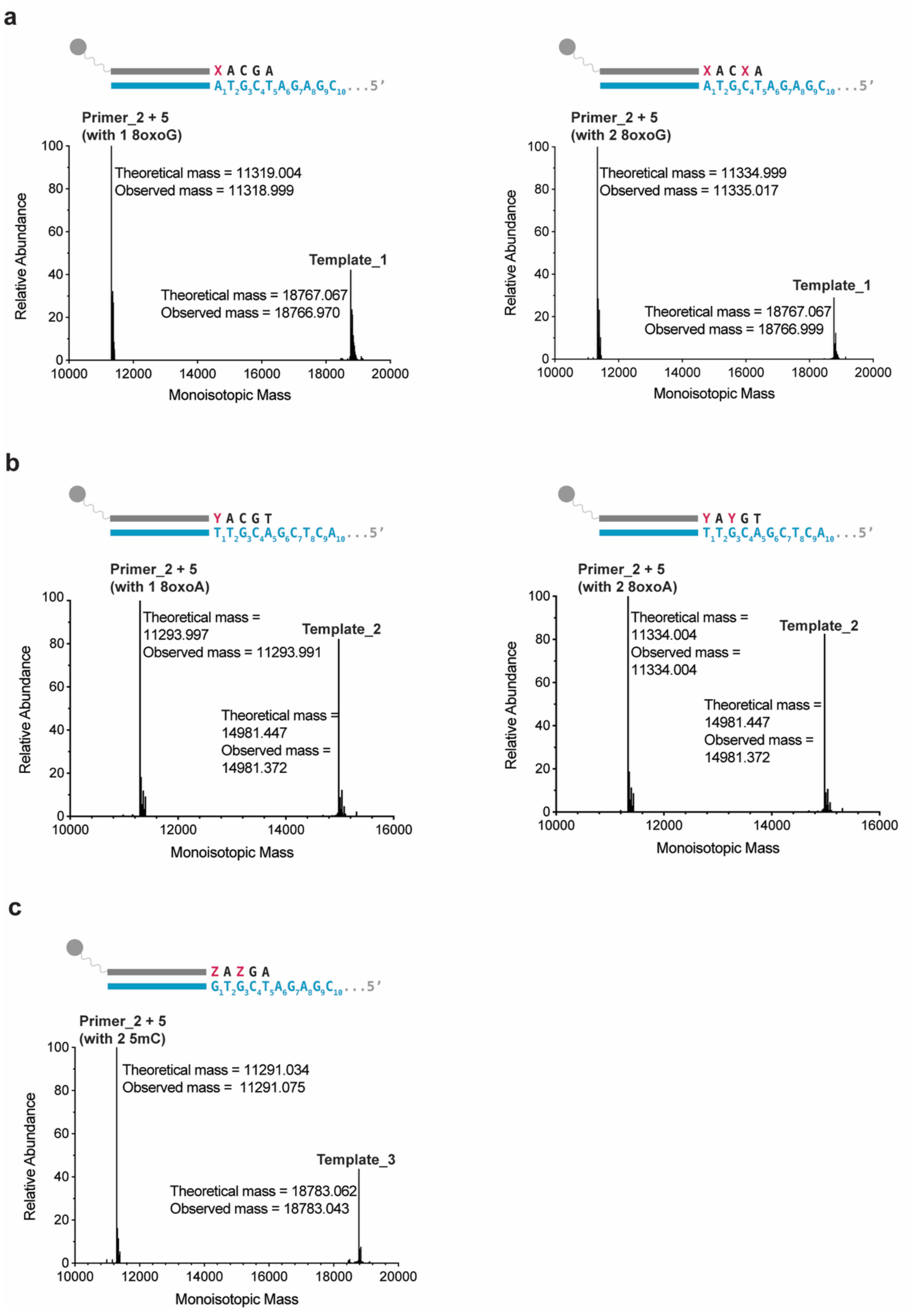

Figure S5: Deconvoluted MS spectra of 5-nucleotide extension of Primer_2 with site-specific incorporation of 8oxoG **(a)**, 8oxoA **(b),** and 5mC **(c)**

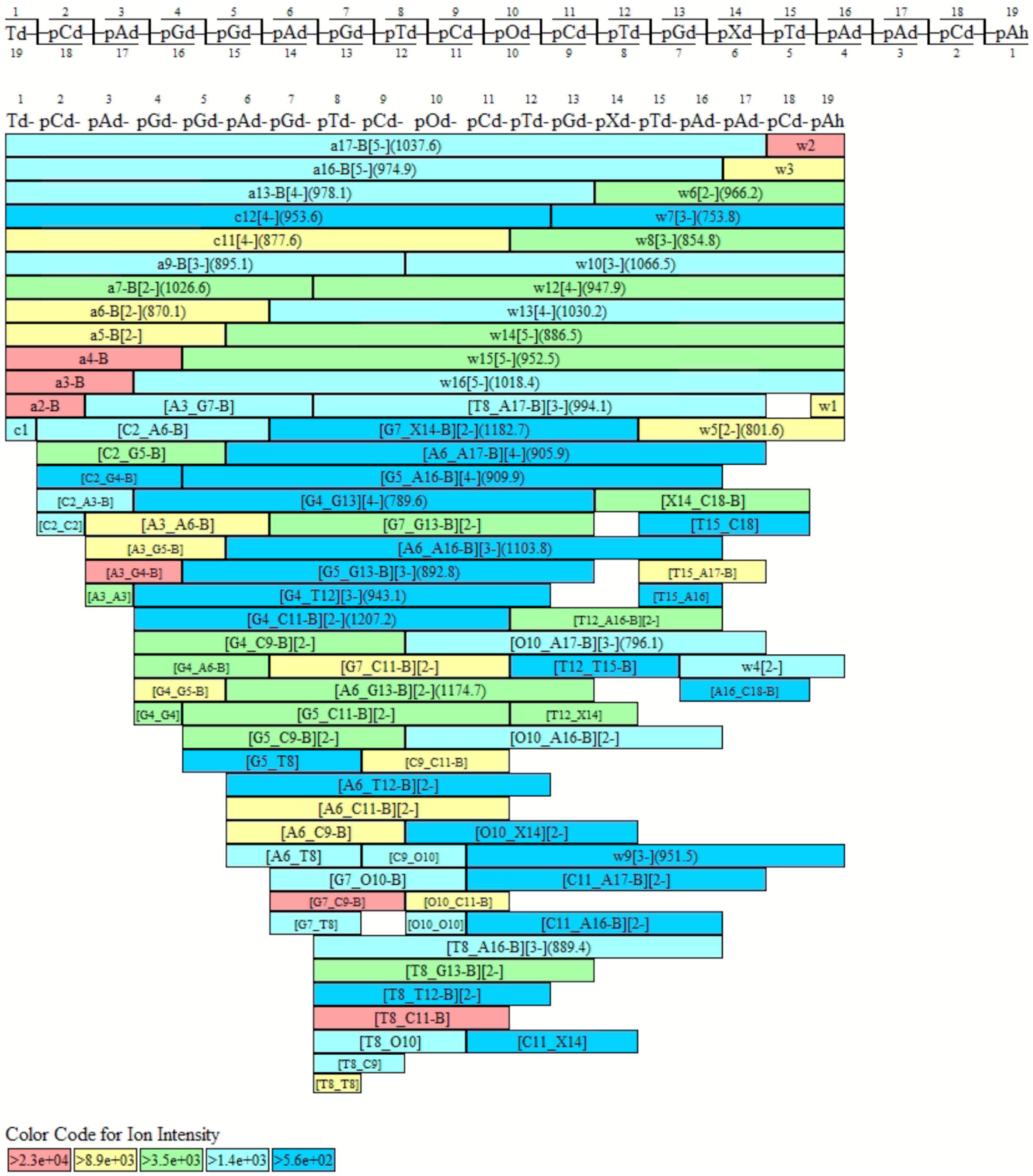

Figure S6: Fragment alignment of the synthesized oligo sequence as analyzed with BioPharma Finder 5.1: MS/MS data confirms DNA sequence with site-specific modifications of 8oxoG (O) and 8oxoA (X). Only data with ASR (average spectral resolution) = 1, confidence = 100 and error within 10ppm is considered for analysis.

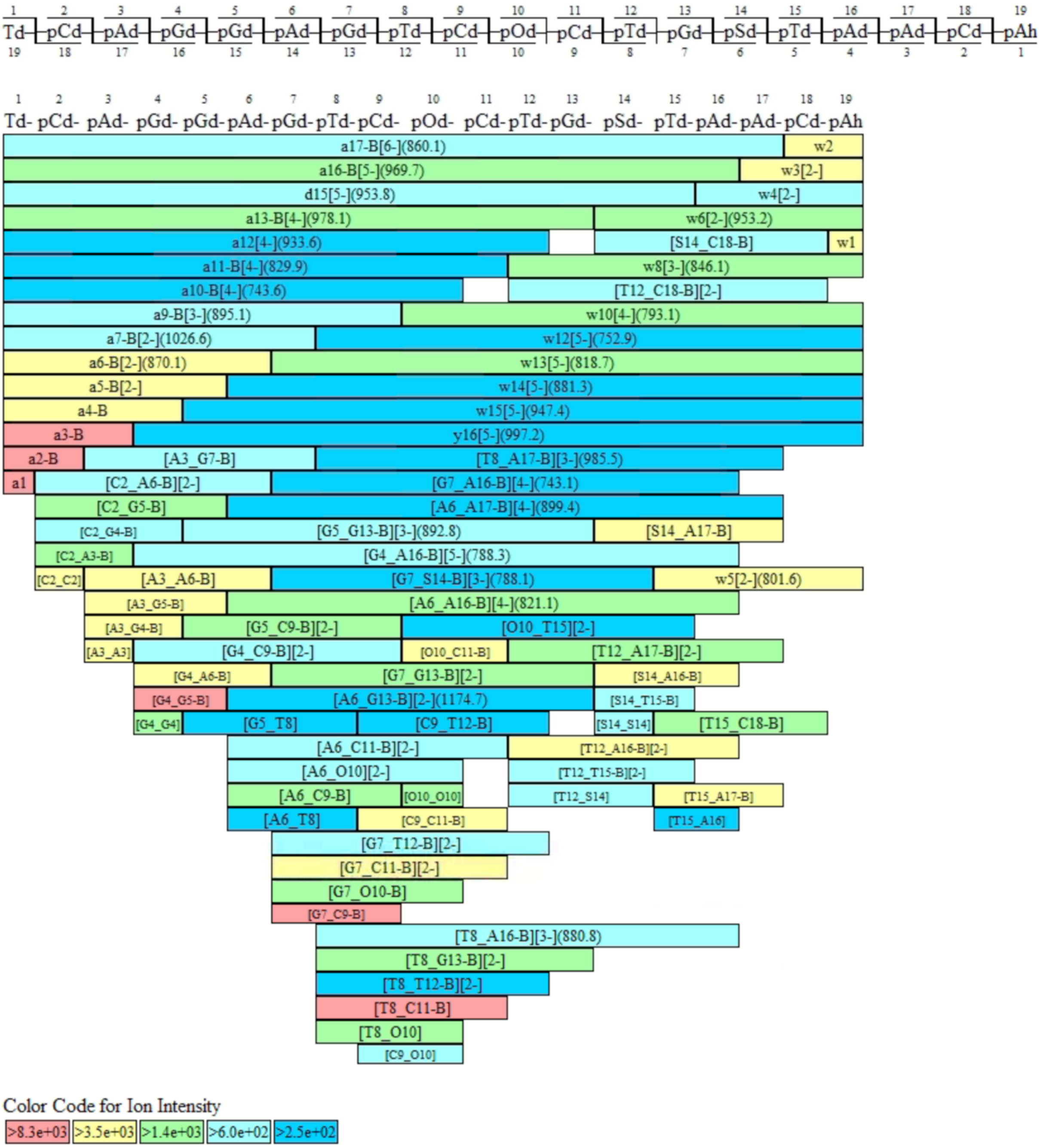

Figure S7: **Fragment alignment of the synthesized oligo sequence as analyzed with BioPharma Finder 5.1:** MS/MS data confirms DNA sequence with site-specific modifications of 8oxoG (O) and 5mC (S). Only data with ASR (average spectral resolution) = 1, confidence = 100 and error within 10ppm is considered for analysis.

Name	Sequence (5' - 3')
Primer_1	[6FAM] GTTTTGGCTACCTGTTACTAAGCA
Primer_2	[Biotin] CGGGCGGACCAGAACCCTTGAGCACAGAAA
6C_Template	CCCCCCTGCTTAGTAACAGGTAGCCAAAAC
6A_Template	AAAAAATGCTTAGTAACAGGTAGCCAAAAC
6T_Template	TTTTTTTGCTTAGTAACAGGTAGCCAAAAC
6G_Template	GGGGGGTGCTTAGTAACAGGTAGCCAAAAC
Template_1	CTTCGTCGGTGACTAACTGTGCGAGATCGTATTTCTGTGCTCAAGGGTTCTGGTCCGCCCG
Template_2	CTTCGGCTAACTCGACGTTTTTCTGTGCTCAAGGGTTCTGGTCCGCCCG
Template_3	CTTCGTCGGTGACTAACTGTGCGAGATCGTGTTTCTGTGCTCAAGGGTTCTGGTCCGCCCG
Template_hairpin	TGTTAGCAGCGACTCCTGAGCGTCGAT[BtndT]CAACGAC**I**CTCAGGAGTC

Table S1: Sequences of oligonucleotides (purchased from Sigma-Aldrich) used in experiments.

Oligos detected	Yield (%)
Primer_2 + TA(rt-C)	96.37
Primer_2 + TA	1.86
Primer_2 + T(rt-A)	0.14
Primer_2 + T	1.15
Primer_2	0.48

Table S2: Primer_2 + 3 rt-dNTP incorporation results with 0.1U/20μL YAV (60 °C, 30 min)

Oligos detected	Yield (%)
Primer_2 + TACG(rt-A)	98.72
Primer_2 + TACG	0.20
Primer_2 + TAC(rt-G)	0.41
Primer_2 + TAC	0.13
Primer_2 + TA	0.10
Primer_2 + T	0.13
Primer_2	0.32

Table S3: Primer_2 + 5 rt-dNTP incorporation results with 0.5U/20μL YAV (60 °C, 30 min)

Oligos detected	Yield (%)
Primer_2 + XACG(rt-A)	99.57
Primer_2 + XACG	0.20
Primer_2 + XAC(rt-G)	0.06
Primer_2 + XAC	0.12
Primer_2 + XA	0.03
Primer_2 + X	0.03

Table S4: Primer_2 + 5 rt-dNTP (with one 8oxoG (X)) incorporation results with 0.5U/20µL YAV (60 °C, 30 min)

Oligos detected	Yield (%)
Primer_2 + XACX(rt-A)	98.54
Primer_2 + XACX	0.96
Primer_2 + XAC	0.36
Primer_2 + XA	0.02
Primer_2 + X(rt-A)	0.07
Primer_2 + X	0.05

Table S5: Primer_2 + 5 rt-dNTP (with two 8oxoG (X)) incorporation results with 0.5U/20µL YAV (60 °C, 30 min)

Oligos detected	Yield (%)
Primer_2 + YACG(rt-T)	99.83
Primer_2 + YACG	0.14
Primer_2 + YAC	0.03

Table S6: Primer_2 + 5 rt-dNTP (with one 8oxoA (Y)) incorporation results with 0.5U/20uL YAV (60 °C, 30 min)

Oligos detected	Yield (%)
Primer_2 + YAYG(rt-T)	99.92
Primer_2 + YAYG	0.05
Primer_2 + YAY	0.03

Table S7: Primer_2 + 5 rt-dNTP (with two 8oxoA (Y)) incorporation results with 0.5U/20uL YAV (60 °C, 30 min)

Oligos detected	Yield (%)
Primer_2 + ZAZG(rt-A)	98.78
Primer_2 + ZAZG	0.41
Primer_2 + ZAZ	0.32
Primer_2 + ZA	0.14
Primer_2 + Z	0.25
Primer_2	0.11

Table S8: Primer_2 + 5 rt-dNTP (with two 5mC (Z)) incorporation results with 0.5U/20µL YAV (60 °C, 30 min)

Oligos detected	Yield (%)
Primer_2 + TACXATCXC(rt-G)	99.31
Primer_2 + TACXATCXC	0.02
Primer_2 + TACXATCX	0.30
Primer_2 + TACX	0.06
Primer_2 + TAC	0.04
Primer_2 + TA	0.09
Primer_2 + T	0.17

Table S9: Primer_2 + 10 rt-dNTP (with two 8oxoG (X)) incorporation results with 0.5U/20μL YAV (60 °C, 30 min)

Oligos detected	Yield (%)
Primer_2 + AYCGTYGAG(rt-T)	99.65
Primer_2 + AYCGT	0.13
Primer_2 + A	0.22

Table S10: Primer_2 + 10 rt-dNTP (with two 8oxoA (Y)) incorporation results with 0.5U/20μL YAV (60 °C, 30 min)

Oligos detected	Yield (%)
Primer_2 + CAZGATZTC(rt-G)	99.64
Primer_2 + CAZGATZT	0.02
Primer_2 + CAZGATZ	0.02
Primer_2 + CAZGAT	0.02
Primer_2 + CAZGA	0.02
Primer_2 + CAZG	0.08
Primer_2 + CAZ	0.04
Primer_2 + C	0.08

Table S11: Primer_2 + 10 rt-dNTP (with two 5mC (Z)) incorporation results with 0.5U/20μL YAV (60 °C, 30 min)

Oligos detected	Yield (%)
HP_fragment + XCTGYTAAC(rt-A)	99.8
HP_fragment + XCTGYTAAC	0.11
HP_fragment + XCTGYTAA	0.02
HP_fragment + XCTGYTA	0.11

Table S12: HP_fragment (Hairpin_fragment = TCAGGAGTC) + 10 rt-dNTP (with 8oxoG (X) at position 1st and 8OA (Y) at position 5th) incorporation results with 0.5U/20μL YAV (60 °C, 30 min)

Oligos detected	Yield (%)
HP_fragment + XCTGZTAAC(rt-A)	99.52
HP_fragment + XCTGZTAA(rt-C)	0.08
HP_fragment + XCTGZTA(rt-A)	0.20
HP_fragment + XCTGZT(rt-A)	0.08

Table S13: HP_fragment (Hairpin_fragment = TCAGGAGTC)+ 10 rt-dNTP (with 8oxoG (X) at position 1st and 5mC (Z) at position 5th) incorporation results with 0.5U/20µL YAV (60 °C, 30 min)

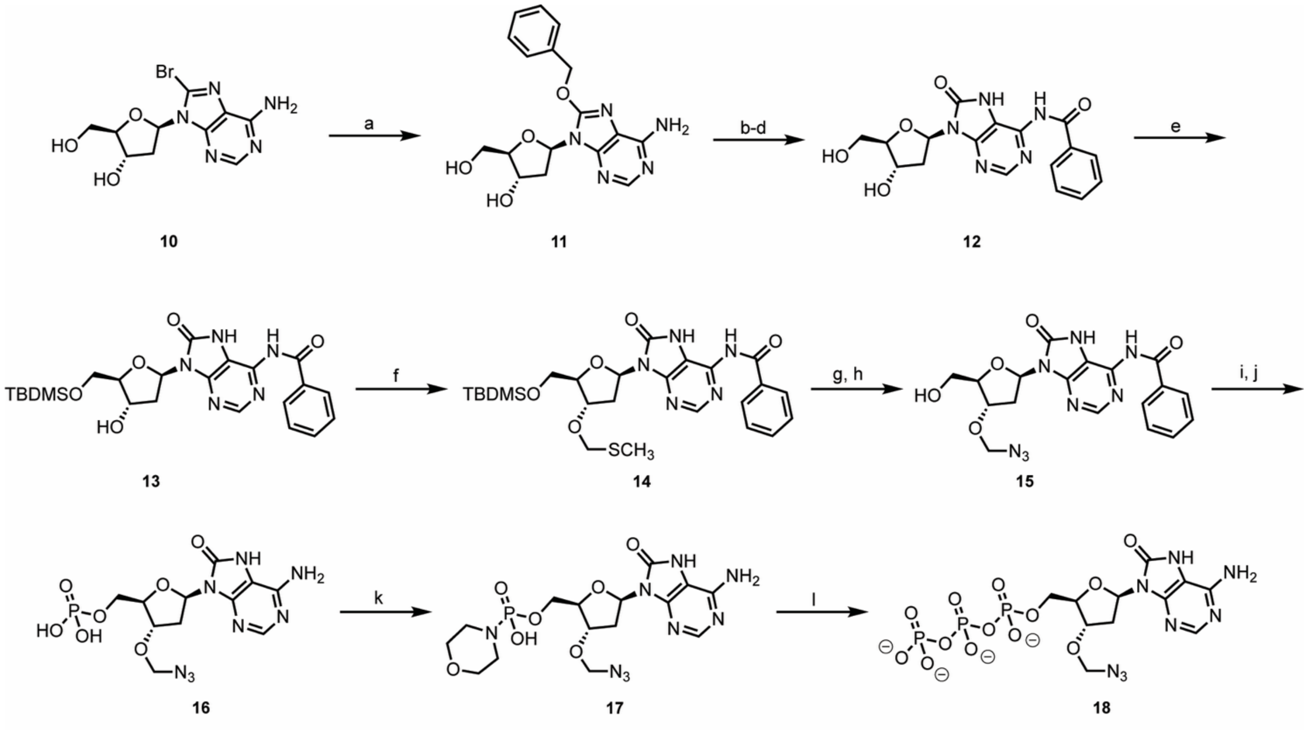

Scheme S1: Synthesis of 3'-O-azidomethyl-8-oxo-dATP 18 from 8-bromo-dA 10.
(a) NaH, BnOH , room temperature, 18h, 91%, (b) Benzoyl chloride, Pyridine, room temperature, 3h (c) 2N NaOH, pyridine-dioxane(1:1), room temperature, 4h (d) 1N HCl, MeOH, room temperature, 1h, 82% over 3 steps, (e) TBDMSCl, Pyridine, room temperature, 2h, 88%, (f) DMSO, AcOH, Ac_2O, room temperature, 46.5h, 66%, (g) $TMSN_3$, NIS, TfOH, DCM, -40 ºC, 2h (h) NH_4F, MeOH, room temperature, 20h, 37% over 2 steps, (i) $POCl_3$, $PO(OMe)_3$, Proton-sponge, 0 ºC, 2h (j) 7N NH_3 in MeOH, 60 ºC, 20h, 55% over 2 steps, (k) DCC, Morpholine, t-BuOH:H_2O(1:1), 95 ºC, 5h, 91%, (l) $(Bu_3NH)_4P_2O_7$, Tetrazole, DMF, 37 ºC, 15h, 63%.

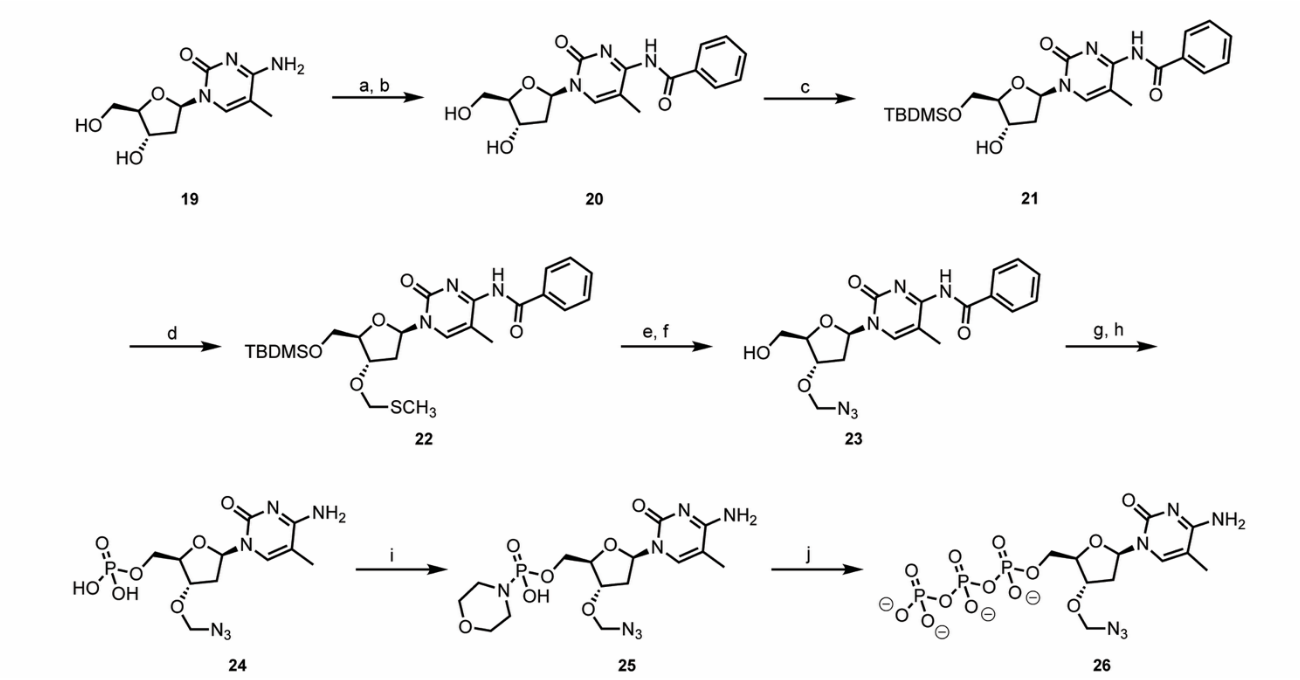

Scheme S2: Synthesis of 3'-O-azidomethyl-5mdCTP 26 from 5mdC 19.

(a) Benzoyl chloride, Pyridine, room temperature, 3h (b) 2N NaOH, pyridine-ethanol(1:1), room temperature, 2h, 62% over 2 steps (c) TBDMSCl, Pyridine, room temperature, 3.5h, 82%, (d) DMSO, AcOH, Ac_2O, room temperature, 49h, 80%, (e) $TMSN_3$, NIS, TfOH, DCM, -20 ºC, 2h (f) NH_4F, MeOH, room temperature, 65.5h, 19% over 2 steps, (g) $POCl_3$, $PO(OMe)_3$, Proton-sponge, 0 ºC, 2h (h) 7N NH_3 in MeOH, 60 ºC, 38.5h, 39% over 2 steps, (i) DCC, Morpholine, t-BuOH: H_2O(1:1), 95 ºC, 5.5h, (j) $(Bu_3NH)_4P_2O_7$, Tetrazole, DMF, 37 ºC, 18h, 46% over 2 steps.

Synthesis of 3’-O-azidomethyl-8-oxo-deoxyguanosine triphosphate

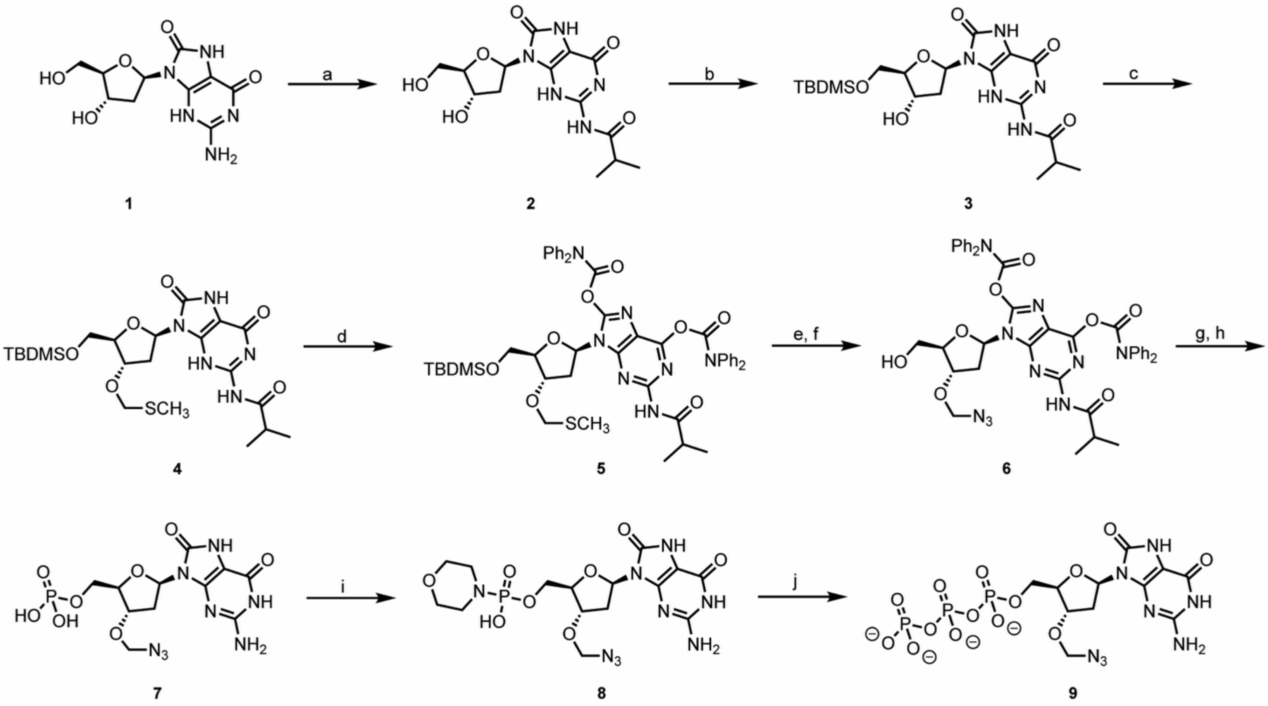

Compound 2:

N-(9-((2*R*,4*S*,5*R*)-4-hydroxy-5-(hydroxymethyl)tetrahydrofuran-2-yl)-6,8-dioxo-6,7,8,9-tetrahydro-3*H*-purin-2-yl)isobutyramide

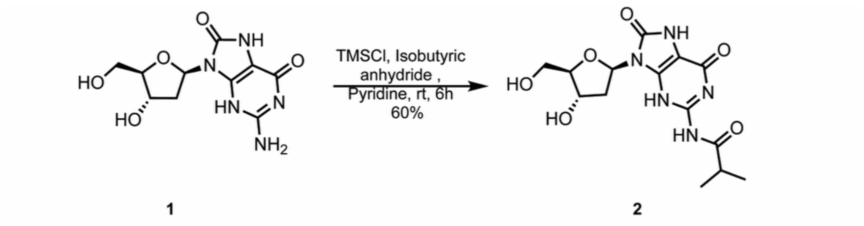

8-oxo-2'-Deoxyguanosine **1** (750 mg, 2.65 mmol) was co-evaporated under reduced pressure with 14.2 mL of pyridine three times and dried over high vacuum overnight. In an inert flask under argon atmosphere dried 8-oxo-2'-Deoxyguanosine (725 mg, 2.56 mmol, 1.00 equiv.) was dissolved in 13.74 mL dry pyridine. To this trimethylchlorosilane (1.62 mL, 12.80 mmol, 5.00 equiv.) was added dropwise. The solution was allowed to stir at RT over 2 h. Isobutyric anhydride (2.12 mL, 12.80 mmol, 5.00 equiv.) was added and the resulted mixture was stirred at RT over 4 h. The reaction mixture was cooled in an ice bath, and 4 mL milli-Q water was added. After 15 min under same conditions 5.26 mL of 25% aqueous ammonia was added and reaction was further allowed to stir over 15 min. The solvent was evaporated under reduced pressure and the obtained residue suspended in 50 mL milli-Q water. The solution was washed with 50 mL DCM. The crude product was obtained by evaporating the aqueous layer under reduced pressure. The crude product was purified with silica gel column

chromatography using MeOH and DCM as eluent system (0% to 12% MeOH/DCM). The protected product **2** was afforded as white powder (540.9 mg, 60% isolated yield).

1H NMR (400 MHz, DMSO-d_6): 12.12 (s, 1H, NH), 11.56 (s, 1H, NH), 11.25 (s, 1H), 6.08 (dd, *J* = 8.0, 6.7 Hz, 1H, 1'-H), 5.15 (d, *J* = 4.0 Hz, 1H, 3'-OH), 4.71 (dd, *J* = 6.5, 5.2 Hz, 1H, 5'-OH), 4.36 (dq, *J* = 6.4, 3.2 Hz, 1H, 3'-H), 3.74 (td, *J* = 5.5, 2.8 Hz, 1H, 4'-H), 3.56 (dt, *J* = 11.0, 5.4 Hz, 1H, 5'-H), 3.43 (dt, *J* = 11.8, 6.0 Hz, 1H, 5'-H), 3.06 (ddd, *J* = 12.9, 8.1, 6.0 Hz, 1H, 2'-H), 2.74 (h, *J* = 6.8 Hz, 1H, 2'-H), 1.97 (ddd, *J* = 12.9, 6.7, 3.0 Hz, 1H, isobutyryl, CH), 1.11 (dd, *J* = 6.8, 1.2 Hz, 6H, isobutyryl, CH_3).
13C NMR (101 MHz, DMSO): δ (ppm): 180.02 (isobutyryl, CO), 151.57 (C8), 149.17(C2 or C6), 147.22 (C2 or C6), 144.89 (C4), 103.37 (C5), 87.33 (C4'), 81.26 (C1'), 71.19 (C3'), 62.26 (C5'), 35.22 (C2'), 34.73 (isobutyryl, CH), 18.88 (isobutyryl, CH_3).
HR-MS (ESI): calcd for $(C14H19N5NaO6)^+$ $[M+Na]^+$: 376.1233, found: 376.1219.

Compound 3:
N-(9-((2*R*,4*S*,5*R*)-5-(((*tert*-butyldimethylsilyl)oxy)methyl)-4-hydroxytetrahydrofuran-2-yl)-6,8-dioxo-6,7,8,9-tetrahydro-3*H*-purin-2-yl)isobutyramide

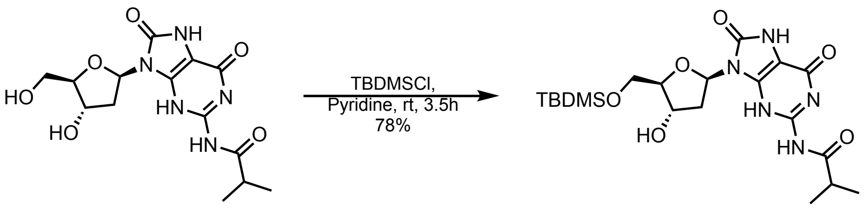

2 3

In an inert Schlenk flask under argon atmosphere compound **2** (599.70 mg, 1.70 mmol, 1.00 equiv.) was dissolved in 3.84 mL dry pyridine. TBDMSCl (281.39 mg, 1.87 mmol, 1.10 equiv.) was added and reaction stirred at RT. After 2.5 h again TBDMSCl (127.91 mg, 0.849 mmol ,0.50 equiv.) was added and the reaction mixture was stirred at RT over 1 h. The reaction was quenched by adding 1ml MeOH. The solvent was removed under reduced pressure. The residue was dissolved in 50 mL ethyl acetate and washed with 50 mL of sat. $NaHCO_3$, water, brine. The organic layer was dried over $MgSO_4$, filtered and evaporated under reduced pressure. The resulted off white crude product was purified with silica gel column chromatography using MeOH and DCM as eluent system (0% to 5% MeOH/DCM). The protected product **3** was afforded as white powder (617.60 mg, 78% isolated yield).

1H NMR (500 MHz, DMSO-d_6): δ (ppm): 12.11 (s, 1H, NH), 11.56 (s, 1H, NH), 11.20 (s, 1H, NH), 6.06 (t, *J* = 7.1 Hz, 1H, 1'-H), 5.17 (d, *J* = 4.2 Hz, 1H, 3'-OH), 4.37 (dq, *J* = 7.0, 3.8 Hz, 1H, 4'-H), 3.78 – 3.66 (m, 2H, 3'-H & 5'-H), 3.67 – 3.57 (m, 1H, 5'-H), 3.11 (dt, *J* = 13.2, 6.6 Hz, 1H, 2'-H), 2.74 (hept, *J* = 6.8 Hz, 1H, isobutyryl, CH), 2.01 (ddd, *J* = 13.0, 7.1, 3.7 Hz, 1H, 2'-H), 1.11 (dd, *J* = 6.9, 1.3 Hz, 6H, isobutyryl, CH_3), 0.82 (s, 9H, 5'-O-$Si(CH_3)_2C(CH_3)_3$), -0.02 (s, 6H, 5'-O-$Si(CH_3)_2C(CH_3)_3$).
13C NMR (101 MHz, DMSO): δ (ppm): 179.93 (isobutyryl, CO), 151.42 (C8), 149.08 (C2 or C6), 147.06 (C2 or C6), 144.89 (C4), 103.39 (C5), 86.80 (C4'), 81.07 (C1'), 70.79 (C3'), 63.78 (C5'), 34.82 (C2'), 34.70 (isobutyryl,

CH), 25.81 (5'-O-Si(CH_3)$_2$C(CH_3)$_3$, CH_3), 18.93 (isobutyryl, CH_3), 18.75 (isobutyryl, CH_3), 18.03 (5'-O-Si(CH_3)$_2$C(CH_3)$_3$, C), -5.29 (5'-O-Si(CH_3)$_2$C(CH_3)$_3$, CH_3), -5.38 (5'-O-Si(CH_3)$_2$C(CH_3)$_3$, CH_3).

HR-MS (ESI): calcd for (C20H33N5NaO6Si)$^+$ [M+Na]$^+$: 490.2098, found: 490.2080.

Compound 4:

N-(9-((2*R*,4*S*,5*R*)-5-(((*tert*-butyldimethylsilyl)oxy)methyl)-4-((methylthio)methoxy)tetrahydrofuran-2-yl)-6,8-dioxo-6,7,8,9-tetrahydro-3*H*-purin-2-yl)isobutyramide

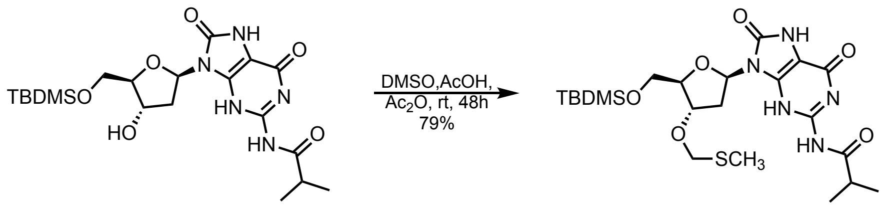

3 4

In an inert Schlenk flask under argon atmosphere compound **3** (486 mg, 1.04 mmol, 1.00 equiv.) was dissolved in 2 mL dry DMSO, 0.95 mL acetic acid and 3.0 mL acetic anhydride were added to the solution. The reaction was allowed to stir at RT over 46 h. Quenching of the reaction was performed by adding 50 mL saturated $NaHCO_3$ solution. The aqueous layer was washed four times with ethyl acetate and the combined organic layer was washed with saturated $NaHCO_3$ solution. The organic layer was dried over Na_2SO_4, filtered and evaporated under reduced pressure to afford crude product. Silica gel column chromatography using MeOH and DCM as eluent system (1:60 to 1: 40, MeOH : DCM) yielded product **4** as white foamy solid (434 mg, 79% isolated yield).

1H NMR (600 MHz, CDCl$_3$): δ (ppm): 12.20 (s, 1H, NH), 10.14 (s, 1H, NH), 9.20 (S, 1H, NH), 6.06 - 6.00 (m, 1H, 1'-H), 4.78 (dt, *J* = 6.7, 4.1 Hz, 1H, 3'-H), 4.68 (d, *J* = 11.4, 1H, 3'-O-CH_2SCH_3), 4.62 (d, *J* = 11.4, 1H, 3'-O-CH_2SCH_3), 3.92 (ddd, *J* = 6.9, 5.2, 3.8 Hz, 1H, 4'-H), 3.73 (qd, *J* = 10.7, 6.1 Hz, 2H, 5'-H), 3.26 (dt, *J* = 13.2, 6.4 Hz, 1H, 2'-H), 2.68 (h, *J* = 7.0 Hz, 1H, isobutyryl, CH), 2.15 (m & s, 4H, 2'-H and 3'-O-CH_2SCH_3), 1.3 (d, *J* = 6.9 Hz, 3H, isobutyryl, CH_3), 1.27 (d, *J* = 6.9 Hz, 3H, isobutyryl, CH_3), 0.85 (s, 9H, 5'-O-Si(CH_3)$_2$C(CH_3)$_3$), 0.03 (s, 3H, 5'-O-Si(CH_3)$_2$C(CH_3)$_3$), 0.02 (s, 3H, 5'-O-Si(CH_3)$_2$C(CH_3)$_3$).

13C NMR (151 MHz, CDCl$_3$): δ (ppm): 178.95 (isobutyryl, CO), 151.94 (C8), 150.13 (C2 or C6), 146.89 (C2 or C6), 145.52 (C4), 104.23 (C5), 84.76 (C4'), 81.87 (C1'), 76.88 (C3'), 73.62 (3'-O-CH_2SCH_3, CH_2), 63.25 (C5'), 50.87(DCM), 36.53 (isobutyryl, CH), 33.14 (C2'), 26.00 (5'-O-Si(CH_3)$_2$C(CH_3)$_3$, CH_3), 19.15 (isobutyryl, CH_3), 18.99 (isobutyryl, CH_3), 18.41 (5'-O-Si(CH_3)$_2$C(CH_3)$_3$, C), 13.93 (3'-O-CH_2SCH_3, CH_3), -5.24 (5'-O-Si(CH_3)$_2$C(CH_3)$_3$, CH_3).

HR-MS (ESI): calcd for (C22H37N5NaO6SSi)$^+$ [M+Na]$^+$: 550.2132, found: 550.2119.

Compound 5:

9-((2*R*,4*S*,5*R*)-5-(((*tert*-butyldimethylsilyl)oxy)methyl)-4-((methylthio)methoxy)tetrahydrofuran-2-yl)-2-isobutyramido-9*H*-purine-6,8-diyl bis(diphenylcarbamate)

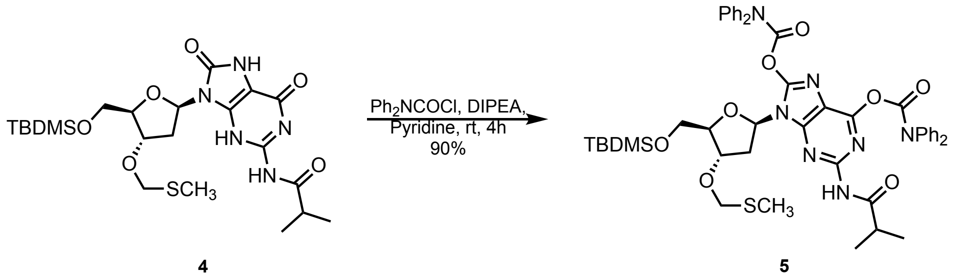

A stirring solution of compound **4** (181.5 mg, 0.344 mmol, 1.00 equiv.) in 3.8 mL dry pyridine under inert atmosphere with argon was treated with diphenylcarbamoyal chloride (239.05 mg, 1.03 mmol, 3.00 equiv.) and DIPEA (176.73 µL, 1.01 mmol, 2.95 equiv.). The reaction was allowed to stir at RT over 4 h. The solvent was removed under reduced pressure and crude product was purified with silica gel column chromatography using Et_2O and petroleum ether as eluent system (25% to 50% Et2O/petroleum ether). The product **5** was afforded as white foamy solid (284.5 mg, 90% isolated yield).

1H NMR (600 MHz, DMSO-*d*6): δ (ppm): 10.75 (s, 1H, NH), 7.48 – 7.12 (m, 20H, diphenylcarbonyl, C_6H_5), 5.87 (t, *J* = 7.0 Hz, 1H, 1'-H), 4.73 – 4.65 (m, 2H, 3'-O-CH_2SCH_3, CH_2), 4.63 (dt, *J* = 6.6, 3.4 Hz, 1H, 3'-H), 3.79 (td, *J* = 6.4, 3.1 Hz, 1H, 4'-H), 3.71 – 3.43 (m, 2H, 5'-H), 3.13 (dt, *J* = 14.0, 6.9 Hz, 1H, 2'-H), 2.67 (h, *J* = 6.8 Hz, 1H, isobutyryl, CH), 2.15 – 2.09 (m, 1H, 2'-H), 2.07 (s, 3H, m, 2H, 3'-O-CH_2SCH_3, CH_3), 1.06 (dd, *J* = 8.5, 6.8 Hz, 6H, isobutyryl, CH_3), 0.80 (s, 9H, 5'-O-$Si(CH_3)_2C(CH_3)_3$), -0.06 (s, 6H, 5'-O-$Si(CH_3)_2C(CH_3)_3$).
13C NMR (151 MHz, DMSO-*d*6): δ (ppm): 174.57 (isobutyryl, CO), 152.21 (C2), 152.03 (C8), 149.86 (C6 or diphenylcarbonyl, CO), 148.47 (C6 or diphenylcarbonyl, CO), 147.38 (C4), 145.77 (C6 or diphenylcarbonyl, CO), 142.12 (diphenylcarbonyl, C_6H_5), 129.47 (diphenylcarbonyl, C_6H_5), 129.27 (diphenylcarbonyl, C_6H_5), 129.17 (diphenylcarbonyl, C_6H_5), 127.85 (diphenylcarbonyl, C_6H_5), 127.11 (diphenylcarbonyl, C_6H_5), 126.38 (diphenylcarbonyl, C_6H_5), 105.98 (C5), 84.51 (C4'), 81.93 (C1'), 76.77 (C4'), 72.83 (3'-O-CH_2SCH_3, CH_2), 62.92 (C5'), 34.72 (isobutyryl, CH), 31.86 (C2'), 25.72 (5'-O-$Si(CH_3)_2C(CH_3)_3$, CH_3), 19.25 (isobutyryl, CH_3), 19.02 (isobutyryl, CH_3), 17.86 (5'-O-$Si(CH_3)_2C(CH_3)_3$, C), 13.26 (3'-O-CH_2SCH_3, CH_3), -5.43 (5'-O-$Si(CH_3)_2C(CH_3)_3$, CH_3), -5.51 (5'-O-$Si(CH_3)_2C(CH_3)_3$, CH_3).
HR-MS (ESI): calcd for $(C48H55N7NaO8SSi)^+$ $[M+Na]^+$: 940.35, found: 940.3493.

Compound 6:
9-((2*R*,4*S*,5*R*)-4-(azidomethoxy)-5-(hydroxymethyl)tetrahydrofuran-2-yl)-2-isobutyramido-9*H*-purine-6,8-diyl bis(diphenylcarbamate)

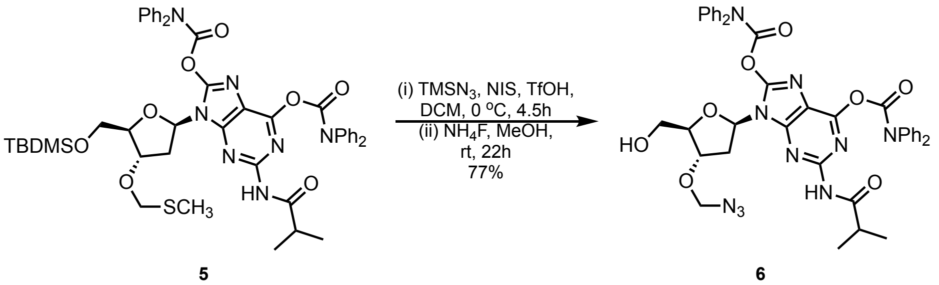

In an inert Schlenk flask under argon atmosphere compound **5** (250 mg, 0.272 mmol, 1.00 equiv.) was dissolved in dry 2.72 mL DCM. $TMSN_3$ (108 µL, 0.817 mmol, 3.00 equiv.) was added and stirred at RT over 10 min. The

reaction mixture was cooled to 0 ºC, NIS (122.52 mg, 0.545 mmol, 2.00 equiv.) and TfOH (28.8 µL, 0.327 mmol, 1.20 equiv.) were added. The reaction mixture was allowed to stir under same conditions over 4.5 h. Quenching of the reaction was performed by adding 5 mL saturated $NaHCO_3$ solution at 0 ºC, filtered, and the filtrate was mixed with 40 mL DCM washed with saturated $Na_2S_2O_3$ and brine. The organic layer was dried over $MgSO_4$, filtered and evaporated under reduced pressure to obtain pale yellow residue. The residue was dissolved in 2.7 mL dry MeOH and NH_4F (123.74 mg, 3.35 mmol, 12.30 equiv.) was added. The reaction was stirred at RT over 22 h. The solvent was removed under reduced pressure and the residue was suspended in 50 mL milli-Q water. The aqueous solution was extracted with 50 mL DCM four times and the combined organic layer was dried over Na_2SO_4, filtered and evaporated under reduced pressure. The crude product was purified with silica gel column chromatography using EtOAc and Cyclohexane as eluent system (20% to 50% EtOAc/Cyclohexane). The product **6** was afforded as white powder (166.4 mg, 77% isolated yield).

1H NMR (600 MHz, DMSO-*d*6**):** δ (ppm): 10.76 (s, 1H, NH), 7.60 – 7.03 (m, 20H, diphenylcarbonyl, C_6H_5), 5.92 (ddd, *J* = 7.8, 5.5, 2.3 Hz, 1H, 1'-H), 4.88 – 4.80 (m, 2H, 3'-O-CH_2SCH_3), 4.74 (ddt, *J* = 9.3, 6.9, 3.6 Hz, 1H, 3'-H), 4.65 – 4.58 (m, 1H, 5'-OH), 3.79 (qd, *J* = 6.0, 5.5, 2.3 Hz, 1H, 4'-H), 3.50 – 3.34 (m, 2H, 5'-H), 2.90 (br, s, 1H, 2'-H), 2.70 (h, *J* = 6.9 Hz, 1H, isobutyryl, CH), 2.25 – 2.18 (m, 1H, 2'-H), 1.07 (dd, *J* = 6.9, 5.7 Hz, 6H, isobutyryl, CH_3).

13C NMR (151 MHz, DMSO**):** δ (ppm): 174.80 (isobutyryl, CO), 152.17 (C2), 151.98 (C8), 149.86 (C6 or diphenylcarbonyl, CO), 148.48 (C6 or diphenylcarbonyl, CO), 147.30 (C4), 145.73 (C6 or diphenylcarbonyl, CO), 142.09 (diphenylcarbonyl, C_6H_5), 129.45 (diphenylcarbonyl, C_6H_5), 129.25 (diphenylcarbonyl, C_6H_5), 129.18 (diphenylcarbonyl, C_6H_5), 127.84 (diphenylcarbonyl, C_6H_5), 127.13 (diphenylcarbonyl, C_6H_5), 127.02 (diphenylcarbonyl, C_6H_5), 126.39 (diphenylcarbonyl, C_6H_5), 106.05 (C5), 85.18 (C4'), 81.39 (3'-O-CH_2N_3), 81.24 (C1'), 78.61 (C3'), 61.60 (C5'), 34.64 (isobutyryl, CH), 33.42 (C2'), 19.18 (isobutyryl, CH_3), 19.02 (isobutyryl, CH_3).

HR-MS (ESI): calcd for $(C41H38N10NaO8)^+$ $[M+Na]^+$: 821.2772, found: 821.2771.

Compound 7: 3'-O-azidomethyl-8-oxo-dGMP:
((2*R*,3*S*,5*R*)-5-(2-amino-6,8-dioxo-1,6,7,8-tetrahydro-9*H*-purin-9-yl)-3-(azidomethoxy)tetrahydrofuran-2-yl)methyl dihydrogen phosphate

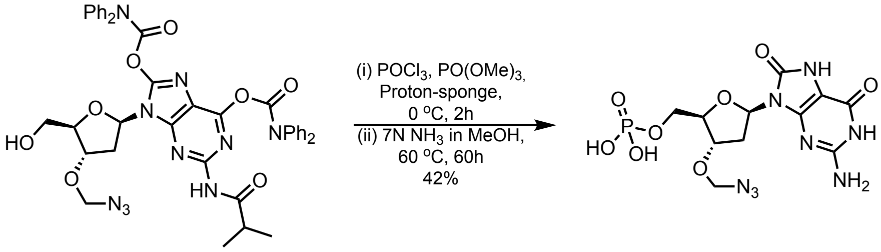

6 **7**

Compound **6** (59.8 mg, 74.86 µmol, 1.00 equiv.) and proton sponge (96.26 mg, 0.449mmol, 6.00 equiv.) were dried over high vacuum for 30 min. Under argon atmosphere mixture was dissolved in 0.2 mL trimethyl phosphate and cooled at 0 ºC. $POCl_3$ (27.8 µL, 0.299 mmol, 4.00 equiv.) was added dropwise and reaction was stirred over 2 h. Quenching of reaction was performed by adding 2.4 mL of ice-cold 1.0M TEAA buffer (pH= 7.6)

and the turbid solution was allowed to stir over 1 h at 0 ºC. Solvent was evaporated under reduced pressure and filter column (silica gel column chromatography) was used to remove the proton sponge using 0 to 5%MeOH/DCM/2%TEA eluent system, the rest residue was eluted with max 15%MeOH/DCM/2%TEA. Solvent was removed under reduced pressure. 5.5 mL 7N NH_3 in MeOH was added to the residue and was stirred at 60 ºC over 60 h. After removing solvent under reduced pressure, the crude was purified with HPLC using VP nucleodur C18 column (eluents A and B). Purified product was lyophilized to obtain 3'-O-azidomethyl-8-oxo-dGMP **7** as a TEA salt with 1:3.6:1.19 (compound **7**: TEA: AA (acetic acid) and 49wt%) mol ratio (26.9 mg, 31.50 µmol, 42% with TEA salt).

1H NMR (600 MHz, D_2O): δ (ppm): 6.19 (td, *J* = 7.3, 1.9 Hz, 1H, 1'-H), 4.96 (d, *J* = 9.2 Hz, 1H, 3'-O-CH_2N_3), 4.85 (d, *J* = 9.1 Hz, 1H, 3'-O-CH_2N_3), 4.69 (dq, *J* = 4.8, 2.4, 1.5 Hz, 1H, 3'-H), 4.25 (td, *J* = 6.3, 3.4 Hz, 1H, 4'-H), 4.05 (dtd, *J* = 11.9, 6.0, 1.0 Hz, 1H, 5'-H), 3.90 (ddd, *J* = 10.9, 6.5, 5.1 Hz, 1H, 5'-H), 3.30 (dddd, *J* = 14.2, 7.9, 6.5, 1.4 Hz, 1H, 2'-H), 3.20 (qd, *J* = 7.4, 1.4 Hz, 21.61H, Et_3NH^+), 2.43 (ddd, *J* = 14.1, 7.1, 3.4 Hz, 1H, 2'-H), 1.91 (s, 3.51H, CH_3COO^-), 1.28 (td, *J* = 7.3, 1.1 Hz, 32.48H, Et_3NH^+).
31P NMR (243 MHz, D_2O): δ (ppm): 3.81.
13C NMR (151 MHz, D_2O): δ (ppm): 181.45 (d, CH_3COO^-), 154.14 (C6 or C2), 153.03 (d, C8 or C6 or C2), 148.66(d, C4), 99.83 (C5), 83.67 (d, C4'), 81.72 (C1' and 3'-O-CH_2N_3), 79.52 (C3'), 63.77 (C5'), 46.62 (Et_3NH^+), 33.13 (C2'), 23.23(CH_3COO^-), 8.19 (Et_3NH^+)).
13C NMR (176 MHz, D_2O): δ (ppm): 181.32 (CH_3COO^-), 153.36 (C6 or C2), 153.15 (C6 or C2), 152.99 (C8), 148.69 (C4), 99.63 (C5), 83.27 (d, C4'), 81.82 (C1'), 81.73 (3'-O-CH_2N_3), 79.2 (C3'), 64.43 (d, C5'), 46.64 (Et_3NH^+), 33.26 (C2'), 23.14 (CH_3COO^-), 8.19 (Et_3NH^+)).
HR-MS (ESI): calcd for $(C11H14N8O8P)^-$ $[M-H]^-$: 417.0678, found: 417.0665.

Compound 8: 3'-O-azidomethyl-8-oxo-dGMP morpholidate:
((2*R*,3*S*,5*R*)-5-(2-amino-6,8-dioxo-1,6,7,8-tetrahydro-9*H*-purin-9-yl)-3-(azidomethoxy)tetrahydrofuran-2-yl)methyl hydrogen morpholinophosphonate

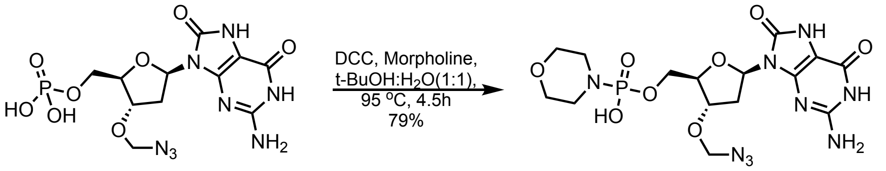

7 **8**

3'-O-azidomethyl-8-oxo-dGMP **7** with TEA salt (17 mg, 23.037 µmol, 1.00 equiv.) was dissolved in 0.43 mL milli-Q water and the pH of the solution was adjusted 3-4 by adding 1M HCl. Anhydrous morpholine (10 µL, 0.115 mmol, 5.00 equiv.) dissolved in 0.43 mL *t*-BuOH was added dropwise. The reaction mixture was allowed to stir at RT over 15 min, and warmed to 95 ºC. DCC (23.78 mg, 0.115 mmol, 5.00 equiv.) dissolved in 0.85mL *t*-BuOH was added dropwise over 2 h under same condition. Solution was further stirred over 2.5 h at 95 °C. The solvents were evaporated under reduced pressure, and resuspended in 1 mL of ice-cold water. Dicyclohexylurea precipitates were removed by filtration of the suspension using 0.45 µm HPLC Filter Chromafil. Filtrate was purified with HPLC using VP nucleodur C18 column (eluent A and B) and lyophilized to obtain 3'-

O-azidomethyl-8-oxo-dGMP morpholidate **8** as a TEA salt with 1:1.93:1.12 (compound **8**: TEA: AA and 64.9wt%) mol ratio (13.6 mg, 18.12 µmol, 79% with TEA salt).

1H NMR (700 MHz, D_2O): δ (ppm): 6.20 (tt, *J* = 7.8, 3.7 Hz, 1H, 1'-H), 4.93 – 4.86 (m, 2H, 3'-O-CH_2N_3), 4.78 – 4.74 (m, 1H, 3'-H), 4.26 – 4.21 (m, 1H, 4'-H), 4.09 – 4.03 (m, 1H, 5'-H), 3.97 (tdd, *J* = 11.1, 7.3, 5.0 Hz, 1H, 5'-H), 3.55 (tq, *J* = 11.4, 6.0 Hz, 4H, morpholine, O-CH_2), 3.31 (dtt, *J* = 14.3, 7.3, 1.6 Hz, 1H, 2'-H), 3.20 (q, *J* = 7.4 Hz, 11.55H, Et_3NH^+), 3.00 – 2.88 (m, 4H, morpholine, N-CH_2), 2.53 – 2.48 (m, 1H, 2'-H), 1.93 – 1.91 (m, 3.35H, CH_3COO^-), 1.28 (t, *J* = 7.3 Hz, 17.35H, Et_3NH^+).

31P NMR (283 MHz, D_2O): δ (ppm): 7.46.

13C NMR (176 MHz, D_2O): δ (ppm): 181.33(CH_3COO^-), 153.29 (C6 or C2), 153.03 (C6 or C2), 152.93 (C8), 148.57 (C4), 99.52 (C5), 83.26(d, C4'), 81.98 (3'-O-CH_2N_3), 81.62 (C1'), 78.89 (C3'), 66.78(d, morpholine, O-CH_2), 64.49(d, C5'), 46.64 (Et_3NH^+), 44.61 (morpholine, N-CH_2), 33.67 (C2'), 23.17(CH_3COO^-), 10.49 (Et_3NH^+), 8.19 (Et_3NH^+).

HR-MS (ESI): calcd for $(C15H21N9O8P)^-$ $[M-H]^-$: 486.1256, found: 486.1243.

Compound 9: 3'-O-azidomethyl-8-oxo-dGTP:
((2*R*,3*S*,5*R*)-5-(2-amino-6,8-dioxo-1,6,7,8-tetrahydro-9*H*-purin-9-yl)-3-(azidomethoxy)tetrahydrofuran-2-yl)methyl triphosphate

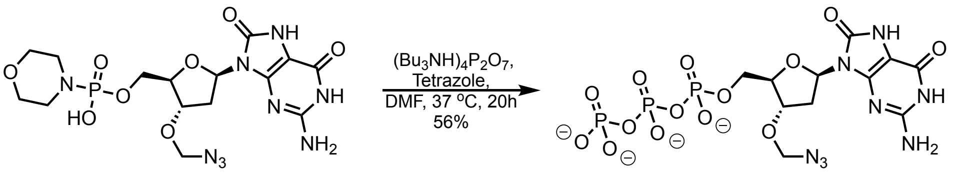

8 Compound **9**

3'-O-azidomethyl-8-oxo-dGMP morpholidate **8** as TEA salt (12.1 mg, 15.59 µmol, 1.00 equiv.) was co-evaporated three times with 0.5 mL toluene and dried over high vaccum overnight. To the dried morpholidate **8** tributylammonium pyrophosphate (34.22 mg, 62.38 µmol, 4.00 equiv.) was added and were dried over high vacuum over 30min. 0.45M Tetrazole (173.3 µL, 77.97 µmol, 5.00 equiv.) and dry DMF (500 µL) were added to the dry mixture under argon atmosphere. The reaction mixture was stirred at 37 °C over 20 h and solvent was removed by lyophilizing the reaction mixture. The crude was first purified with HPLC using Ion-exchange column with eluents C and D. Purified fraction was lyophilized and salt exchange was performed with VP nucleodur C18 column using eluents A and B. Purified triphosphate was lyophilized to afford triphosphate **9** as a TEA salt with 1:1.70 (compound **9**: TEA and 77.1wt%) mol ratio (6.5 mg, 8.664 µmol, 56% with TEA salt).

1H NMR (600 MHz, D_2O): δ (ppm): 6.21 (t, *J* = 7.3 Hz, 1H, 1'-H), 4.97 (d, *J* = 9.2 Hz, 1H, 3'-O-CH_2N_3), 4.87 (d, *J* = 9.2 Hz, 1H, 3'-O-CH_2N_3), 4.78 – 4.74 (m, 1H, 3'-H), 4.32 (h, *J* = 6.0 Hz, 2H, 4'-H and 5'-H), 4.18 (dt, *J* = 9.6, 6.3 Hz, 1H, 5'-H), 3.33 (dt, *J* = 14.2, 7.1 Hz, 1H, 2'-H), 3.21 (q, *J* = 7.3 Hz, 10.08H, Et_3NH^+), 2.45 (ddd, *J* = 14.1, 7.0, 3.1 Hz, 1H, 2'-H), 1.29 (t, *J* = 7.3 Hz, 15.52H, Et_3NH^+).

31P NMR (243 MHz, D_2O): δ (ppm): -10.36 (d, *J* = 19.5 Hz), -11.27 (d, *J* = 19.9 Hz), -23.08 (t, *J* = 19.6 Hz).

13C NMR (151MHz, D₂O): δ (ppm): 153.35 (C6 or C5), 153.18 (C6 or C5), 153.08 (C8), 148.83 (C4), 99.63 (C5), 83.02 (d, C4‘), 82.02 (C1‘), 81.81 (3’-O-CH_2N_3), 79.46 (C3’), 65.36 (d, C5’), 46.63 (Et_3NH^+), 33.26 (C2’), 8.20 (Et_3NH^+).

HR-MS (ESI): calcd for $(C11H16N8O14P3)^-$ $[M-H]^-$: 577.0004, found: 576.9994.

Synthesis of 3’-O-azidomethyl-8-oxo-deoxyadenosine triphosphate

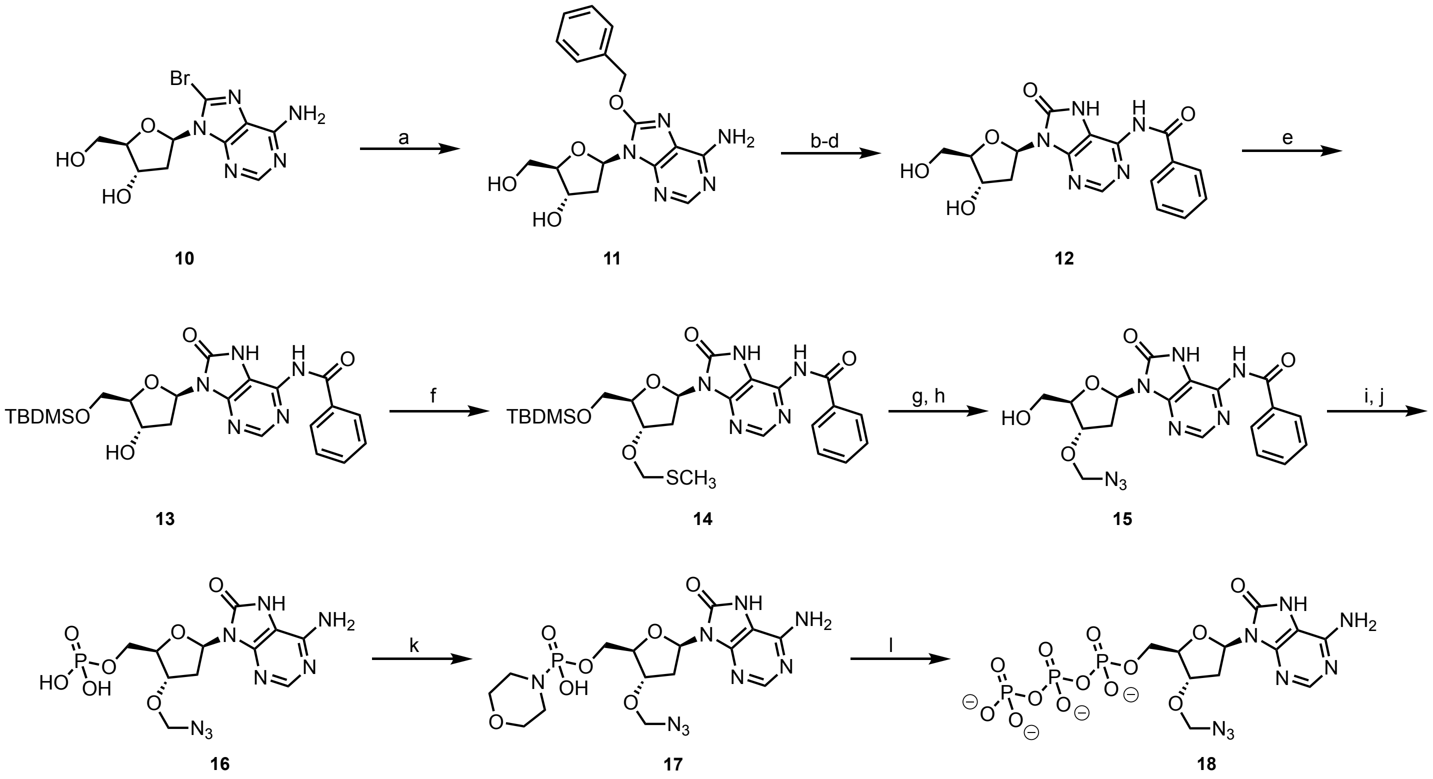

Compound 11:

(2R,3S,5R)-5-(6-amino-8-(benzyloxy)-9*H*-purin-9-yl)-2(hydroxymethyl)tetrahydrofuran-3-ol

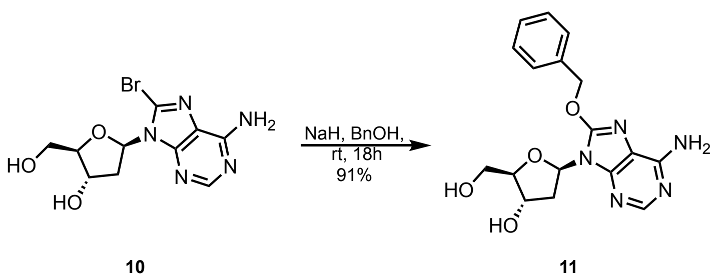

In an inert flask under argon atmosphere NaH (355.27 mg, 14.8 mmol, 60wt%, 5.75 equiv.) and 12.75 mL benzyl alcohol were stirred at RT over 2.5 h. 8-bromo-2’-deoxyadenosine **10** (850 mg, 2.57 mmol, 1.00 equiv.) was

added to the prepared sodium benzoylate and reaction was allowed to stir at RT over 15.5 h. Quenching of reaction was performed by neutralizing the reaction mixture with concentrated HCl in ice bath. 100 mL EtOAc was added to the solution and, organic layer was washed twice with saturated $NaHCO_3$ and brine. Organic layer was dried over $MgSO_4$, filtered and solvent evaporated under reduce pressure to afford viscous liquid. Silica gel column chromatography using MeOH and DCM as eluent system (0 to 10%, MeOH/DCM) yielded product **11** as white solid (834.4 mg, 91% isolated yield).

1H NMR (500 MHz, DMSO-*d*6): δ (ppm): 8.05 (s, 1H, 2-H), 7.58 – 7.52 (m, 2H, 8-O-CH_2-C_6H_5), 7.47 – 7.35 (m, 3H, 8-O-CH_2-C_6H_5), 6.97 (s, 2H, 6-NH_2), 6.21 (dd, *J* = 8.2, 6.4 Hz, 1H, 1'-H), 5.60 – 5.50 (m, 2H, 8-O-CH_2-Ph), 5.31 – 5.23 (m, 2H, 3'-OH & 5'-OH), 4.41 – 4.34 (m, 1H, 3'-H), 3.82 (td, *J* = 4.7, 2.5 Hz, 1H, 4'-H), 3.57 (dt, *J* = 11.8, 4.6 Hz, 1H, 5'-H), 3.47 – 3.41 (m, 1H, 5'-H), 3.07 – 2.97 (m, 1H, 2'-H), 2.11 (ddd, *J* = 13.1, 6.5, 2.7 Hz, 1H, 5'-H).

13C NMR (126 MHz, DMSO-*d*6): δ (ppm): 154.06 (C4), 153.44(C8), 150.66 (C2), 148.70 (C6), 135.41(C_6H_5), 128.62 (C_6H_5), 128.49 (C_6H_5), 114.88 (C5), 87.93 (C4'), 82.60 (C1'), 71.35 (8-O-CH_2-Ar), 71.27(C3'), 62.33 (C5'), 36.94 (C2').

HR-MS (ESI): calcd for $(C_{17}H_{20}N_5O_4)^+$ $[M+H]^+$: 358.1515, found: 358.1510.

Compound 12:

N-(9-((2*R*,4*S*,5*R*)-4-hydroxy-5-(hydroxymethyl)tetrahydrofuran-2-yl)-8-oxo-8,9-dihydro-7*H*-purin-6-yl)benzamide

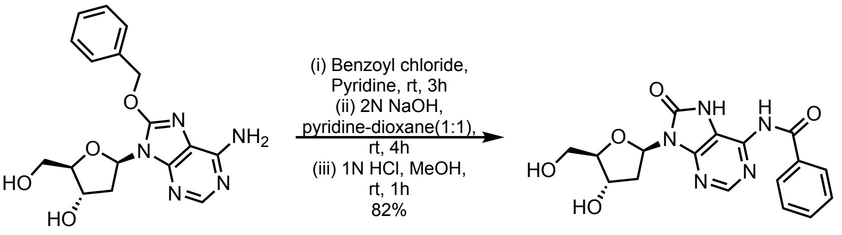

11 **12**

In an inert flask under argon atmosphere compound **11** (442.5 mg, 1.24 mmol, 1.00 equiv.) was dissolved in 18.2 mL dry pyridine. At 4 °C benzoyl chloride (1.03 mL, 8.92 mmol, 7.20 equiv.) was added dropwise and reaction mixture was allowed to stir at RT over 3 h. 1 mL milli-Q water was added and solvent removed under reduced pressure. 50 mL DCM was added to the residual oil and organic layer was washed with saturated $NaHCO_3$, brine. Organic layer was dried over $MgSO_4$, filtered and solvent removed under reduced pressure to afford tetrabenzoylated intermediate. Crude mixture dissolved in a 65.4 mL dioxane: pyridine (1:1) solution and 36.3 mL of 2N NaOH solution was added. After 4 h of stirring at RT, reaction was quenched by neutralizing with 1M HCl. The aqueous layer was extracted four times with DCM and the combined organic layers were washed with saturated $NaHCO_3$ solution, dried over Na_2SO_4, filtered and solvent evaporated under reduced pressure. The crude product was further dissolved in 14.5 mL MeOH and 1M HCl (1.43 mL, 1.43 mmol, 1.16 equiv.) was added at room temperature. The solution was stirred at RT over 1 h and reaction mixture was neutralized the pH with 2M NaOH solution. Solvent was evaporated under reduced pressure and silica gel column

chromatography was used to purify the crude using MeOH and DCM as eluent system (0 to 8%, MeOH/DCM) to obtain product **12** as white solid (376.1 mg, 82% isolated yield).

1H NMR (500 MHz, DMSO-*d*6): δ (ppm): 11.20 (s, 1H, NH), 10.73 (s, 1H, NH), 8.49 (s, 1H, 2-H), 8.09 – 8.01 (m, 2H, 6-NH-COC_6H_5), 7.68 – 7.60 (m, 1H, 6-NH-COC_6H_5), 7.55 (dd, *J* = 8.4, 7.1 Hz, 2H, 6-NH-COC_6H_5), 6.25 (t, *J* = 7.3 Hz, 1H, 1'-H), 5.24 (d, *J* = 4.3 Hz, 1H, 3'-OH), 4.89 – 4.81 (m, 1H, 5'-OH), 4.45 (dq, *J* = 6.7, 3.4 Hz, 1H, 3'-H), 3.82 (td, *J* = 5.3, 2.8 Hz, 1H, 4'-H), 3.68 – 3.59 (m, 1H, 5'-H), 3.54 – 3.43 (m, 1H, 5'-H), 3.10 (ddd, *J* = 13.5, 7.8, 6.2 Hz, 1H, 2'-H), 2.10 (ddd, *J* = 13.1, 6.8, 3.2 Hz, 1H, 2'-H).

13C NMR (126 MHz, DMSO-*d*6): δ (ppm): 165.46 (CO), 151.39 (C8), 150.24 (C4), 149.47 (C2), 138.40 (C6), 132.92 (C_6H_5), 132.40 (C_6H_5), 128.47 (C_6H_5), 128.37 (C_6H_5), 112.77 (C5), 87.53 (C4'), 81.30 (C1'), 71.16 (C3'), 62.26 (C5'), 35.64 (C2').

HR-MS (ESI): calcd for $(C17H18N5O5)^+$ $[M+H]^+$: 372.1308, found: 372.1313.

Compound 13:

N-(9-((2*R*,4*S*,5*R*)-5-(((*tert*-butyldimethylsilyl)oxy)methyl)-4-hydroxytetrahydrofuran-2-yl)-8-oxo-8,9-dihydro-7*H*-purin-6-yl)benzamide

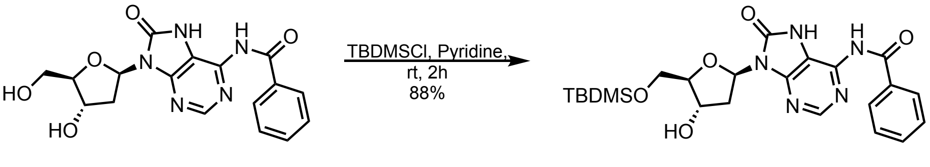

12 **13**

In an inert Schlenk flask under argon atmosphere compound **12** (576.6 mg, 1.55 mmol, 1.00 equiv.) was dissolved in 3.1 mL dry pyridine. TBDMSCl (351 mg, 2.33 mmol, 1.50 equiv.) was added and reaction stirred at RT. After 1.5 h again TBDMSCl (117 mg, 0.776 mmol, 0.50 equiv.) was added and the reaction mixture was stirred at RT over 30 min. The reaction was quenched by adding 1ml MeOH.

The solvent was removed under reduced pressure. The residue was dissolved in 50 mL ethyl acetate and washed with 50 mL of sat. $NaHCO_3$, water, brine. The organic layer was dried over $MgSO_4$, filtered and evaporated under reduced pressure. The resulted crude product was purified with silica gel column chromatography using MeOH and DCM as eluent system (0% to 5% MeOH/DCM). The protected product **13** was afforded as white solid (660 mg, 88% isolated yield).

1H NMR (700 MHz, DMSO-*d*6): δ (ppm): 11.18 (s, 1H, NH), 10.68 (s, 1H, NH), 8.48 (s, 1H, 2-H), 8.08 – 8.03 (m, 2H, 6-NH-COC_6H_5), 7.66 – 7.61 (m, 1H, 6-NH-COC_6H_5), 7.57 – 7.52 (m, 2H, 6-NH-COC_6H_5), 6.23 (t, *J* = 7.1 Hz, 1H, 1'-H), 5.26 (d, *J* = 4.5 Hz, 1H, 3'-OH), 4.48 (dq, *J* = 7.8, 4.0 Hz, 1H, 3'-H), 3.84 (dd, *J* = 10.8, 5.6 Hz, 1H, 5'-H), 3.79 (td, *J* = 5.8, 3.6 Hz, 1H, 4'-H), 3.66 (dd, *J* = 10.8, 6.1 Hz, 1H, 5'-H), 3.17 (dt, *J* = 13.2, 6.6 Hz, 1H, 2'-H), 2.12 (ddd, *J* = 13.1, 7.3, 4.0 Hz, 1H, 2'-H), 0.84 (s, 9H, 5'-O-$Si(CH_3)_2C(CH_3)_3$), -0.01 (d, *J* = 4.2 Hz, 6H, 5'-O-$Si(CH_3)_2C(CH_3)_3$).

13C NMR (176 MHz, DMSO-*d*6): δ (ppm): 165.40 (CO), 151.40 (C8), 150.31 (C4), 149.46 (C2), 138.26 (C6), 132.93 (C_6H_5), 132.37 (C_6H_5), 128.45 (C_6H_5), 128.36 (C_6H_5), 112.70 (C5), 86.75 (C4'), 80.99 (C1'), 70.65 (C3'), 63.51 (C5'), 35.04 (C2'), 25.81(5'-O-$Si(CH_3)_2C(CH_3)_3$), 18.04 (5'-O-$Si(CH_3)_2C(CH_3)_3$), -5.27 (5'-O-$Si(CH_3)_2C(CH_3)_3$), -5.39 (5'-O-$Si(CH_3)_2C(CH_3)_3$).

HR-MS (ESI): calcd for $(C23H31N5NaO5Si)^+$ $[M+Na]^+$: 508.1992, found: 508.1970.

Compound 14:

N-(9-((2*R*,4*S*,5*R*)-5-(((*tert*-butyldimethylsilyl)oxy)methyl)-4-((methylthio)methoxy)tetrahydrofuran-2-yl)-8-oxo-8,9-dihydro-7*H*-purin-6-yl)benzamide

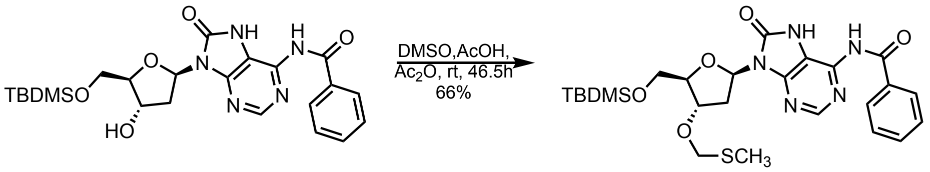

13 **14**

In an inert Schlenk flask under argon atmosphere compound 1**3** (488.2 mg, 1.01 mmol, 1.00 equiv.) was dissolved in 1.9 mL dry DMSO, 0.87 mL acetic acid and 2.8 mL acetic anhydride were added to the solution. The reaction was allowed to stir at RT over 46.5 h. Quenching of the reaction was performed by adding 50 mL saturated $NaHCO_3$ solution. The aqueous layer was washed four times with ethyl acetate and the combined organic layer was washed with saturated $NaHCO_3$ solution. The organic layer was dried over Na_2SO_4, filtered and evaporated under reduced pressure to afford crude product. Silica gel column chromatography using Et_2O and n-Pentane as eluent system (20 to 60% Et_2O/n-Pentane) yielded product **14** as white foamy solid (364 mg, 66% isolated yield).

1H NMR (500 MHz, DMSO-*d*₆) δ (ppm): 11.18 (s, 1H, NH), 10.73 (s, 1H, NH), 8.49 (s, 1H, 2-H), 8.08 – 8.03 (m, 2H, 6-NH-COC_6H_5), 7.66 – 7.61 (m, 1H, 6-NH-COC_6H_5), 7.55 (dd, *J* = 8.4, 7.1 Hz, 2H, 6-NH-COC_6H_5), 6.21 (t, *J* = 7.2 Hz, 1H, 1'-H), 4.74 (s, 2H, 3'-O-CH_2SCH_3), 4.65 (dt, *J* = 6.2, 2.9 Hz, 1H, 3'-H), 3.96 (ddd, *J* = 7.0, 5.8, 2.7 Hz, 1H, 4'-H), 3.82 (dd, *J* = 10.6, 7.1 Hz, 1H, 5'-H), 3.68 (dd, *J* = 10.6, 5.9 Hz, 1H, 5'-H), 3.31 – 3.28 (m, 1H, 2'-H), 2.32 (ddd, *J* = 13.6, 7.0, 3.0 Hz, 1H, 2'-H), 2.13 (s, 3H, 3'-O-CH_2SCH_3), 0.86 (s, 9H, 5'-O-$Si(CH_3)_2C(CH_3)_3$), 0.02 (s, 6H, 5'-O-$Si(CH_3)_2C(CH_3)_3$).

13C NMR (126 MHz, DMSO-*d*₆): δ (ppm): 165.44 (CO), 151.34 (C8), 150.27 (C4), 149.49 (C2), 138.39 (C6), 132.92 (C_6H_5), 132.40 (C_6H_5), 128.46 (C_6H_5), 128.37 (C_6H_5), 112.70 (C5), 84.25 (C4'), 81.44 (C1'), 76.64 (C3'), 72.66 (3'-O-CH_2SCH_3), 63.03 (C5'), 31.94 (C2'), 25.76 (5'-O-$Si(CH_3)_2C(CH_3)_3$), 17.95 (5'-O-$Si(CH_3)_2C(CH_3)_3$), 13.33 (3'-O-CH_2SCH_3), -5.40 (5'-O-$Si(CH_3)_2C(CH_3)_3$), -5.44 (5'-O-$Si(CH_3)_2C(CH_3)_3$).

HR-MS (ESI): calcd for $(C25H35N5NaO5SSi)^+$ $[M+Na]^+$: 568.2026, found: 568.2020.

Compound 15:

N-(9-((2*R*,4*S*,5*R*)-4-(azidomethoxy)-5-(hydroxymethyl)tetrahydrofuran-2-yl)-8-oxo-8,9-dihydro-7*H*-purin-6-yl)benzamide

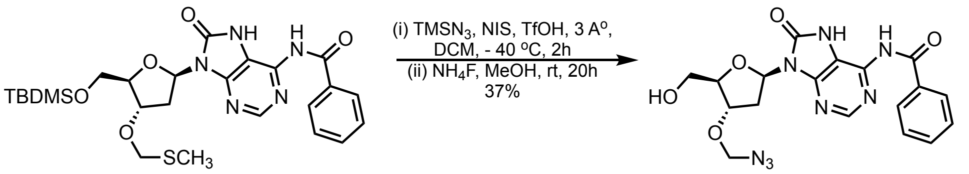

14 **15**

In an inert Schlenk flask under argon atmosphere compound **14** (363.2 mg, 0.666 mmol, 1.00 equiv.) with 3 A° MS was dissolved in dry 6.7 mL DCM. $TMSN_3$ (264 µL, 2 mmol, 3.00 equiv.) was added and stirred at RT over 10 min. The reaction mixture was cooled to - 40 ºC, NIS (299.5 mg, 1.33 mmol, 2.00 equiv.) and TfOH (88.1 µL, 0.998 mmol, 1.50 equiv.) were added. The reaction mixture was allowed to stir under same conditions over 2h. Quenching of the reaction was performed by adding 5 mL saturated $NaHCO_3$ solution at 0 ºC, filtered, and the filtrate was mixed with 40 mL DCM washed with saturated $Na_2S_2O_3$ and brine. The organic layer was dried over $MgSO_4$, filtered and evaporated under reduced pressure. The residue was dissolved in 11.7 mL dry MeOH and NH_4F (295.80 mg, 7.99 mmol, 12.00 equiv.) was added. The reaction was stirred at RT over 20 h. The solvent was removed under reduced pressure and the residue was suspended in 50 mL milli-Q water. The aqueous solution was extracted with 50 mL DCM four times and the combined organic layer was dried over Na_2SO_4, filtered and evaporated under reduced pressure. The crude product was purified with silica gel column chromatography using EtOAc and Cyclohexane as eluent system (20% to 70% EtOAc/Cyclohexane). The product **15** was afforded as white solid (105.6 mg, 37% isolated yield).

1H NMR (700 MHz, Chloroform-*d*): δ (ppm): 9.60 (s, 1H, NH), 8.61 (s, 1H, NH), 8.40 (s, 1H, 2-H), 7.98 – 7.92 (m, 2H, 6-NH-COC_6H_5), 7.69 – 7.64 (m, 1H, 6-NH-COC_6H_5), 7.60 – 7.54 (m, 2H, 6-NH-COC_6H_5), 6.46 (dd, *J* = 9.7, 5.8 Hz, 1H, 1'-H), 5.40 (d, *J* = 11.6 Hz, 1H, 5'-OH), 4.80 (d, *J* = 9.1 Hz, 1H, 3'-O-CH_2N_3), 4.71 (d, *J* = 9.1 Hz, 1H, 3'-O-CH_2N_3), 4.67 – 4.63 (m, 1H, 3'-H), 4.29 (q, *J* = 1.7 Hz, 1H, 4'-H), 3.97 (dt, *J* = 12.6, 1.6 Hz, 1H, 5'-H), 3.77 (td, *J* = 12.3, 1.9 Hz, 1H, 5'-H), 2.98 (ddd, *J* = 13.6, 9.7, 5.7 Hz, 1H, 2'-H), 2.37 (ddd, *J* = 13.6, 5.8, 1.2 Hz, 1H, 2'-H).
13C NMR (176 MHz, Chloroform-*d*): δ (ppm): 165.95 (CO), 150.88 (C8), 150.60 (C4), 150.13 (C2), 138.01 (C6), 133.77 (C_6H_5), 131.98 (C_6H_5), 129.37 (C_6H_5), 127.80 (C_6H_5), 109.36 (C5), 86.63 (C4'), 83.61 (C1'), 81.65 (3'-O-CH_2N_3), 80.21 (C3'), 63.84(C5'), 35.96(C2').
HR-MS (ESI): calcd for $(C18H18N8NaO5)^+$ $[M+Na]^+$: 449.1298, found: 449.1291.

Compound 16: 3'-O-azidomethyl-8-oxo-dAMP:
((2*R*,3*S*,5*R*)-5-(6-amino-8-oxo-7,8-dihydro-9*H*-purin-9-yl)-3-(azidomethoxy)tetrahydrofuran-2-yl)methyl dihydrogen phosphate

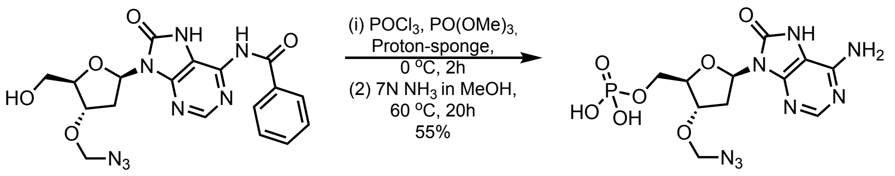

15 16

Compound **15** (51.8 mg, 121.48 µmol, 1.00 equiv.) and proton sponge (156.21 mg, 0.729mmol, 6.00 equiv.) were dried over high vacuum for 30 min. Under argon atmosphere mixture was dissolved in 0.51 mL trimethyl phosphate and cooled at 0 ºC. $POCl_3$ (45.3 µL, 0.486 mmol, 4.00 equiv.) was added dropwise and reaction was stirred over 2 h. Quenching of reaction was performed by adding 3.9 mL of ice-cold 1.0M TEAA buffer (pH= 7.6) and the turbid solution was allowed to stir over 1 h at 0 ºC. Solvent was evaporated under reduced pressure and filter column (silica gel column chromatography) was used to remove the proton sponge using 0 to 5%MeOH/DCM/2%TEA eluent system, the rest residue was eluted with max 15%MeOH/DCM/2%TEA. Solvent

was removed under reduced pressure. 8.94 mL 7N NH_3 in MeOH was added to the residue and was stirred at 60 ºC over 20 h. After removing solvent under reduced pressure, the crude was purified with HPLC using VP nucleodur C18 column ((eluent A and B). Purified product was lyophilized to obtain 3'-O-azidomethyl-8-oxo-dAMP **16** as a TEA salt with 1:3.10:2.75 (compound **16**: TEA: AA and 46wt%) mol ratio (59 mg, 67.04 µmol, 55% with TEA salt).

1H NMR (600 MHz, D_2O): δ (ppm): 8.14 – 8.05 (m, 1H, 2-H), 6.32 – 6.24 (m, 1H, 1'-H), 4.89 (ddt, *J* = 31.6, 9.2, 1.2 Hz, 2H, 3'-O-CH_2N_3), 4.71 (tt, *J* = 7.8, 6.2, 2.8 Hz, 1H, 3'-H), 4.27 (ddq, *J* = 6.5, 4.7, 2.6, 1.8 Hz, 1H, 4'-H), 4.14 – 3.99 (m, 2H, 5'-H), 3.31 (ddt, *J* = 16.2, 9.3, 4.7 Hz, 1H, 2'-H), 3.24 – 3.15 (m, 18.33H, Et_3NH^+), 2.49 (ddt, *J* = 13.8, 8.9, 3.0 Hz, 1H, 2'-H), 1.97 (d, *J* = 2.2 Hz, 8.2H, CH_3COO^-), 1.27 (tt, *J* = 7.3, 1.4 Hz, 27.9H, Et_3NH^+).
31P NMR (243 MHz, D_2O): δ (ppm): 0.28.
13C NMR (151 MHz, D_2O): δ (ppm): 179.67(CH_3COO^-), 152.75 (C8), 151.00 (C2), 147.16 (C6), 146.55 (C4), 104.08 (C5), 83.04 (d, C4'), 81.87 (3'-O-CH_2N_3), 81.65 (C1'), 78.84 (C3'), 64.71 (d, C5'), 46.63 (Et_3NH^+), 33.24 (C2'), 22.21(CH_3COO^-), 8.19 (Et_3NH^+).
HR-MS (ESI): calcd for $(C11H14N8O7P)^-$ $[M-H]^-$: 401.0729, found: 401.0715.

Compound 17: 3'-O-azidomethyl-8-oxo-dAMP morpholidate:
((2*R*,3*S*,5*R*)-5-(6-amino-8-oxo-7,8-dihydro-9*H*-purin-9-yl)-3-(azidomethoxy)tetrahydrofuran-2-yl)methyl hydrogen morpholinophosphonate

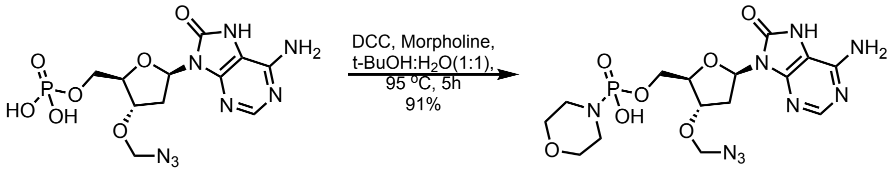

16 17

3'-O-azidomethyl-8-oxo-dAMP **16** with TEA salt (43.2 mg, 56.647 µmol, 1.00 equiv.) was dissolved in 0.9 mL milli-Q water and the pH of the solution was adjusted 3-4 by adding 1M HCl. Anhydrous morpholine (22 µL, 0.255 mmol, 4.50 equiv.) dissolved in 0.9 mL *t*-BuOH was added dropwise. The reaction mixture was allowed to stir at RT over 15 min, and warmed to 95 ºC. DCC (52.6 mg, 0.255 mmol, 4.50 equiv.) dissolved in 1.8 mL *t*-BuOH was added dropwise over 2 h under same condition. Solution was further stirred over 3 h at 95 °C. The solvents were evaporated under reduced pressure, and resuspended in 1 mL of ice-cold water. Dicyclohexylurea precipitates were removed by filtration of the suspension using 0.45 µm HPLC Filter Chromafil. Filtrate was purified with HPLC using VP nucleodur C18 column (eluent A and B) and lyophilized to obtain 3'-O-azidomethyl-8-oxo-dAMP morpholidate **17** as a TEA salt with 1:1.84:0.89(compound **17**: TEA: AA and 66wt%) mol ratio (36.5 mg, 51.32 µmol, 91% with TEA salt).

1H NMR (700 MHz, D_2O): δ (ppm): 8.13 – 8.08 (m, 1H, 2-H), 6.26 (qd, *J* = 7.7, 6.9, 3.7 Hz, 1H, 1'-H), 4.94 – 4.86 (m, 2H, 3'-O-CH_2N_3), 4.78 – 4.75 (m, 1H, 3'-H), 4.23 (pd, *J* = 4.0, 1.6 Hz, 1H, 4'-H), 4.02 (dtd, *J* = 10.5, 4.6, 1.3 Hz, 1H, 5'-H), 3.95 – 3.89 (m, 1H, 5'-H), 3.55 – 3.46 (m, 4H, morpholine, CH_2), 3.38 – 3.31 (m, 1H, 2'-H), 3.23 – 3.16 (m, 10.93H, Et_3NH^+), 2.88 (q, *J* = 4.8 Hz, 4H, morpholine, CH_2), 2.54 (dddd, *J* = 15.0, 9.1, 4.5, 2.8 Hz, 1H, 2'-H), 1.91 (s, 2.67H, CH_3COO^-), 1.28 (t, *J* = 7.4 Hz, 16.54H, Et_3NH^+).

31P NMR (243 MHz, D2O): δ (ppm): 7.36.

13C NMR (176 MHz, D2O): δ (ppm): 181.36 (CH_3COO^-), 152.76 (C8), 151.18 (C2), 147.34 (C6), 146.59 (C4), 104.08 (C5), 83.14 (d, C4'), 82.04 (3'-O-CH_2N_3), 81.62 (C1'), 78.68 (C3'), 66.74 (d, morpholine, CH_2), 64.36 (d, C5'), 46.63 (Et_3NH^+), 44.57(morpholine, CH_2), 33.53 (C2'), 23.21 (CH_3COO^-), 8.19 (Et_3NH^+).

HR-MS (ESI): calcd for $(C15H21N9O7P)^-$ $[M-H]^-$: 470.1307, found: 470.1294.

Compound 18: 3'-O-azidomethyl-8-oxo-dATP:
((2*R*,3*S*,5*R*)-5-(6-amino-8-oxo-7,8-dihydro-9*H*-purin-9-yl)-3-(azidomethoxy)tetrahydrofuran-2-yl)methyl triphosphate

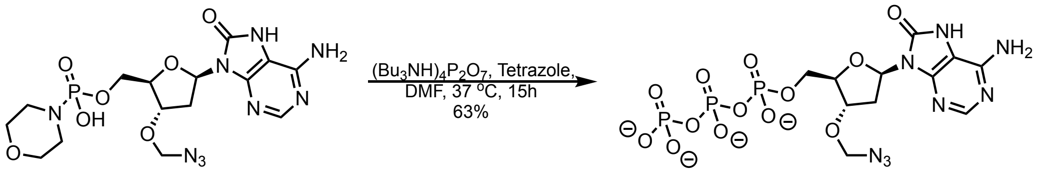

17 **18**

3'-O-azidomethyl-8-oxo-dAMP morpholidate **17** as TEA salt (5 mg, 8.84 µmol, 1.00 equiv.) was co-evaporated three times with 0.5 mL toluene and dried over high vaccum overnight. To the dried morpholidate **17** tributylammonium pyrophosphate (19.39 mg, 35.34 µmol, 4.00 equiv.) was added and were dried over high vacuum over 30min. 0.45M Tetrazole (98.2 µL, 44.18 µmol, 5.00 equiv.) and dry DMF (300 µL) were added to the dry mixture under argon atmosphere. The reaction mixture was stirred at 37 °C over 15 h and solvent was removed by lyophilizing the reaction mixture. The crude was first purified with HPLC using Ion-exchange column with eluents C and D. Purified fraction was lyophilized and salt exchange was performed with VP nucleodur C18 column using eluents A and B. Purified triphosphate was lyophilized to afford triphosphate **18** as a TEA salt with 1:1.75 (compound **18**: TEA and 76wt%) mol ratio (4.1 mg, 5.55 µmol, 63% with TEA salt).

1H NMR (700 MHz, D2O): δ (ppm): 8.16 (s, 1H, 2-H), 6.32 (t, J = 7.1 Hz, 1H, 1'-H), 4.98 (d, J = 9.2 Hz, 1H, 3'-O-CH_2N_3), 4.85 (d, J = 9.1 Hz, 1H, 3'-O-CH_2N_3), 4.74 (dt, J = 6.7, 4.0 Hz, 1H, 3'-H), 4.33 (ddd, J = 6.6, 5.2, 3.9 Hz, 1H, 4'-H), 4.23 (ddd, J = 11.6, 6.7, 5.2 Hz, 1H, 5'-H), 4.15 (dt, J = 11.0, 6.6 Hz, 1H, 5'-H), 3.38 (dt, J = 14.0, 6.9 Hz, 1H, 2'-H), 3.20 (q, J = 7.3 Hz, 10.58H, Et_3NH^+), 2.51 (ddd, J = 14.1, 7.3, 4.0 Hz, 1H, 2'-H), 1.28 (t, J = 7.3 Hz, 15.9H, Et_3NH^+).

31P NMR (283 MHz, D2O) with 1H decoupling: δ (ppm): -8.48, -11.20 (d, J = 19.0 Hz), -22.74.

31P NMR (243 MHz, D2O) without 1H decoupling: -10.08, -11.28 (dt, J = 19.5, 6.6 Hz), -23.06 (t, J = 19.8 Hz).

13C NMR (176 MHz, D2O): δ (ppm): 176.79 (CH_3COO^-), 153.02 (C8), 151.24 (C2), 147.51 (C6), 146.87 (C4), 104.49 (C5), 82.91 (d, C4'), 81.98 (3'-O-CH_2N_3), 81.74 (C1'), 79.09 (C3'), 65.43 (d, C5'), 46.62 (Et_3NH^+), 33.26 (C2'), 8.19 (Et_3NH^+).

HR-MS (ESI): calcd for $(C11H16N8O13P3)^-$ $[M-H]^-$: 561.0055, found: 561.0043.

Synthesis of 3'-O-azidomethyl-5-methyl-2'-deoxycytidine triphosphate

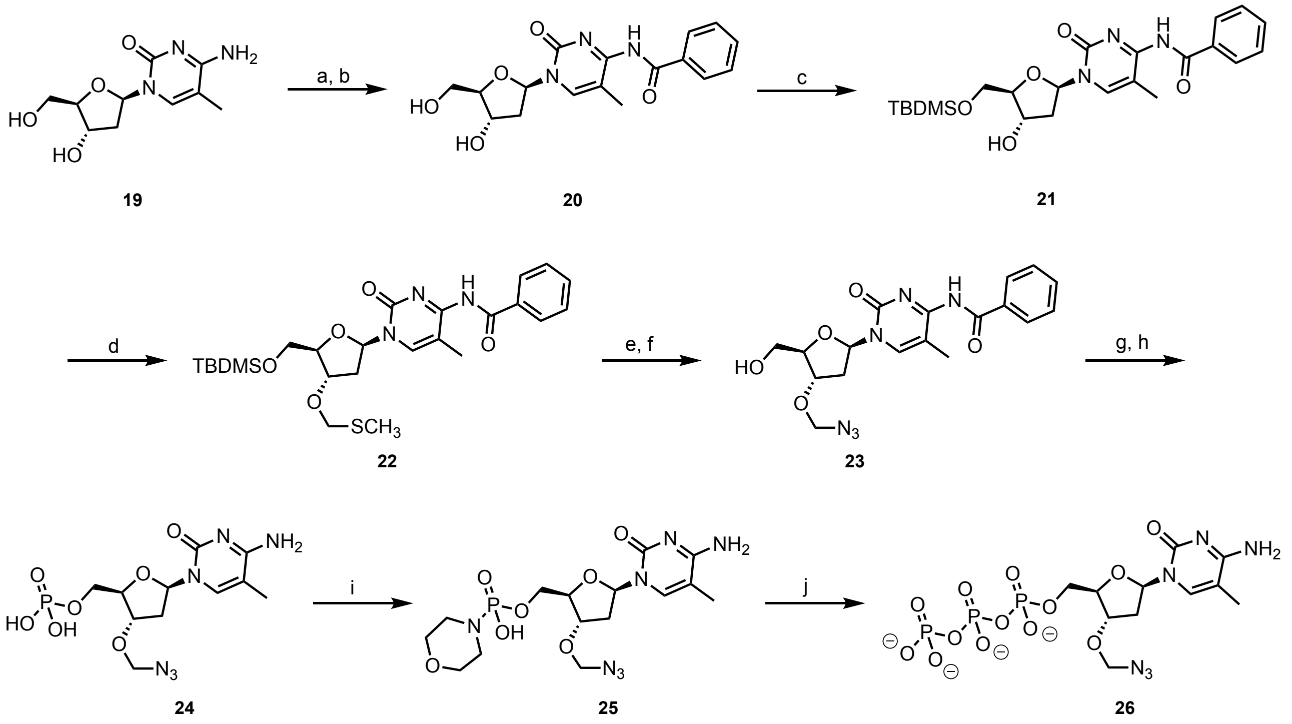

Compound 20:

N-(1-((2*R*,4*S*,5*R*)-4-hydroxy-5-(hydroxymethyl)tetrahydrofuran-2-yl)-5-methyl-2-oxo-1,2-dihydropyrimidin-4-yl)benzamide

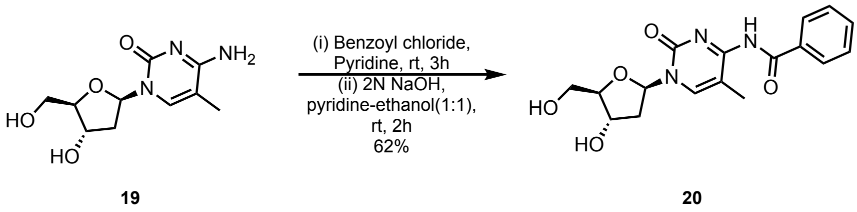

In an inert flask under argon atmosphere compound **19** (1.0 g, 4.15 mmol, 1.00 equiv.) was dissolved in 20.75 mL dry pyridine. At 4 °C benzoyl chloride (1.90 mL, 16.58 mmol, 4.00 equiv.) was added dropwise and reaction mixture was allowed to stir at RT over 3 h. 1 mL milli-Q water was added and solvent removed under reduced pressure. 50 mL DCM was added to the residual oil and organic layer was washed with saturated $NaHCO_3$, brine. Organic layer was dried over $MgSO_4$, filtered and solvent removed under reduced pressure to afford tetrabenzoylated intermediate. Crude mixture dissolved in a 50 mL pyridine: ethanol (1:1) solution and 25 mL of 2N NaOH solution was added. After 2 h of stirring at RT, reaction was quenched by neutralizing with 1M HCl. The aqueous layer was extracted four times with DCM and the combined organic layers were washed with saturated $NaHCO_3$ solution, dried over Na_2SO_4, filtered and solvent evaporated under reduced pressure. The crude product was purified with silica gel column chromatography using MeOH and DCM as eluent system (0 to 6%, MeOH/DCM) to obtain product **20** as white solid (889.9 mg, 62% isolated yield).

1H NMR (600 MHz, DMSO-*d*6): δ (ppm): 13.02 (s, 1H, NH), 8.19 (s, 2H, 4-NH-COC$_6$H$_5$), 8.08 (s, 1H, 6-H), 7.59 (t, *J* = 7.5 Hz, 1H, 4-NH-COC$_6$H$_5$), 7.49 (t, *J* = 7.1 Hz, 2H, 4-NH-COC$_6$H$_5$), 6.17 (t, *J* = 6.5 Hz, 1H, 1'-H), 5.28 (dd, *J* = 4.3, 1.2 Hz, 1H, 3'-OH), 5.12 (t, *J* = 5.1 Hz, 1H, 5'-OH), 4.31 – 4.24 (m, 1H, 3'-H), 3.83 (br s, 1H, 4'-H), 3.70 – 3.56 (m, 2H, 5'-H), 2.24 – 2.12 (m, 2H, 2'-H), 2.02 (s, 3H, 5-CH3).

13C NMR (151 MHz, DMSO-*d*6): δ (ppm): 178.16 (CO), 159.17 (C4), 147.44 (C2), 138.94 (C6), 136.76 (C$_6$H$_5$), 132.50 (C$_6$H$_5$), 129.35 (C$_6$H$_5$), 128.31 (C$_6$H$_5$), 109.88 (C5), 87.70 (C4'), 84.92 (C1'), 69.97 (C3'), 60.95 (C5'), 40.06 (C2'), 13.12 (C5-CH$_3$).

HR-MS (ESI): calcd for (C17H19N3NaO5)$^+$ [M+Na]$^+$: 368.1222, found: 368.1209.

Compound 21:

N-(1-((2*R*,4*S*,5*R*)-5-(((*tert*-butyldimethylsilyl)oxy)methyl)-4-hydroxytetrahydrofuran-2-yl)-5-methyl-2-oxo-1,2-dihydropyrimidin-4-yl)benzamide

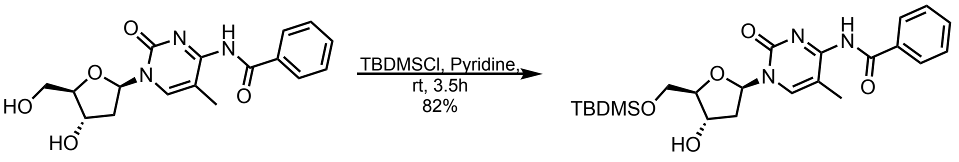

20 21

In an inert Schlenk flask under argon atmosphere compound **12** (852.1 mg, 2.47 mmol, 1.00 equiv.) was dissolved in 6.2 mL dry pyridine. TBDMSCl (483.44 mg, 3.21 mmol, 1.30 equiv.) was added and reaction stirred at RT. After 3 h again TBDMSCl (185.94 mg, 1,23 mmol, 0.50 equiv.) was added and the reaction mixture was stirred at RT over 30 min. The reaction was quenched by adding 1ml MeOH and solvent was removed under reduced pressure. The residue was dissolved in 50 mL ethyl acetate and washed with 50 mL of sat. NaHCO$_3$, water, brine. The organic layer was dried over MgSO$_4$, filtered and evaporated under reduced pressure. The resulted crude product was purified with silica gel column chromatography using MeOH and DCM as eluent system (0% to 2% MeOH/DCM). The protected product **21** was afforded as white solid (934.3mg, 82% isolated yield).

1H NMR (700 MHz, DMSO-*d*6): δ (ppm): 13.00 (s, 1H, NH), 8.23 – 8.15 (m, 2H, 4-NH-COC$_6$H$_5$), 7.77 (s, 1H, 6-H), 7.58 (t, *J* = 7.4 Hz, 1H, 4-NH-COC$_6$H$_5$), 7.49 (t, *J* = 7.5 Hz, 2H, 4-NH-COC$_6$H$_5$), 6.17 (t, *J* = 6.8 Hz, 1H, 1'-H), 5.32 (d, *J* = 4.2 Hz, 1H, 3'-OH), 4.23 (ddd, *J* = 7.4, 6.0, 3.3 Hz, 1H, 3'-H), 3.92 – 3.87 (m, 1H, 4'-H), 3.80 (ddd, *J* = 46.0, 11.5, 3.6 Hz, 2H, 5'-H), 2.24 – 2.10 (m, 2H, 2'-H), 2.02 (s, 3H, 5-CH$_3$), 0.88 (s, 9H, 5'-O-Si(CH$_3$)$_2$C(CH$_3$)$_3$), 0.09 (d, *J* = 3.8 Hz, 6H, 5'-O-Si(CH$_3$)$_2$C(CH$_3$)$_3$).

13C NMR (151 MHz, DMSO-*d*6): δ (ppm): 178.15 (CO), 158.98 (C4), 147.38 (C2), 138.16 (C6), 136.68 (C$_6$H$_5$), 132.52 (C$_6$H$_5$), 129.36 (C$_6$H$_5$), 128.29 (C$_6$H$_5$), 109.88 (C5), 87.31 (C4'), 85.12 (C1'), 70.34 (C3'), 63.13 (C5'), 40.06 (C2'), 25.78 (5'-O-Si(CH$_3$)$_2$C(CH$_3$)$_3$), 18.03 (5'-O-Si(CH$_3$)$_2$C(CH$_3$)$_3$), 13.09 (C5-CH$_3$), -5.43 (5'-O-Si(CH$_3$)$_2$C(CH$_3$)$_3$).

HR-MS (ESI): calcd for (C23H33N3NaO5Si)$^+$ [M+Na]$^+$: 482.2087, found: 482.2070.

Compound 22:

N-(1-((2*R*,4*S*,5*R*)-5-(((*tert*-butyldimethylsilyl)oxy)methyl)-4-((methylthio)methoxy)tetrahydrofuran-2-yl)-5-methyl-2-oxo-1,2-dihydropyrimidin-4-yl)benzamide

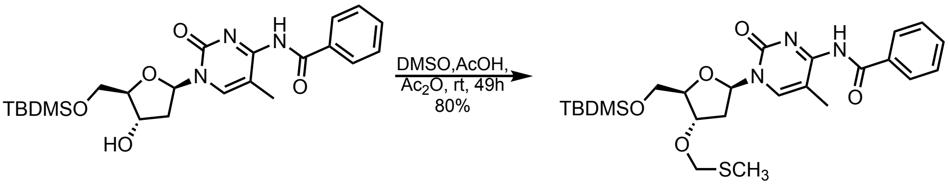

21 **22**

In an inert Schlenk flask under argon atmosphere compound **21** (916.1 mg, 1.99 mmol, 1.00 equiv.) was dissolved in 3.83 mL dry DMSO, 1.82 mL acetic acid and 5.74 mL acetic anhydride were added to the solution. The reaction was allowed to stir at RT over 49 h. Quenching of the reaction was performed by adding 50 mL saturated $NaHCO_3$ solution. The aqueous layer was washed four times with ethyl acetate and the combined organic layer was washed with saturated $NaHCO_3$ solution. The organic layer was dried over Na_2SO_4, filtered and evaporated under reduced pressure to afford crude product. Silica gel column chromatography using EtOAc and cyclohexane as eluent system (15% EtOAc/Cyclohexane) yielded product **22** as white foamy solid (826.4mg, 80% isolated yield).

1H NMR (700 MHz, DMSO-*d*$_6$) δ (ppm): 12.96 (s, 1H, NH), 8.22 – 8.15 (m, 2H, 4-NH-COC_6H_5), 7.80 – 7.75 (m, 1H, 6-H), 7.59 (t, *J* = 7.4 Hz, 1H, 4-NH-COC_6H_5), 7.50 (t, *J* = 7.6 Hz, 2H, 4-NH-COC_6H_5), 6.13 (dd, *J* = 8.2, 5.9 Hz, 1H, 1'-H), 4.73 (s, 2H, 3'-O-CH_2SCH_3), 4.42 (dt, *J* = 6.2, 2.3 Hz, 1H, 3'-H), 4.05 (td, *J* = 4.1, 2.2 Hz, 1H, 4'-H), 3.89 – 3.75 (m, 2H, 5'-H), 2.41 – 2.34 (m, 1H, 2'-H), 2.22 (ddd, *J* = 14.2, 8.3, 6.2 Hz, 1H, 2'-H), 2.10 (s, 3H, 3'-O-CH_2SCH_3), 2.03 (s, 3H, 5-CH_3), 0.89 (s, 9H, 5'-O-$Si(CH_3)_2C(CH_3)_3$), 0.10 (d, *J* = 3.5 Hz, 6H, 5'-O-$Si(CH_3)_2C(CH_3)_3$).

13C NMR (176 MHz, DMSO-*d*$_6$): δ (ppm): 178.14 (CO), 158.77 (C4), 147.43 (C2), 137.99 (C6), 136.62 (C_6H_5), 132.57 (C_6H_5), 129.38 (C_6H_5), 128.31 (C_6H_5), 110.08 (C5), 85.19 (C1'), 84.60 (C4'), 75.92 (C3'), 72.63 (3'-O-CH_2SCH_3), 63.03 (C5'), 36.56 (C2'), 25.76 (5'-O-$Si(CH_3)_2C(CH_3)_3$), 17.97 (5'-O-$Si(CH_3)_2C(CH_3)_3$), 13.27 (3'-O-CH_2SCH_3), 13.10 (C5-CH_3), -5.45 (5'-O-$Si(CH_3)_2C(CH_3)_3$), -5.47 (5'-O-$Si(CH_3)_2C(CH_3)_3$).

HR-MS (ESI): calcd for $(C25H37N3NaO5SSi)^+$ $[M+Na]^+$: 542.2121, found: 542.2103.

Compound 23:

N-(1-((2*R*,4*S*,5*R*)-4-(azidomethoxy)-5-(hydroxymethyl)tetrahydrofuran-2-yl)-5-methyl-2-oxo-1,2-dihydropyridin-4-yl)benzamide

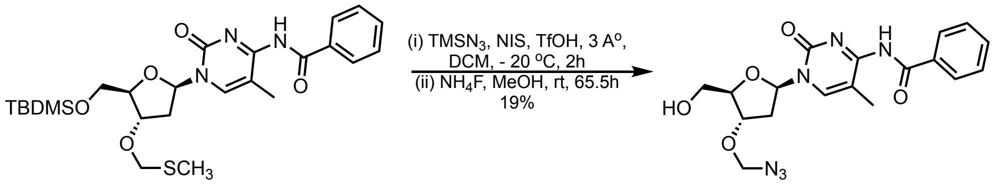

22 **23**

In an inert Schlenk flask under argon atmosphere compound **22** (373.4 mg, 0.718 mmol, 1.00 equiv.) with 3 A° MS was dissolved in dry 7.2 mL DCM. $TMSN_3$ (285 µL, 2.16 mmol, 3.00 equiv.) was added and stirred at RT over 10 min. The reaction mixture was cooled to - 20 ºC, NIS (323.28 mg, 1.44 mmol, 2.00 equiv.) and TfOH (95.4 µL, 1.08 mmol, 1.50 equiv.) were added. The reaction mixture was allowed to stir under same conditions over 2h. Quenching of the reaction was performed by adding 5 mL saturated $NaHCO_3$ solution at 0 ºC, filtered, and the filtrate was mixed with 40 mL DCM washed with saturated $Na_2S_2O_3$ and brine. The organic layer was dried over $MgSO_4$, filtered and evaporated under reduced pressure. The residue was dissolved in 7.2 mL dry MeOH and NH_4F (319.31 mg, 8.62 mmol, 12.00 equiv.) was added. The reaction was stirred at RT over 23 h. LC-MS suggested presence of 5'-OH protected product, again NH_4F (159.66 mg, 4.31 mmol, 6.00 equiv.) was added to the reaction mixture and allowed to stir under same condition over 42.5h. The solvent was removed under reduced pressure and the residue was suspended in 50 mL milli-Q water. The aqueous solution was extracted with 50 mL DCM four times and the combined organic layer was dried over Na_2SO_4, filtered and evaporated under reduced pressure. The crude product was purified with silica gel column chromatography using EtOAc and Cyclohexane as eluent system MeOH and DCM as eluent (1:400-1:200-1: 150 of MeOH : DCM). The product **23** was afforded as white solid (54.8 mg, 19% isolated yield).

1H NMR (600 MHz, Chloroform-*d*): δ (ppm): 12.98 (s, 1H, NH), 8.22 – 8.17 (m, 2H, 4-NH-COC_6H_5), 8.04 (s, 1H, 4-NH-COC_6H_5), 7.59 (t, *J* = 7.3 Hz, 1H, 4-NH-COC_6H_5), 7.50 (t, *J* = 7.6 Hz, 2H, 4-NH-COC_6H_5), 6.15 (t, *J* = 6.7 Hz, 1H, 1'-H), 5.24 (t, *J* = 5.2 Hz, 1H, 5'-OH), 4.87 (s, 2H, 3'-O-CH_2N_3), 4.40 (dt, *J* = 6.4, 3.2 Hz, 1H, 3'-H), 4.03 (q, *J* = 3.6 Hz, 1H, 4'-H), 3.72 – 3.60 (m, 2H, 5'-H), 2.42 – 2.29 (m, 2H, 2'-H), 2.03 (s, 3H, 5-CH_3).
13C NMR (151 MHz, Chloroform-*d*): δ (ppm): 178.14 (CO), 158.96 (C4), 147.48 (C2), 138.70 (C6), 136.69 (C_6H_5), 132.55 (C_6H_5), 129.37 (C_6H_5), 128.32 (C_6H_5), 110.03 (C5), 85.13 (C4'), 84.85 (C1'), 81.07 (3'-O-CH_2N_3), 77.96 (C3'), 60.90 (C5'), 37.19 (C2'), 13.13 (C5-CH_3).
HR-MS (ESI): calcd for $(C18H18N8NaO5)^+$ $[M+Na]^+$: 423.1393, found: 423.1380.

Compound 24: 3'-O-azidomethyl-5mdCMP:
((2*R*,3*S*,5*R*)-5-(4-amino-5-methyl-2-oxopyrimidin-1(2*H*)-yl)-3-(azidomethoxy)tetrahydrofuran-2-yl)methyl dihydrogen phosphate

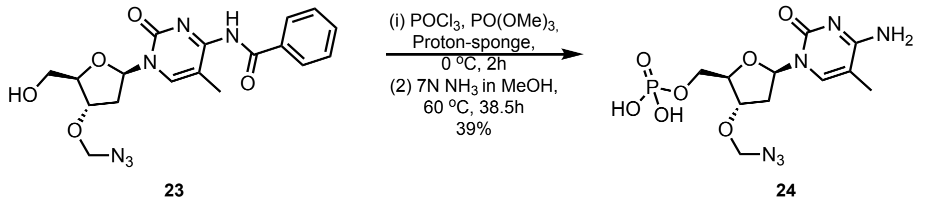

Compound **23** (36.20 mg, 90.41 µmol, 1.00 equiv.) and proton sponge (116.26 mg, 0.542mmol, 6.00 equiv.) were dried over high vacuum for 30 min. Under argon atmosphere mixture was dissolved in 0.23 mL trimethyl phosphate and cooled at 0 ºC. $POCl_3$ (33.7 µL, 0.362 mmol, 4.00 equiv.) was added dropwise and reaction was stirred over 2 h. Quenching of reaction was performed by adding 2.9 mL of ice-cold 1.0M TEAA buffer (pH= 7.6) and the turbid solution was allowed to stir over 1 h at 0 ºC. Solvent was evaporated under reduced pressure and filter column (silica gel column chromatography) was used to remove the proton sponge using 0 to

5%MeOH/DCM/2%TEA eluent system, the rest residue was eluted with max 15%MeOH/DCM/2%TEA. Solvent was removed under reduced pressure. 6.65 mL 7N NH_3 in MeOH was added to the residue and was stirred at 60 ºC over 38.5 h. After removing solvent under reduced pressure, the crude was purified with HPLC using VP nucleodur C18 column ((eluent A and B). Purified product was lyophilized to obtain 3'-O-azidomethyl-8-oxo-dAMP **16** as a TEA salt with 1:0.935:0.21 (compound **24**: TEA: AA and 78wt%) mol ratio (17.2 mg, 35.574 µmol, 39% with TEA salt).

1H NMR (600 MHz, D_2O): δ (ppm): 7.82 (d, *J* = 1.5 Hz, 1H, 6-H), 6.35 (dd, *J* = 8.1, 6.0 Hz, 1H, 1'-H), 4.88 (s, 2H, 3'-O-CH_2N_3), 4.60 (dt, *J* = 5.6, 2.5 Hz, 1H, 3'-H), 4.37 (p, *J* = 3.0 Hz, 1H, 4'-H), 4.13 – 4.06 (m, 2H, 5'-H), 3.21 (q, *J* = 7.3 Hz, 5.5H, Et_3NH^+), 2.57 (ddd, *J* = 14.2, 6.0, 2.5 Hz, 1H, 2'-H), 2.34 (ddd, *J* = 14.3, 8.2, 6.1 Hz, 1H, 2'-H), 2.01 (d, *J* = 1.1 Hz, 3H, 5-CH_3), 1.95 – 1.92 (m, 0.62H, CH_3COO^-), 1.29 (t, *J* = 7.3 Hz, 8.42H, Et_3NH^+).
31P NMR (243 MHz, D_2O): δ (ppm): 0.38.
13C NMR (151 MHz, D_2O): δ (ppm): 165.42 (C2), 156.68 (C4), 138.72 (C6), 105.01 (C5), 85.83 (C1'), 83.77 (d, C4'), 81.48 (3'-O-CH_2N_3), 78.93 (C4'), 64.65 (d, C5'), 46.65 (Et_3NH^+), 37.44 (C2'), 12.27 (C5-CH_3), 8.20 (Et_3NH^+).
HR-MS (ESI): calcd for $(C_{11}H_{16}N_6O_7P)^-$ $[M-H]^-$: 375.0824, found: 375.0812.

Compound 25: 3'-O-azidomethyl-5mdCMP morpholidate:
((2*R*,3*S*,5*R*)-5-(4-amino-5-methyl-2-oxopyrimidin-1(2*H*)-yl)-3-(azidomethoxy)tetrahydrofuran-2-yl)methyl hydrogen morpholinophosphonate

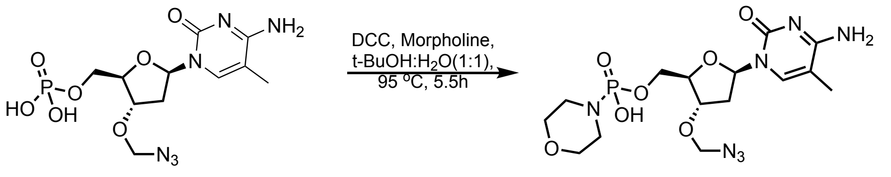

3'-O-azidomethyl-5mdCMP **24** with TEA salt (13.0 mg, 26.89 µmol, 1.00 equiv.) was dissolved in 0.5 mL milli-Q water and the pH of the solution was adjusted 3-4 by adding 1M HCl. Anhydrous morpholine (11.60 µL, 0.134 mmol, 5.00 equiv.) dissolved in 0.5 mL *t*-BuOH was added dropwise. The reaction mixture was allowed to stir at RT over 30 min, and warmed to 95 ºC. DCC (27.75 mg, 0.134 mmol, 5.00 equiv.) dissolved in 1 mL *t*-BuOH was added dropwise over 2 h under same condition. Solution was further stirred over 3.5 h at 95 °C. The solvents were evaporated under reduced pressure, and resuspended in 1 mL of ice-cold water. Dicyclohexylurea precipitates were removed by filtration of the suspension using 0.45 µm HPLC Filter Chromafil. Filtrate was purified with HPLC using VP nucleodur C18 column (eluent A and B) and lyophilized to obtain 3'-O-azidomethyl-5mdCMP morpholidate **25** as a TEA salt. The compound **25** was directly used in next step for triphosphate synthesis.

HR-MS (ESI): calcd for $(C_{15}H_{23}N_7O_7P)^-$ $[M-H]^-$: 444.1402, found: 444.1393.

Compound 26: 3'-O-azidomethyl-5mdCTP: ((2*R*,3*S*,5*R*)-5-(4-amino-5-methyl-2-oxopyrimidin-1(2*H*)-yl)-3-(azidomethoxy)tetrahydrofuran-2-yl)methyl triphosphate

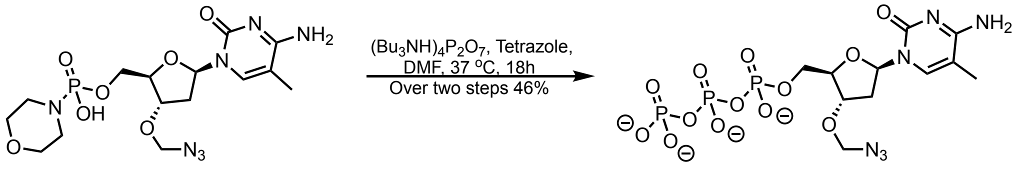

3'-O-azidomethyl-5mdCMP morpholidate **25** was co-evaporated three times with 0.5 mL toluene and dried over high vaccum overnight. To the dried morpholidate tributylammonium pyrophosphate (59.0 mg, 107.55 µmol, 4.00 equiv.) was added and were dried over high vacuum over 30min. 0.45M Tetrazole (300 µL, 134.44 µmol, 5.00 equiv.) and dry DMF (860 µL) were added to the dry mixture under argon atmosphere. The reaction mixture was stirred at 37 °C over 18 h and solvent was removed by lyophilizing the reaction mixture. The crude was first purified with HPLC using Ion-exchange column with eluents C and D. Purified fraction was lyophilized and salt exchange was performed with VP nucleodur C18 column using eluents A and B. Purified triphosphate was lyophilized to afford triphosphate **26** as a TEA salt with 1:1.985:0.14 (compound **26**: TEA: AA and 72wt%) mol ratio over two steps (9.2 mg, 12.34 µmol, 46% with TEA salt).

1H NMR (700 MHz, D_2O): δ (ppm): 7.80 (d, *J* = 1.2 Hz, 1H, 6-H), 6.36 (dd, *J* = 8.6, 5.8 Hz, 1H, 1'-H), 4.93 – 4.83 (m, 2H, 3'-O-CH_2N_3), 4.65 (dt, *J* = 6.0, 2.2 Hz, 1H, 3'-H), 4.41 – 4.38 (m, 1H, 4'-H), 4.26 – 4.21 (m, 2H, 5'-H), 3.20 (q, *J* = 7.4 Hz, 11.91H), 2.56 (ddd, *J* = 14.3, 5.8, 2.1 Hz, 1H, 2'-H), 2.34 (ddd, *J* = 14.3, 8.6, 5.9 Hz, 1H, 2'-H), 2.02 (s, 3H, 5-CH_3), 1.92 (s, 0.42H), 1.28 (t, *J* = 7.3 Hz, 18.16H).
31P NMR (283 MHz, D_2O) with 1H decoupling: δ (ppm): -9.79 (br s), -11.65 (d, *J* = 19.9 Hz), -23.03 (t, *J* = 19.9 Hz).
31P NMR (243 MHz, D_2O) without 1H decoupling: δ (ppm): -9.79 (br s), -11.66 (dd, *J* = 19.9, 5.6 Hz), -23.03 (t, *J* = 19.9 Hz).
13C NMR (176 MHz, D_2O): δ (ppm): 165.92 (C2), 157.36 (C4), 138.45 (C6), 105.20 (C5), 85.77 (C1'), 83.63 (d, C4'), 81.56 (3'-O-CH_2N_3), 79.37 (C3'), 65.65 (d, C5'), 46.63 (Et_3NH^+), 37.48 (C2'), 12.36 (C5-CH_3), 8.19 (Et_3NH^+).
HR-MS (ESI): calcd for $(C11H18N8O13P3)^-$ $[M-H]^-$: 535.015, found: 535.0139.

NMR Spectra

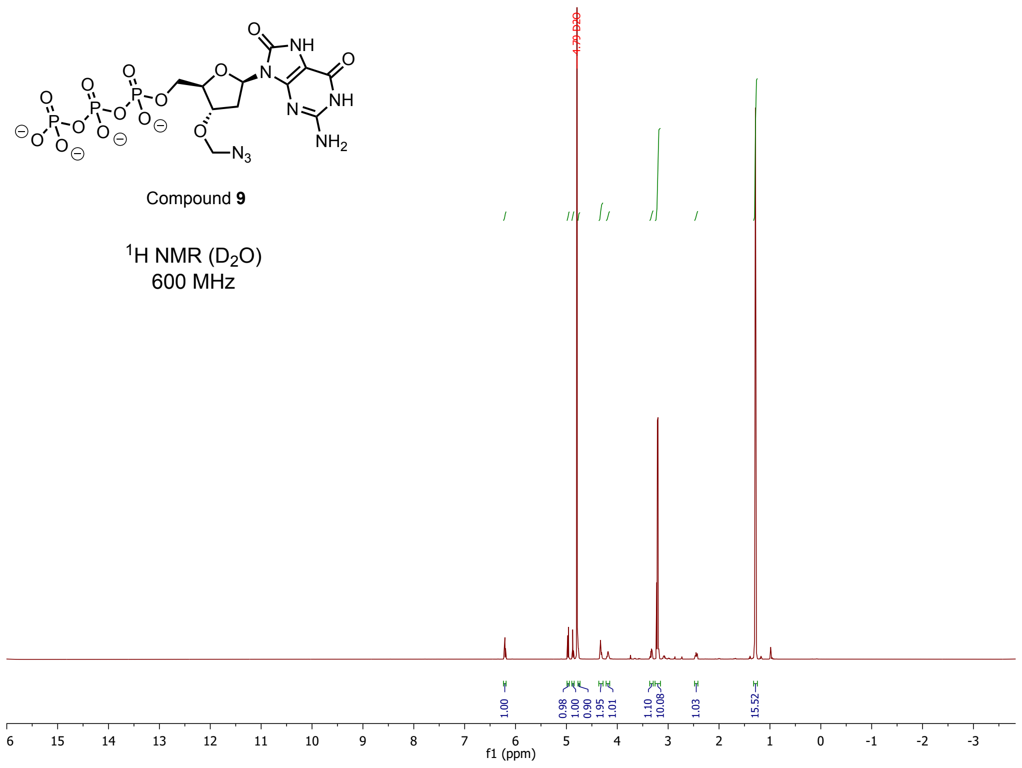
Compound **9**
^1H NMR (D_2O)
600 MHz
4.79 D2O
1.00
0.98
1.00
0.90
1.95
1.01
1.10
10.08
1.03
15.52
f1 (ppm)

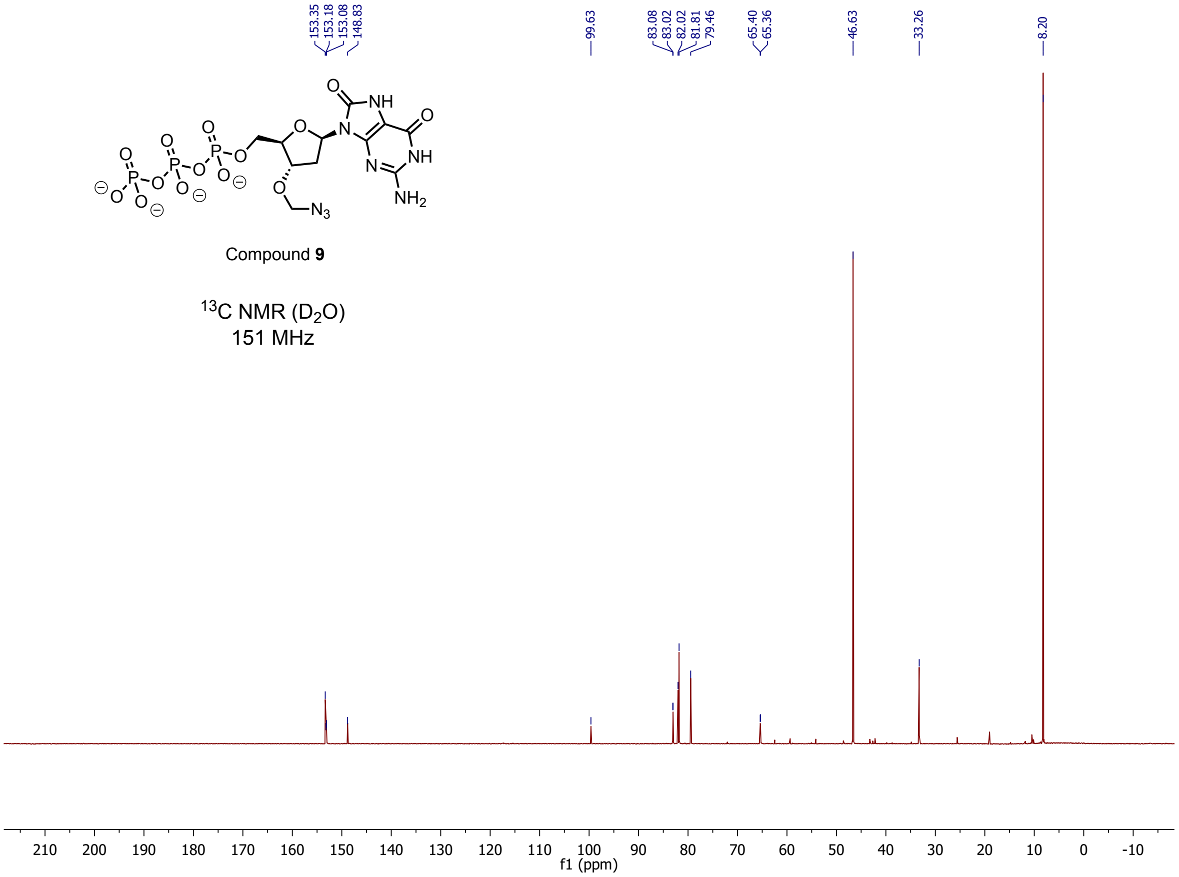
153.35
153.18
153.08
148.83
99.63
83.08
83.02
82.02
81.81
79.46
65.40
65.36
46.63
33.26
8.20
Compound **9**
^{13}C NMR (D_2O)
151 MHz
f1 (ppm)

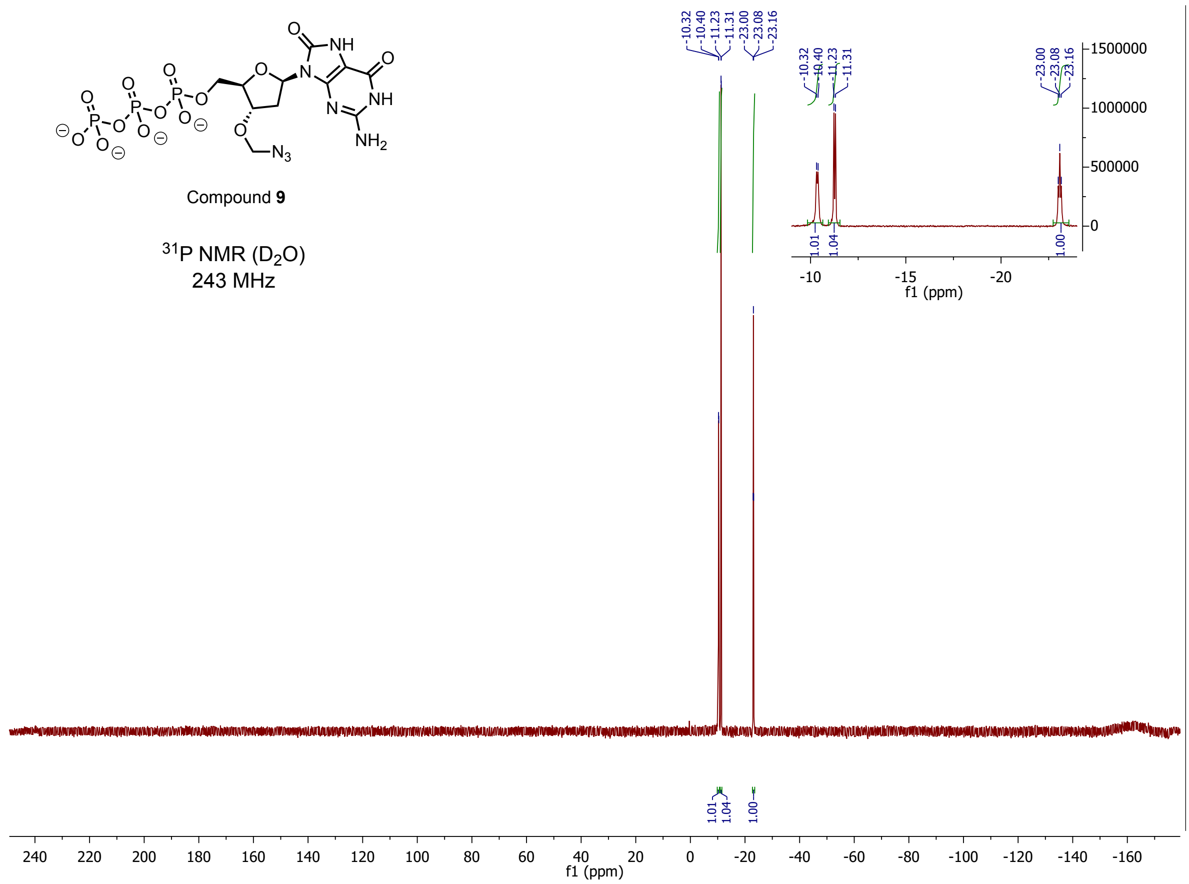
Compound **9**
^{31}P NMR (D_2O)
243 MHz
-10.32
-10.40
-11.23
-11.31
-23.00
-23.08
-23.16
1.01
1.04
1.00
f1 (ppm)

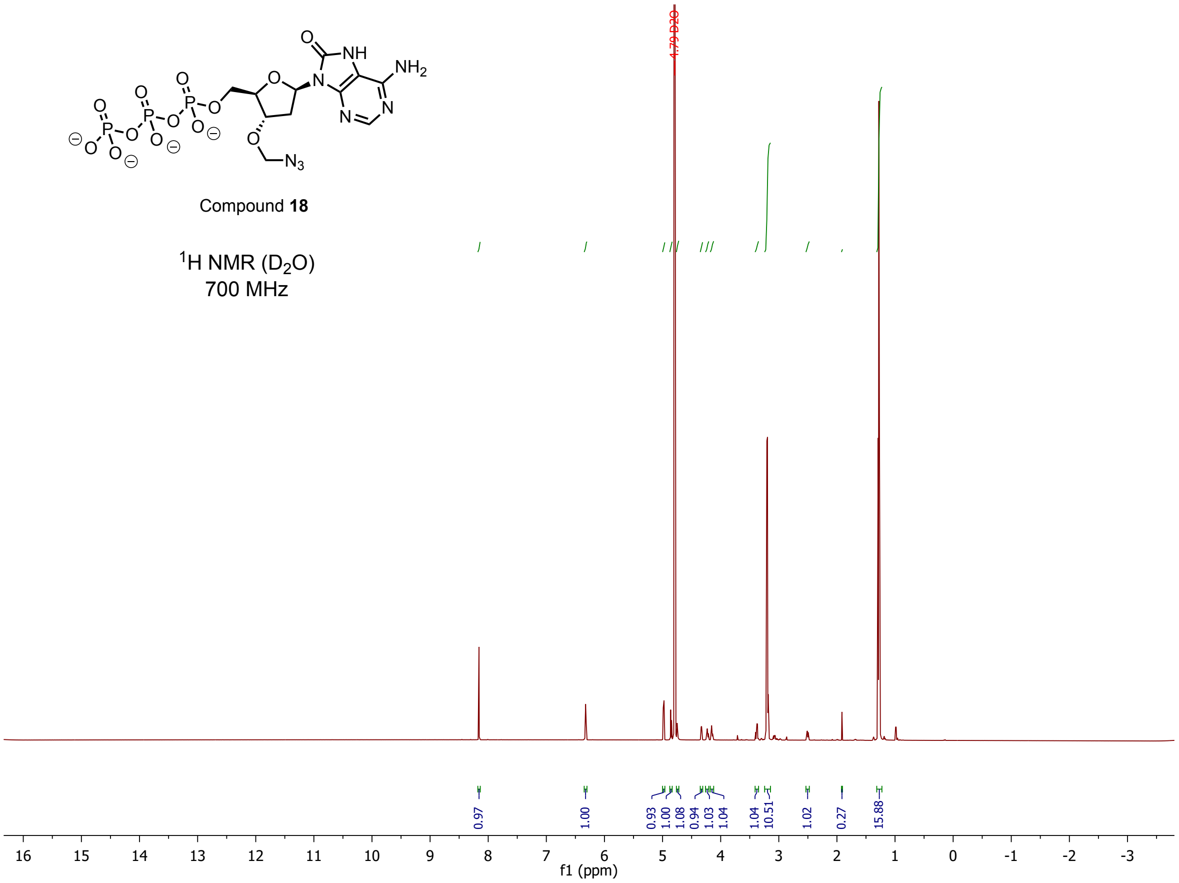
Compound **18**
^{1}H NMR (D_2O)
700 MHz
4.79 D2O
0.97
1.00
0.93
1.00
1.08
0.94
1.03
1.04
1.04
10.51
1.02
0.27
15.88
f1 (ppm)

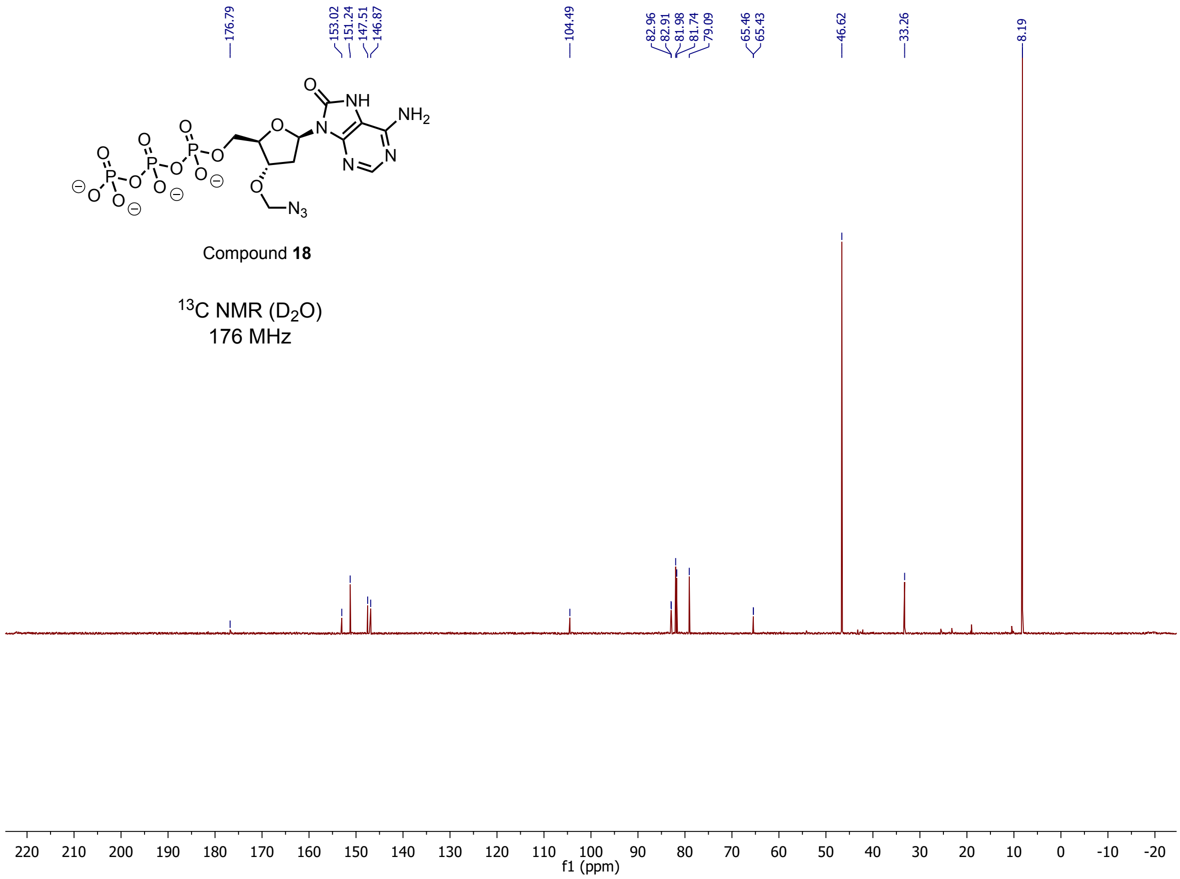
176.79
153.02
151.24
147.51
146.87
104.49
82.96
82.91
81.98
81.74
79.09
65.46
65.43
46.62
33.26
8.19
Compound 18
^{13}C NMR (D_2O)
176 MHz
f1 (ppm)

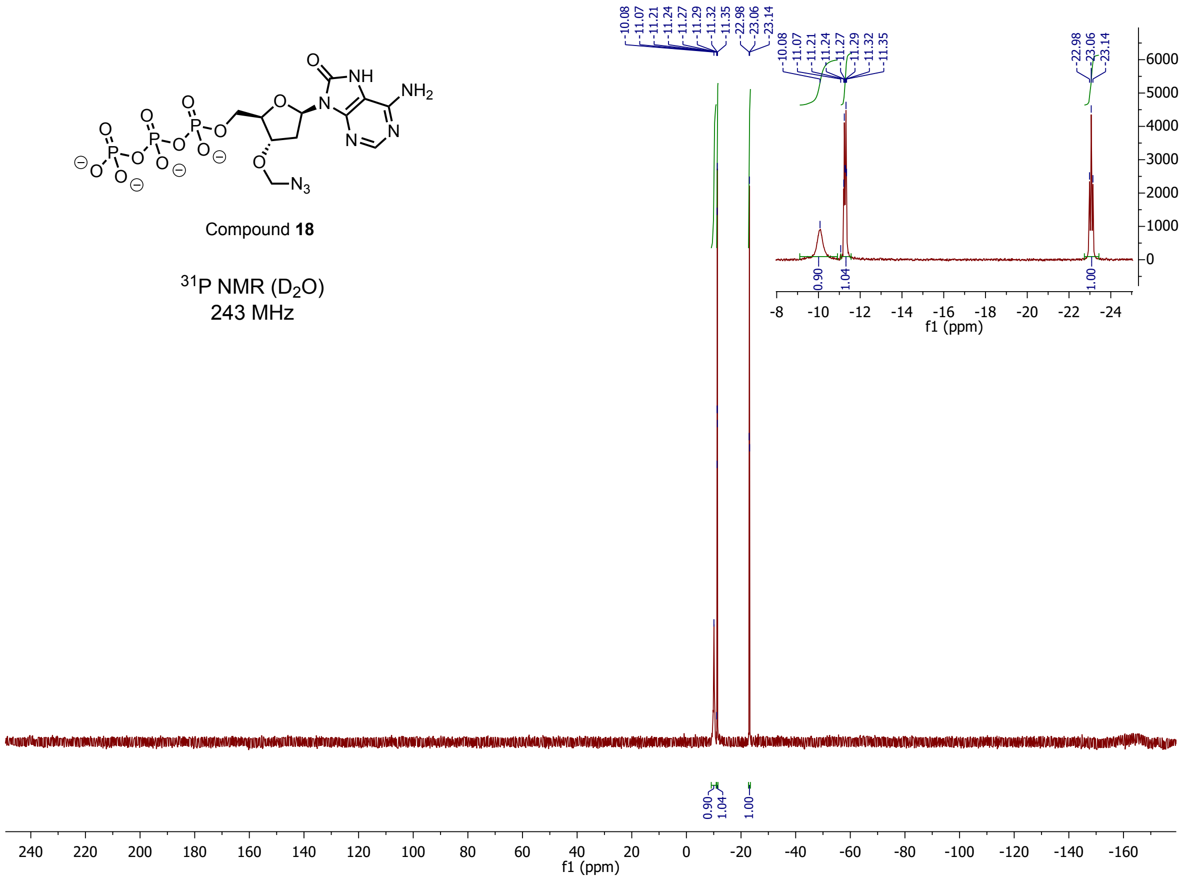
-10.08
-11.07
-11.21
-11.24
-11.27
-11.29
-11.32
-11.35
-22.98
-23.06
-23.14
Compound 18
^{31}P NMR (D_2O)
243 MHz
0.90
1.04
1.00
f1 (ppm)

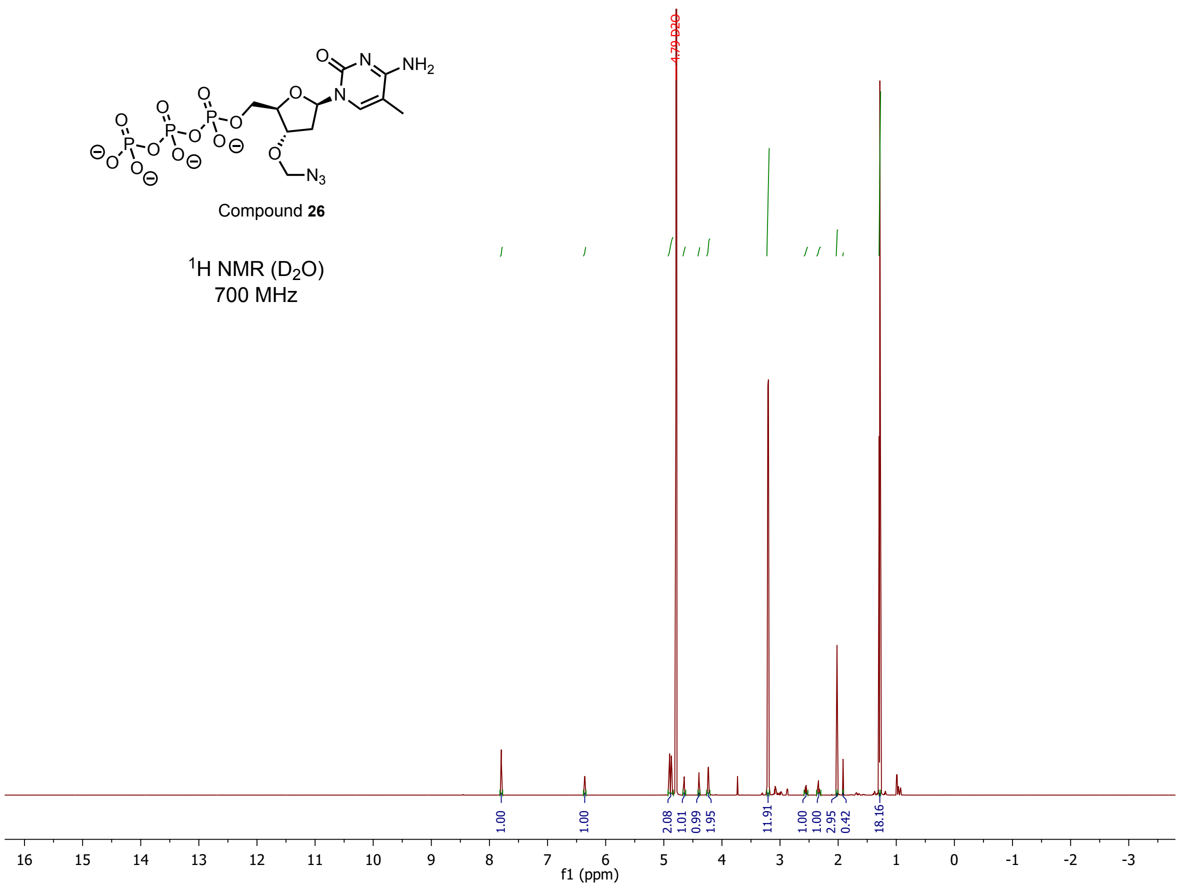

Compound 26
^{1}H NMR (D_2O)
700 MHz
4.79 D2O
1.00
1.00
2.08
1.01
0.99
1.95
11.91
1.00
1.00
2.95
0.42
18.16
f1 (ppm)

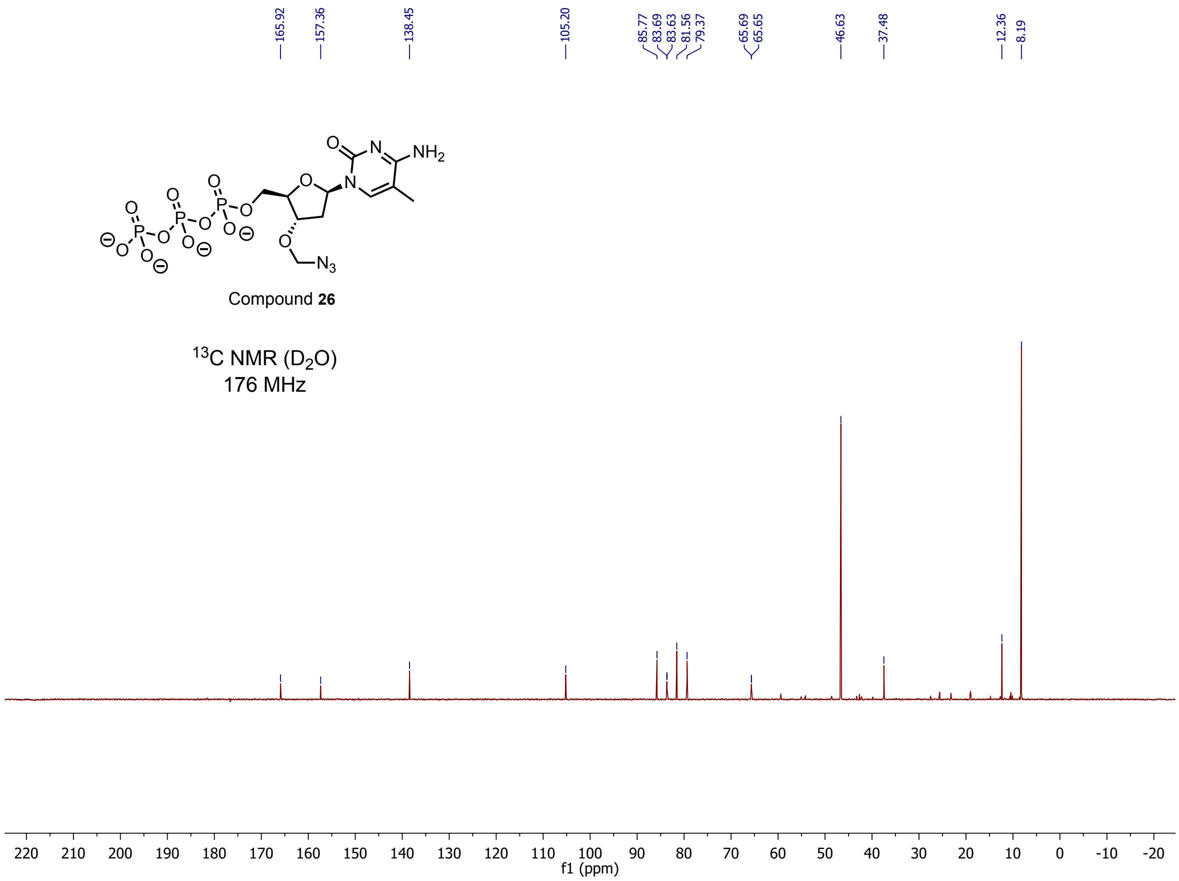

165.92
157.36
138.45
105.20
85.77
83.69
83.63
81.56
79.37
65.69
65.65
46.63
37.48
12.36
8.19
Compound 26
^{13}C NMR (D_2O)
176 MHz
f1 (ppm)

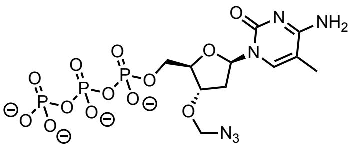

Compound **26**

^{31}P NMR (D_2O)
243 MHz

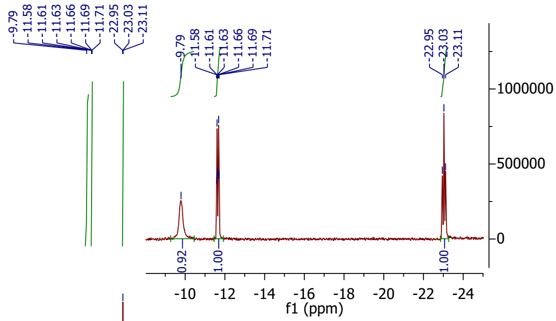

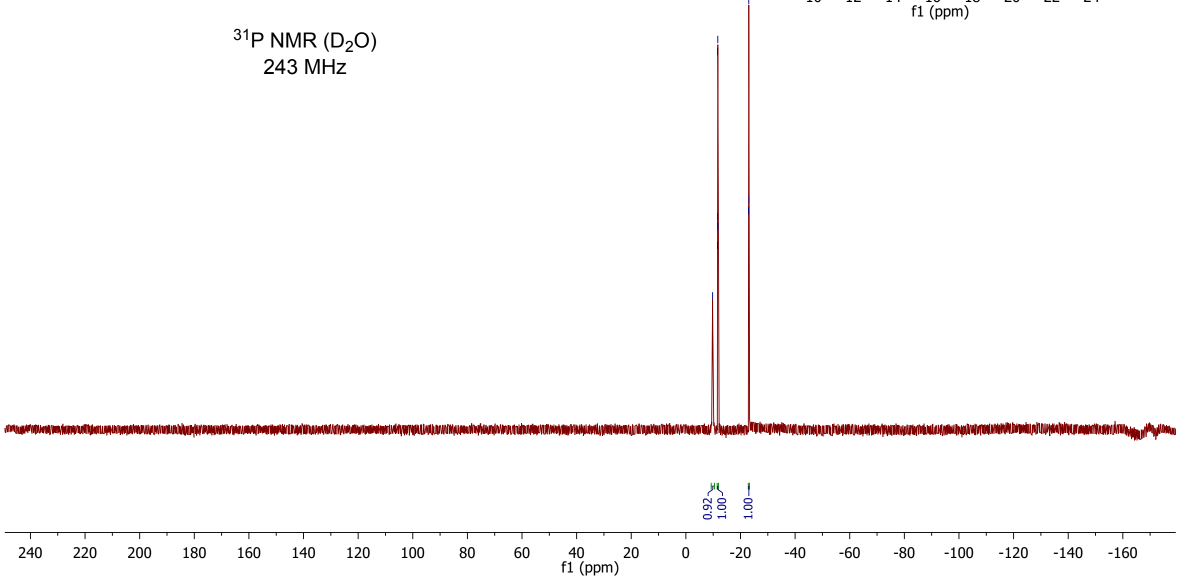

Supplement: Supplementary file 1 — Supporting File: advs75917‐sup‐0001‐SuppMat.pdf. [file ADVS-9999-e75917-s001.pdf]
